# Short and Modular Synthesis of Substituted 2-Aminopyrroles

**DOI:** 10.1021/acs.orglett.1c01345

**Published:** 2021-04-30

**Authors:** Raquel Diana-Rivero, Beate Halsvik, Fernando García Tellado, David Tejedor

**Affiliations:** † Instituto de Productos Naturales y Agrobiología, CSIC, Astrofísico Francisco Sánchez 3, 38206 La Laguna, Tenerife, Spain; ‡ Doctoral and Postgraduate School, Universidad de La Laguna, Santa Cruz de Tenerife, Spain; § Department of Chemistry, University of Bergen, Allégaten 41, NO-5007 Bergen, Norway

## Abstract

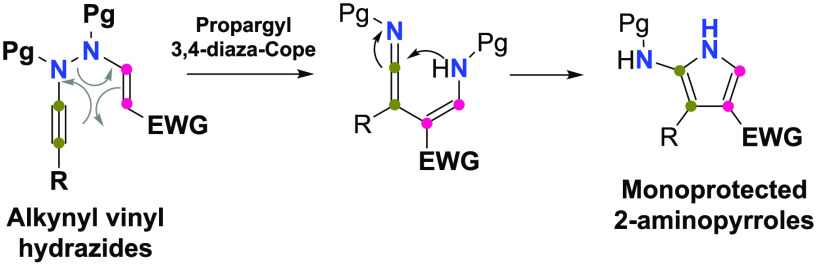

We herein describe a simple and metal-free domino methodology to synthesize 2-aminopyrroles from alkynyl vinyl hydrazides. The domino reaction involves a novel propargylic 3,4-diaza-Cope rearrangement and a tandem isomerization/5-exo-dig N-cyclization reaction. By using this approach, a number of 2-aminopyrroles with diverse substituents have been prepared.

The 2-aminopyrrole ring constitutes an architectural motif present in many bioactive compounds spanning a wide set of pharmacological activities.^1^ Selected examples include multisubstituted pyrroles **I** and **II**, which are inhibitors of mitogen-activated protein kinase enzymes (MEKs)^1a^ and metallo-β-lactamases (MBL),^1b^ respectively, or the heterofused pyrrole **III**, a modulator of B-cell lymphoma 2 (Bcl-2) family members^1c^ (Figure 1). Besides these therapeutic applications, 2-aminopyrroles have found use as molecular platforms in the synthesis of analogues of purine bases.^2^

**Figure 1:**
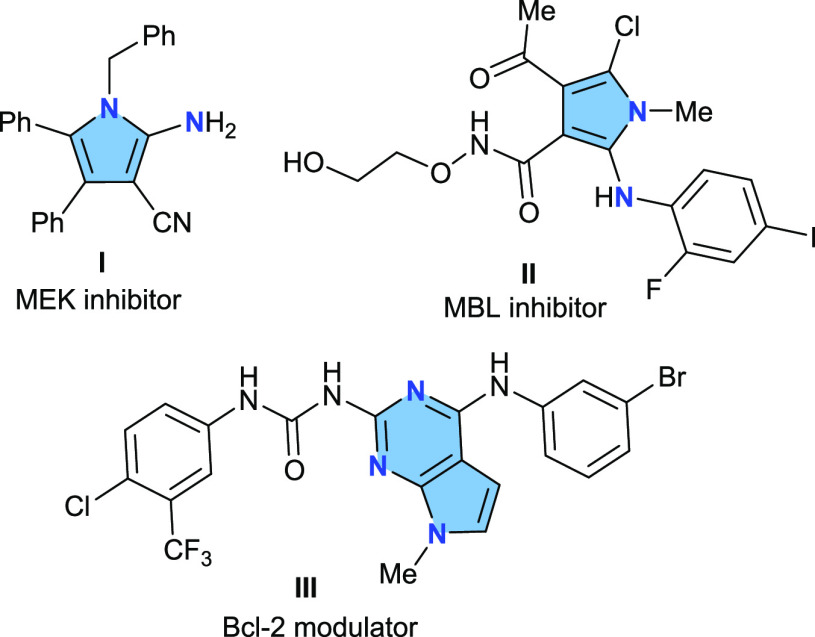
Bioactive 2-aminopyrroles.

These properties are made of these 5-membered heterocycle recurrent synthetic targets.^3^ Although the synthesis of pyrroles is well established and a good number of efficient methodologies based on the classical Knorr,^4^ Paal–Knorr,^5^ and Hantzsch^6^ reactions are already available,^7^ they are not easily adapted to the synthesis of 2-aminopyrroles. These limitations have fueled the development of novel synthetic strategies to gain access to these substituted pyrroles. They essentially rely on three main types: (1) the multicomponent approach using nitriles or isocyanides,^3b,3d,8^ (2) the transition-metal-catalyzed cycloisomerization of alkynes and allenes,^3c,9^ and (3) miscellaneous domino (cascade) approaches.^10^

Over the last years, our group has been focused on the design and development of domino processes based on the propargyl Claisen rearrangement of propargyl enol ethers (PVEs).^11^ In a previous work,^12^ we found that the microwave irradiation of PVEs **1** bearing an electron-withdrawing group (EWG) at the propargylic position led to furans **3** (Scheme 1a). This conversion took place through a domino process involving the propargyl Claisen rearrangement of **1** and the tandem enolization/5-exo-dig O-cyclization of the β-allenal intermediate **2**. Inspired by this result, we envisioned that this process could be applied toward the preparation of 2-aminopyrroles if a convenient *N*-alkynyl, *N*′-vinyl hydrazide platform (AVH) such as **4** could host the domino reaction (Scheme 1b). The domino reaction should be triggered by the propargylic 3,4-diaza Cope rearrangement of the AVH platform. Surprisingly, there are no precedents in the literature for this sigmatropic rearrangement even though the 3,4-diaza Cope rearrangement of hydrazines is an important reaction in organic synthesis.^13^ The Fischer^14a^ synthesis of indole and the Piloty^14b−14d^ synthesis of pyrrole constitute iconic examples. We envisioned that the lower BDE of the N–N bond (167 kJ/mol) compared with the C–O bond (358 kJ/mol) would reduce the energy barrier for the sigmatropic rearrangement, and in consequence, it could be a feasible process. In addition, we also expected that the electronic configuration of the ethenimine-enamine intermediate **6** should favor the required 5-exo-dig N-cyclization, funneling the whole transformation toward the 2-aminopyrrole **7**. We report herein the results of this study and its implementation as a synthetic strategy to access polysubstituted 2-aminopyrroles.

**Scheme 1:**
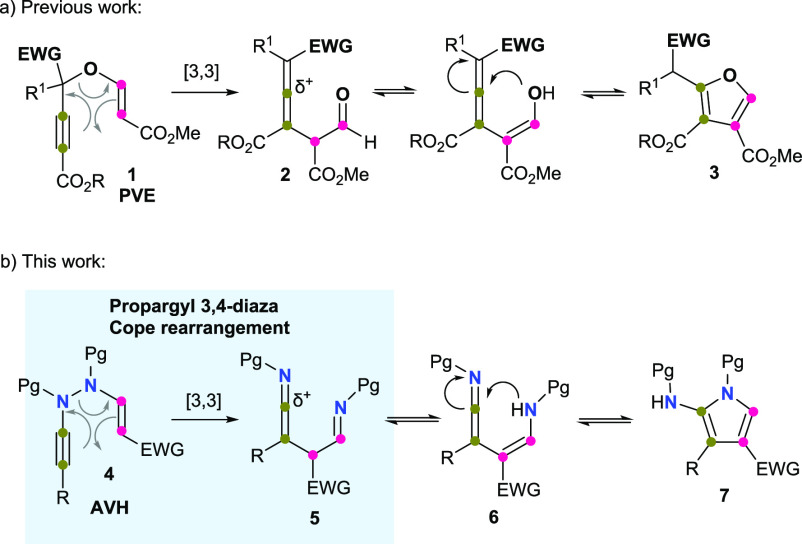
Strategy for the Domino Synthesis of 2-Aminopyrroles

In order to test the feasibility of our hypothesis, we first needed to prepare the previously unknown AVHs. Based on our own experience and the well-established synthesis of PVEs from alcohols catalyzed by DABCO,^15^ we envisioned that the incorporation of the vinyl functionality, and thus the synthesis of the AVH platforms from the corresponding *N*-alkynyl hydrazides,^16^ could be realized through the same protocol (Scheme 2). To our delight, the reaction of hydrazides **9** with activated alkynes **10** (1.1 equiv) in the presence of catalytic amounts of DABCO (10 mol %) led to AVHs **4** with an excellent average yield. These conditions were considered satisfactory, and they were not further optimized except for the conjugated amide, which required more time (16 h) and DABCO (50 mol %) to deliver the corresponding AVH **4m** in good yield (86%).

**Scheme 2:**
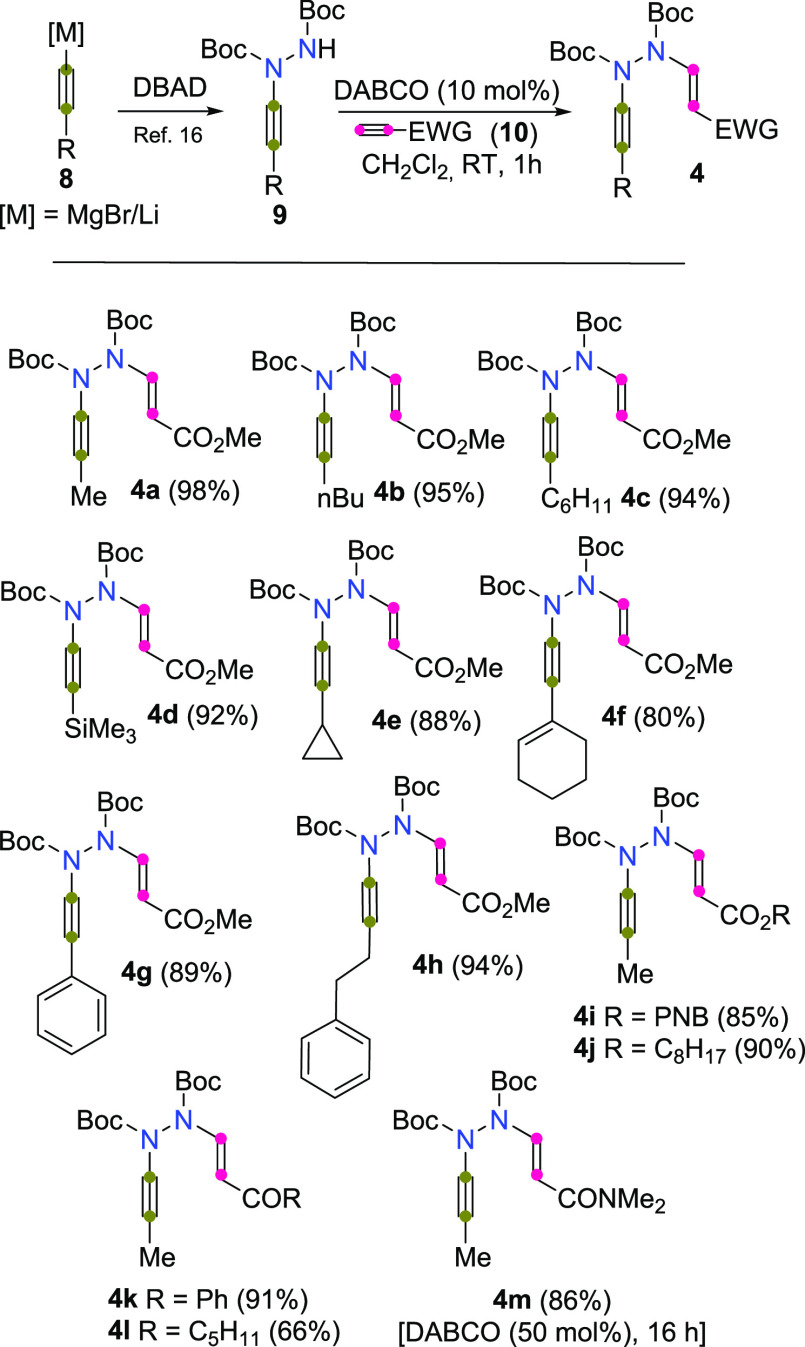
*N*-Alkynyl *N′*-Vinyl Hydrazides **4** Used in This Study DBAD = Di-*tert*-butylazodicarboxylate. DABCO = 1,4-diazabicyclo[2.2.2]octane. PNB = *p*-nitrobenzyl.

Next, we investigated if AVHs **4** could indeed undergo the expected domino transformation triggered by the propargyl 3,4-diaza Cope rearrangement (Scheme 1). AVH **4a** was taken as the model platform to study the domino reaction. According to our predictions, heating a solution of **4a** in toluene for 24 h under reflux conditions resulted in the formation of the expected 2-aminopyrrole **7a** in 17% yield (Scheme 3, entry 1). To our surprise, we also obtained the 2-aminopyrroles **11a** (37%) and **12a** (31%), which incorporated different N-protection group patterns on their structures. This result suggests that the original protection of both nitrogen atoms is modified along the reaction pathway. A number of experiments were then conducted to study the outcome of the reaction. We first found that the increase of the reaction time from 24 to 72 h favored the formation of **12a** (50%) over **7a** (4%) and **11a** (26%) (Scheme 3, entry 2). Next, we performed the reaction in refluxing xylenes (24 h). It was expected that an increase in the reaction temperature should increase the rate of the domino reaction and favor the protecting group translocation (Scheme 3, entry 3). Pleasantly, under these conditions, only the monoprotected 2-aminopyrrole **12a** was obtained in an excellent 82% yield. Furthermore, TLC control showed the sequential appearance and disappearance of products **7** and **11** and the progressive formation of **12**. It is worthy to note that under these conditions the translocation and elimination of one of the two *N*-Boc groups along the reaction pathway allow obtaining a single 2-aminopyrrole molecule endowed with an *N*-Boc-protected 2-amino group and an unsubstituted pyrrole nitrogen, which will be important for further selective synthetic transformations on these molecules and may additionally provide important analogues for SAR studies. The scope of the reaction was studied using the rest of AVH **4b**–**h** (Scheme 3, entries 4–20). In general, the reaction manifold tolerated different substituents at the alkyne moiety, including alkyl, cycloalkyl, and aryl groups.

**Scheme 3:**
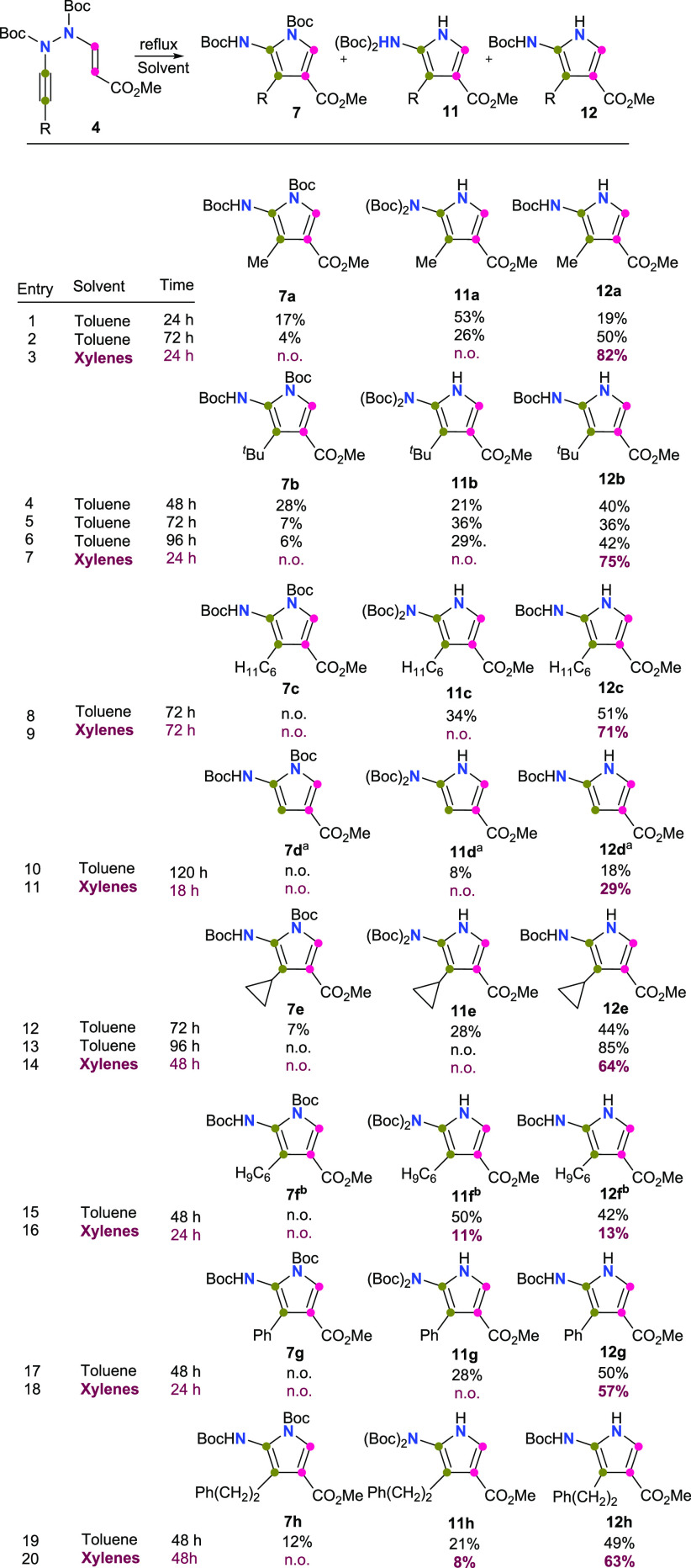
Synthesis of 2-Aminopyrroles **7**, **11**, and **12** from AVHs **4** The silyl group of AVH **4d** is lost. Cyclohex-1-en-1-yl. Abbreviations: n.o. = not observed.

The reaction time required for the whole transformation of **4** into **12** was substituent dependent, spanning from 24 h for **4a**, **4b**, and **4g** to 72 h for **4c** (entries 3, 7, 18, and 9, respectively). The cyclohexenyl-substituted AVH **4f** could not be selectively transformed into the corresponding monoprotected derivative **12f** (entries 15 and 16). Mixtures of **11f** and **12f** were consistently obtained in both toluene and xylenes, although better yields were attained in the first case. Prolonged heating in xylenes afforded decomposition of intermediates and a serious decrease in the yield (entry 16). In the case of the AVH **4h**, the replacement of toluene by xylenes decreased the yield of the reaction (82% vs 71%), and it could not entirely funnel the reaction toward the derivative **12h** (entries 19 and 20). Although mixtures of **11h** and **12h** were obtained in both cases, the proportion of **12h** increased in xylenes up to 63% at the expense of a decrease of **11h** from 21% to 8% (entry 21). Finally, in the case of AVH **4d**, the trimethylsilyl substituent did not tolerate a prolonged reflux in toluene (entry 10) or xylene (entry 11). After 120 h of heating in toluene under reflux conditions, **4d** afforded a mixture of **11d** (8%) and **12d** (18%), with complete loss of the silyl group in both structures (R = H). Under xylene reflux conditions (18 h), **4d** provided **12d** in 29% yield.

The tolerance of the reaction with regard to the nature of the electron-withdrawing group at the enamine was explored with the AVHs **4i**–**m**, featuring different ester (**4i**–**j**), ketone (**4k**–**l**), and amide (**4m**) groups (Scheme 4). All of them delivered the corresponding 2-aminopyrroles **12i**–**m** although with different efficiency. AVHs **4i**–**j** armed with an ester group afforded the corresponding 2-aminopyrroles **12i**–**j** in moderate-to-good yields (59% and 78%, respectively). On the other hand, AVHs endowed with an aliphatic ketone (**4l**) or a tertiary amide group (**4m**) gave the corresponding products **12l** or **12m** in moderate yields (47% and 40%, respectively). Unfortunately, the efficiency of the reaction manifold was seriously altered when an aromatic ketone was incorporated into the AVH (**12k**, 26%).

**Scheme 4:**
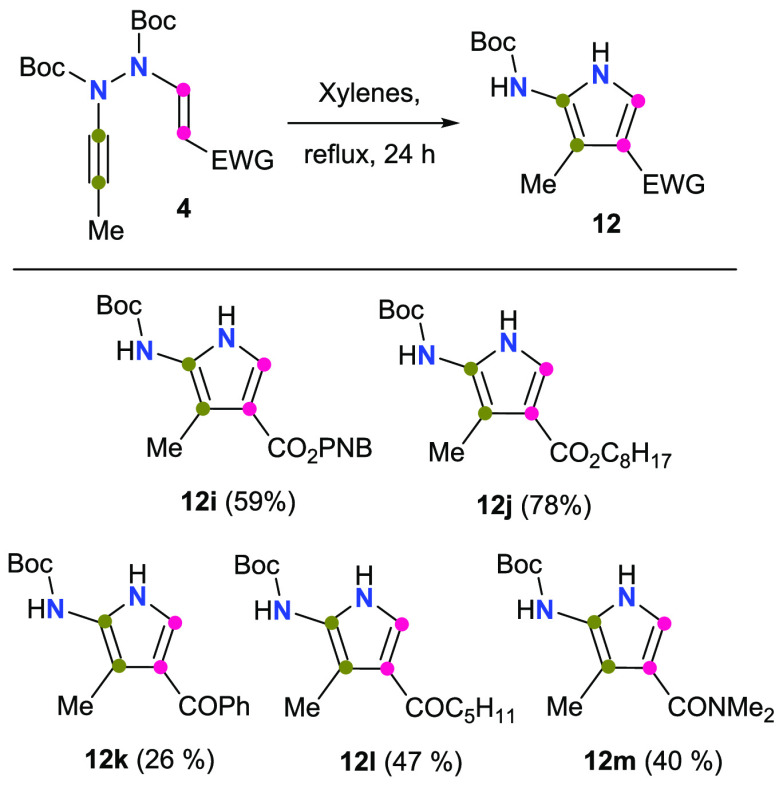
Electron-Withdrawing Group Tolerance PNB = *p*-nitrobenzyl.

Overall, these experimental results suggest that a migration/loss of one of the two *N*-Boc groups is likely involved in the domino process and that it is temperature dependent (Figure 2). To the best of our knowledge, the migration of the BOC group from the pyrrole nitrogen atom to the exocyclic carbamate nitrogen is unprecedented, and it probably arises from the different chemical reactivity of both nitrogen atoms and their spatial proximity. The experimental results are consistent with the BOC group migration occurring once the sigmatropic rearrangement has been accomplished. The accumulation of derivative **11** in the reaction medium seems to point out that the elimination process is the energetically most demanding step of the domino process and that it is accomplished at different rates depending on the ring substituent (see Scheme 3, entries 2, 5, 8, and 12).

**Figure 2:**
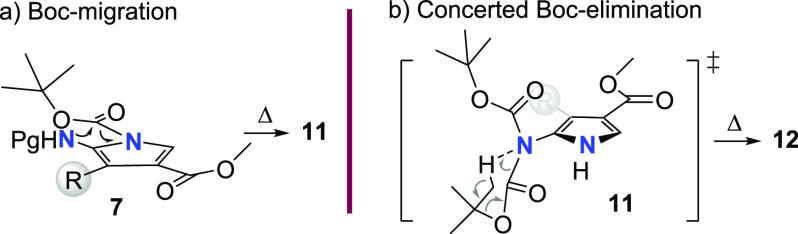
Mechanism of the migration (a) and elimination (b) of the Boc group.

In summary, we have developed a novel and facile synthetic methodology to access 2-aminopyrroles from previously unknown but easily accessible *N*-alkynyl, *N*′-vinyl hydrazides through an unprecedented 3,4-diaza Cope rearrangement and a 5-exo-dig N-cyclization reaction. Using this strategy, we built a small library of 29 different 2-aminopyrroles with diverse protection patterns. The whole domino process can be harnessed to selectively deliver a single 2-aminopyrrole with the amine nitrogen protected as its *N*-Boc and the pyrrole nitrogen free (N–H). This result highlights the symmetry-breaking power of this manifold, which transforms a linear and symmetrically protected hydrazide platform into an asymmetrically protected 2-aminopyrrole molecule.

## Supporting Information Available

Experimental procedures and spectral data for all new compounds **4**, **7**, **9**, **11**, and **12** (PDF)
