# Supplementary material for: Short and Modular Synthesis of Substituted 2-Aminopyrroles
Source: Org Lett. 2021 Apr 30;23(10):4078–82. doi: 10.1021/acs.orglett.1c01345 (PMC8483442; doi:10.1021/acs.orglett.1c01345)
Supplement: Supplementary file 1 — ol1c01345_si_001.pdf [file ol1c01345_si_001.pdf]

Electronic Supporting Information for

Short and modular synthesis of substituted 2-aminopyrroles.

Raquel Diana-Rivero,[ab] Beate Halsvik, [c†] Fernando García Tellado *[a] and David Tejedor*[a]

[a]*Instituto de Productos Naturales y Agrobiología, Consejo Superior de Investigaciones Científicas, Astrofísico Francisco Sánchez 3, 38206 La Laguna, Tenerife (Spain).*

Email: dtejedor@ipna.csic.es ; fgarcia@ipna.csic.es

[b]*Doctoral and Postgraduate School Universidad de La Laguna, Avda. Astrofísico Francisco Sánchez s/n, apdo.456, 38200 La Laguna, Santa Cruz de Tenerife (Spain).*

[c]*Department of Chemistry, University of Bergen, Allégaten 41, NO-5007 Bergen (Norway)*

Table of Contents | **Pages**

1. General Considerations	S2
2. Experimental Procedures	S2
2.1. Preparation of **9**	S2-S3
2.2. General procedure for synthesis of **4**	S3
2.3. General procedure for synthesis of **7**,**11**,**12**	S3
3. Characterization and spectral data	S3-S14
4. ^{1}H and ^{13}C NMR Spectra	S15
4.1. Compounds **9**	S15-S18
4.2. Compounds **4**	S19-S31
4.3. Compounds **7**	S32-S35
4.4. Compounds **11**	S36-S41
4.5. Compounds **12**	S42-S53

† Contributed to the work at IPNA-CSIC during a research visit.

1. General Considerations

^{1}H NMR and ^{13}C NMR spectra of $CDCl_3$ solutions were recorded either at 400 and 100 MHz or at 500 and 125 MHz (Bruker Ac 200 and AMX2-500), respectively. Mass spectra (low resolution) (EI/CI) were obtained with a Hewlett-Packard 5995 gas chromatograph/mass spectrometer. High-resolution mass spectra were recorded either with a mass spectrometer LCT Premier XE with two types of ionization sources: electrospray (ESI), an atmospheric pressure chemical ionization source (APCI), and with an orthogonal acceleration time-of-flight (oa-TOF) analyzer, or with a Micromass Autospec mass spectrometer. Analytical thin-layer chromatography plates used were E. Merck Brinkman UV-active silica gel (Kieselgel 60 F254) on aluminum. Flash column chromatography was carried out with E. Merck silica gel 60 (particle size less than 0.020 mm) using appropriate mixtures of ethyl acetate in hexane unless other solvents are specified. All reactions were performed in oven-dried glassware. All materials were obtained from commercial suppliers and used as received. Reactions were stirred under reflux conditions using a hotplate stirrer with a Heat-On™ Block System.

2. Experimental Procedures

2.1 Preparation of alkynyl hydrazides 9.

Alkynyl hydrazides **9b-g** were prepared following the reported procedure by Batey and col.[1] Alkynyl hydrazides **9a** and **9h** were prepared from the corresponding alkynyl magnesium bromide following the typical standard procedure described below. Alkynyl hydrazides **9c**, **9d**, **9f** and 9**g** have been described elsewhere.[1]

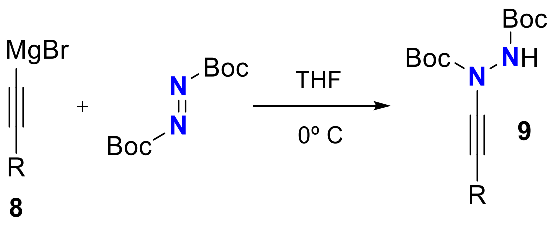

A nitrogen flushed round bottomed flask capped with a rubber septa was charged with di-t-butyl-azodicarboxylate (2.0 mmol, 1 equiv.) and cooled to 0 ºC. Then, propynyl magnesium bromide (0.5M in THF)(4.4 mL; 2.2 mmol; 1.1 equiv.) was added dropwise via cannula. The reaction mixture was stirred at 0º C for 2 hours and allowed to reach room temperature. The reaction mixture was then poured on water and extracted with ethyl acetate. The organic extract was dried ($MgSO_4$), filtered, and concentrated in vacuo. The resulting crude residue was

[1] Beveridge, R.; Batey, R. *Org. Lett.* **2012**, *14*, 540-543.

purified through silica gel (ethyl acetate/ hexanes) to provide the alkynyl hydrazides **9.**

2.2. General procedure for synthesis of alkynyl vinyl hydrazides 4.

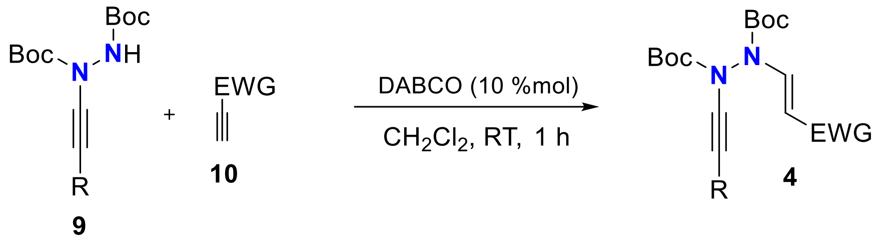

To a solution of the appropriate alkynyl hidrazide **9** (1.0 mmol, 1 equiv.) in CH_2Cl_2 (10 mL) were added DABCO (0.1 mmol, 0.1 equiv.) and the activated alkyne **10** (1.1 mmol; 1.1 equiv.). The mixture was stirred at room temperature for 1 hour. The reaction mixture was concentrated in vacuo and purified by flash chromatography (ethyl acetate / hexanes) to yield the desired alkynyl vinyl hydrazides **4**.

2.3. General procedure for the synthesis of 2-aminopyrroles 7, 11, 12.

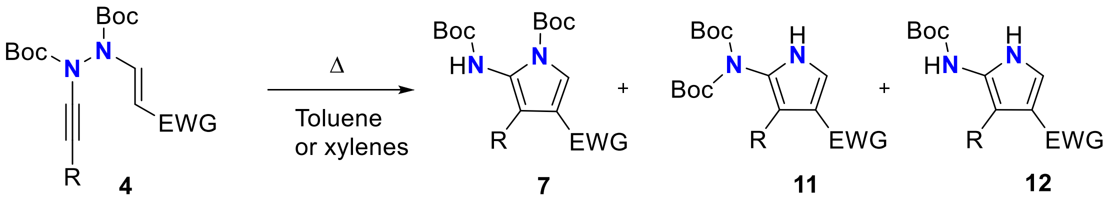

A solution of the alkynyl vinyl hydrazide **4** (0.3 mmol) in toluene or xylenes (3 mL) was stirred at reflux temperature until total consumption of the starting material. The solution was concentrated in vacuo and purified by flash chromatography. Elution with appropriate mixtures of ethyl acetate in hexanes afforded 2-aminopyrrole **7**. Further elution delivered the 2-aminopyrroles **12** and **11** consecutively.

3. Characterization and spectral data.

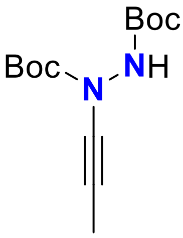

Di-tert-butyl 1-(prop-1-yn-1-yl)hydrazine-1,2-dicarboxylate (9a). Purified by silica gel chromatography (EtOAc/Hexanes: 10:90 $^{v}/_{v}$). Yellow oil (730.7 mg, 67%). 1H NMR ($CDCl_3$, 400 MHz): δ 6.63 (bs, 1H), 1.91 (s, 3H), 1.48 (s, 9H), 1.47 (s, 9H). 13C {1H} NMR ($CDCl_3$, 100 MHz): δ 154.1, 153.2, 83.8, 82.1, 72.2, 66.4, 28.1, 27.9, 3.2. HRMS (ESI-TOF) m/z: [M + Na]+ Calcd for $C_{13}H_{22}N_2O_4Na$ 293.1477; Found 293.1482.

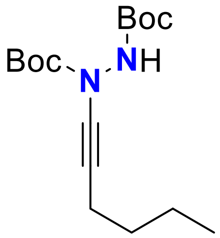

Di-tert-butyl 1-(hex-1-yn-1-yl)hydrazine-1,2-dicarboxylate (9b). Purified by silica gel chromatography (dichloromethane/Hexanes: 80:20 v/v). (347.1 mg, 37%). Pale yellow oil: 1H NMR ($CDCl_3$, 400 MHz): δ 6.84 (s, 1H), 2.23 (t, 2H, J = 6.8 Hz), 1.42 (s, 9H), 1.41 (s, 9H), 1.34 (t, 2H, J = 7.4 Hz), 0.83 (t, 3H, J = 7.1 Hz). 13C {1H} NMR ($CDCl_3$, 100 MHz): δ 154.0, 153.2, 83.4, 81.8, 73.3, 30.6, 29.5, 28.0, 27.7, 21.6, 17.9, 13.4. HRMS (ESI-TOF) m/z: [M + Na]+ Calcd for $C_{16}H_{28}N_2O_4Na$ 335.1947, Found 335.1949.

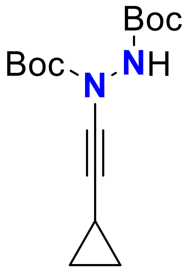

Di-tert-butyl 1-(cyclopropylethynyl)hydrazine-1,2-dicarboxylate (9e). Purified by silica gel chromatography (EtOAc/Hexanes: 20:80 v/v). (333.0 mg, 56%). 1H NMR ($CDCl_3$, 400 MHz): δ 7.03 (s, 1H), 1.33 (s, 18H), 1.25 - 1.07 (m, 1H), 0.63 (d, 2H, J = 8.0 Hz), 0.53 (s, 2H). 13C {1H} NMR ($CDCl_3$, 100 MHz): δ 152.9, 82.9, 81.2, 68.6, 27.6, 27.4, 8.0, -1.2. .HRMS (ESI-TOF) m/z: [M + Na]+ Calcd for $C_{15}H_{24}N_2O_4Na$ 319.1634; Found 319.1627.

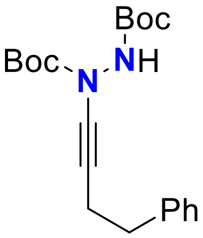

Di-tert-butyl 1-(4-phenylbut-1-yn-1-yl)hydrazine-1,2-dicarboxylate (9h). Purified by silica gel chromatography (EtOAc/Hexanes: 20:80 v/v). (324.8 mg, 27%). Yellow oil: 1H NMR ($CDCl_3$, 400 MHz): δ 7.35 – 7.16 (m, 5H), 6.91 (s, 1H), 2.86 (t, 2H, J = 7.6 Hz), 2.60 (t, 2H, J = 7.6 Hz), 1.51 (s, 9H), 1.51 (s, 9H). 13C {1H} NMR ($CDCl_3$, 100 MHz): δ 154.0, 153.1, 140.7, 128.3, 128.2, 126.1, 83.7, 82.0, 70.2, 35.1, 28.0, 27.8, 20.6. HRMS (ESI-TOF) m/z: [M + Na]+ Calcd for $C_{20}H_{28}N_2O_4Na$ 383.1947; Found 383.1944.

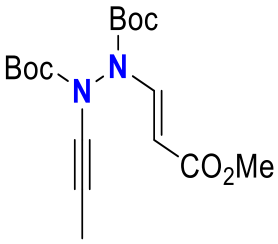

Di-tert-butyl (E)-1-(3-methoxy-3-oxoprop-1-en-1-yl)-2-(prop-1-yn-1-yl) hydrazine-1,2-dicarboxylate (4a). Purified by silica gel chromatography (EtOAc/Hexanes: 15:85 v/v). Pale yellow oil (118.8 mg, 98%): 1H NMR ($CDCl_3$, 400 MHz): δ 8.00 (d, 1H, J = 13.7 Hz), 5.36 (d, 1H, J = 13.7 Hz), 3.72 (s, 3H), 1.90 (s, 3H), 1.53 (s, 9H), 1.48 (s, 9H). 13C {1H} NMR ($CDCl_3$, 100 MHz): δ 167.3, 151.3, 149.5, 139.1, 99.1, 84.8, 84.4, 70.0, 68.1, 51.2, 28.0, 27.9, 3.0. HRMS (ESI-TOF) m/z: [M + Na]+ Calcd for $C_{17}H_{26}N_2O_6Na$ 377.1689; Found 377.1685.

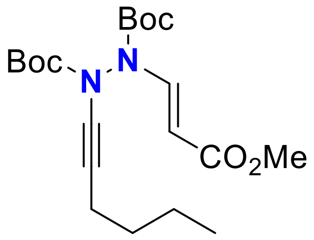

Di-tert-butyl (E)-1-(hex-1-yn-1-yl)-2-(3-methoxy-3-oxoprop-1-en-1-yl)hydrazine-1,2-dicarboxylate (4b). Purified by silica gel chromatography (EtOAc/Hexanes: 30:70 v/v). Yellow oil (417.5 mg, 95%): 1H NMR ($CDCl_3$, 500 MHz): δ 7.98 (d, 1H, J = 13.7 Hz), 5.33 (d, 1H, J = 13.7 Hz), 3.70 (s, 3H), 2.27 (t, 2H, J = 6.9 Hz), 1.51 (s, 9H), 1.46 (s, 9H), 1.44 – 1.37 (m, 2H), 0.88 (t, 3H, J = 7.2 Hz), 13C {1H} NMR ($CDCl_3$, 125 MHz): δ 167.4, 151.4, 149.6, 139.2, 99.2, 84.8, 84.4, 72.6, 71.3, 51.3, 30.9, 28.1, 28.0, 21.9, 18.2, 13.5. HRMS (ESI-TOF) m/z: [M + Na]+ Calcd for $C_{20}H_{32}N_2O_6Na$ 419.2158; Found 419.2160.

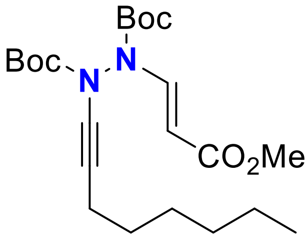

Di-tert-butyl (E)-1-(3-methoxy-3-oxoprop-1-en-1-yl)-2-(oct-1-yn-1-yl)hydrazine-1,2-dicarboxylate (4c). Purified by silica gel chromatography (EtOAc/Hexanes: 10:90 v/v). Yellow oil (473.4 mg, 94%): 1H NMR ($CDCl_3$, 500 MHz): δ 8.07 (d, 1H, J = 13.8 Hz), 7.99 (d, 1H, J = 13.8 Hz). 5.34 (d, 1H, J = 13.8 Hz), 3.70 (s, 3H), 2.26 (t, 2H, J = 7.0 Hz), 1.52 (s, 9H), 1.51 – 1.48 (m, 2H), 1.47 (s, 9H), 1.41 – 1.34 (m, 2H), 1.32 – 1.22 (m, 4H), 0.88 (t, 3H, J = 6.9 Hz). 13C {1H} NMR ($CDCl_3$, 125 MHz): δ 167.3, 151.2, 149.5, 139.0, 99.0, 84.3, 72.5, 71.2, 51.1, 31.3, 28.7, 28.4, 28.0, 27.9, 22.4, 18.4, 13.8. HRMS (ESI-TOF) m/z: [M + Na]+ Calcd for $C_{22}H_{36}N_2O_6Na$ 447.2471; Found 447.2471.

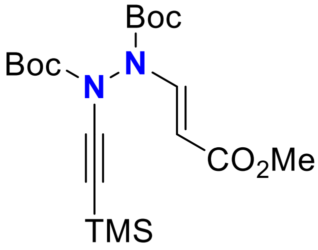

Di-tert-butyl (E)-1-(3-methoxy-3-oxoprop-1-en-1-yl)-2-((trimethylsilyl)ethynyl)hydrazine-1,2-dicarboxylate (4d). Purified by silica gel chromatography (EtOAc/Hexanes: 20:80 v/v). Pale yellow oil (114.4 mg, 92%): 1H NMR ($CDCl_3$, 500 MHz): δ 8.01 (d, 1H, J = 13.7 Hz), 5.37 (d, 1H, J = 13.9 Hz), 3.73 (s, 3H), 1.54 (s, 9H), 1.50 (s, 9H), 0.18 (s, 9H). 13C {1H} NMR ($CDCl_3$, 125 MHz): δ 167.2, 150.6, 138.9, 99.3, 92.1, 84.9, 51.2, 28.0, 27.9, -0.1. HRMS (ESI-TOF) m/z: [M + Na]+ Calcd for $C_{19}H_{32}N_2O_6SiNa$ 435.1927; Found 435.1929.

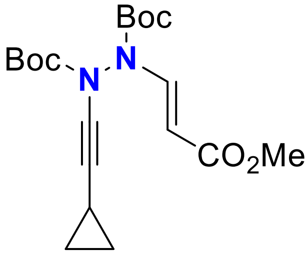

Di-tert-butyl (E)-1-(cyclopropylethynyl)-2-(3-methoxy-3-oxoprop-1-en-1-yl)hydrazine-1,2-dicarboxylate (4e). Purified by silica gel chromatography (EtOAc/Hexanes: 15:85 v/v). (134.3 mg, 88%). 1H NMR ($CDCl_3$, 400 MHz): δ 7.90 (d, 1H, J = 13.7 Hz), 5.23 (d, 1H, J = 15.7 Hz), 3.64 (s, 3H), 1.44 (s, 9H), 1.35 (s, 9H), 1.23 (d, 1H, J = 6.8 Hz), 0.71 (d, 2H, J = 7.9 Hz), 0.60 (s, 2H). 13C {1H} NMR ($CDCl_3$, 100 MHz): δ =

167.3, 151.4, 149.5, 139.1, 99.1, 84.4, 66.7, 51.1, 29.7, 28.0, 27.9, 8.7, -0.8. HRMS (ESI-TOF) m/z: [M + Na]+ Calcd for $C_{19}H_{28}N_2O_6Na$ 403.1845; Found 403.1847.

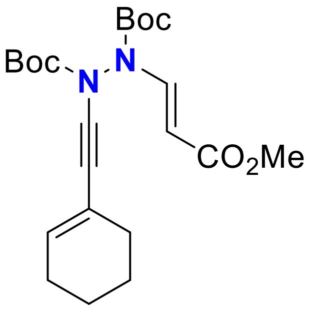

Di-tert-butyl (E)-1-(cyclohex-1-en-1-ylethynyl)-2-(3-methoxy-3-oxoprop-1-en-1-yl) hydrazine-1,2-dicarboxylate (4f). Purified by silica gel chromatography (EtOAc/Hexanes: 15:85 $^{v}/_{v}$). Yellowish oil (192.9 mg, 80%): 1H NMR ($CDCl_3$, 500 MHz): δ 8.00 (d, 1H, J =13.7 Hz), 6.03 (s, 1H), 5.35 (d, 1H, J = 13.7 Hz), 3.70 (s, 3H), 2.12 – 2.02 (m, 4H), 1.65 – 1.52 (m, 4H), 1.51 (s, 9H), 1.47 (s, 9H). 13C {1H} NMR ($CDCl_3$, 125 MHz): δ 167.2, 150.5, 149.4, 138.6, 135.6, 134.0, 119.4, 98.8, 84.5, 51.2, 29.1, 27.8, 27.7, 25.5, 22.1, 21.3. HRMS (ESI-TOF) m/z: [M + Na]+ Calcd for $C_{22}H_{32}N_2O_6Na$ 443.2158; Found 443.2154.

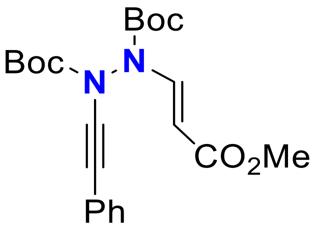

Di-tert-butyl (E)-1-(3-methoxy-3-oxoprop-1-en-1-yl)-2-(phenylethynyl)hydrazine-1,2-dicarboxylate (4g). Purified by silica gel chromatography (EtOAc/Hexanes: 20:80 $^{v}/_{v}$). Yellow oil (184.7 mg, 89%): 1H NMR ($CDCl_3$, 500 MHz): δ 7.40-7.36 (m, 2H), 7.29-7.26 (m, 3H), 5.46 (d, 1H, J = 13.8 Hz), 3.73 (s, 3H), 1.56 (s, 9H), 1.53 (s, 9H). 13C {1H} NMR ($CDCl_3$, 125 MHz): δ 167.2, 150.7, 149.4, 139.1, 131.5, 128.2, 128.1, 122.5, 99.4, 85.0, 80.0, 72.8, 51.2, 28.1, 27.9. HRMS (ESI-TOF) m/z: [M + Na]+ Calcd for $C_{22}H_{28}N_2O_6Na$ 439.1845; Found 439.1841.

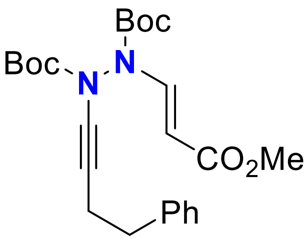

Di-tert-butyl (E)-1-(3-methoxy-3-oxoprop-1-en-1-yl)-2-(4-phenylbut-1-yn-1-yl)hydrazine-1,2-dicarboxylate (4h). Purified by silica gel chromatography (EtOAc/Hexanes: 15:85 $^{v}/_{v}$). Yellow oil (132 mg, 94%): 1H NMR ($CDCl_3$, 500 MHz): δ 8.00 (d, 1H, J = 12.9 Hz), 5.34 (d, 1H, J = 13.8 Hz), 7.27 – 7.17 (m, 5H), 2.82 (t, 2H, J = 7.0 Hz), 3.74 (s, 3H), 2.59 (t, 2H, J = 7.3 Hz), 1.52 (s, 9H), 1.48 (s, 9H). 13C {1H} NMR ($CDCl_3$, 125 MHz): δ 167.2, 151.0, 149.3, 140.4, 138.9, 128.4, 128.2, 126.2, 99.0, 84.7, 84.4, 71.8, 51.1, 27.9, 27.8, 20.4. HRMS (ESI-TOF) m/z: [M + Na]+ Calcd for $C_{24}H_{32}N_2O_6Na$ 467.2158; Found 467.2163.

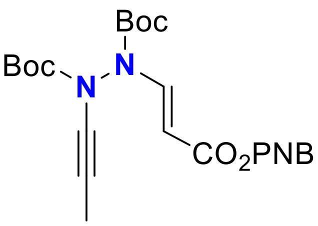

Di-tert-butyl (E)-1-(3-((4-nitrobenzyl)oxy)-3-oxoprop-1-en-1-yl)-2-(prop-1-yn-1-yl)hydrazine-1,2-dicarboxylate (4i) (133.2 mg, 85%). Purified by silica gel chromatography (EtOAc/Hexanes: 30:70 $^{v}/_{v}$). Yellow oil: 1H NMR ($CDCl_3$, 500 MHz): δ 1.47 (s, 9H), 1.52 (s, 9H), 1.91 (s, 3H), 5.25 (s, 2H), 5.40 (d, 1H, J = 13.6 Hz), 7.51 (d, 2H, J = 8.3 Hz,), 8.08 (bs, 1H), 8.19 (d, 2H, J = 8.3 Hz). 13C {1H} NMR ($CDCl_3$, 125 MHz): δ 3.1, 27.8, 27.9, 64.5, 68.2, 69.7, 84.6, 98.1, 123.7, 128.3, 139.9, 143.6, 147.8, 149.3, 151.2, 166.4. HRMS (ESI-TOF) m/z: [M + Na]+ Calcd for $C_{23}H_{29}N_3O_8Na$ 498.1852; Found 498.1847.

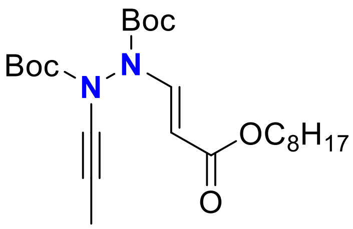

Di-tert-butyl (E)-1-(3-(octyloxy)-3-oxoprop-1-en-1-yl)-2-(prop-1-yn-1-yl)hydrazine-1,2-dicarboxylate (4j). Purified by silica gel chromatography (EtOAc/Hexanes: 5:95 $^{v}/_{v}$). Pale yellow oil (409.4 mg, 90%): 1H NMR ($CDCl_3$, 500 MHz): δ 7.98 (bs, 1H), 5.35 (d, 1H, J = 13.7 Hz), 4.11 (t, 2H, J = 6.7 Hz), 1.91 (s, 3H), 1.63-1.65 (m, 2H), 1.52 (s, 9H), 1.47 (bs, 9H), 1.27-1.36 (m, 10H), 0.87 (t, 3H, J = 6.7 Hz). 13C {1H} NMR ($CDCl_3$, 125 MHz): δ 167.0, 151.3, 149.5, 138.8, 99.5, 84.7, 84.4, 69.9, 68.0, 64.4, 31.8, 29.2, 29.1, 28.8, 28.0 (3C), 27.9 (3C), 26.0, 22.6, 14.0, 3.1. HRMS (ESI-TOF) m/z: [M + Na]+ Calcd for $C_{24}H_{40}N_2O_6Na$ 475.2784; Found 475.2790.

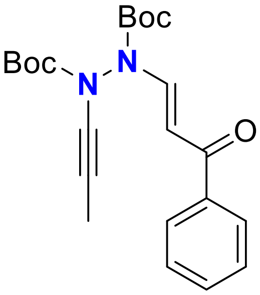

Di-tert-butyl (E)-1-(3-oxo-3-phenylprop-1-en-1-yl)-2-(prop-1-yn-1-yl)hydrazine-1,2-dicarboxylate (4k) (113.0 mg, 91%). Purified by silica gel chromatography (EtOAc/Hexanes: 10:90 $^{v}/_{v}$). Yellow oil: 1H NMR ($CDCl_3$, 500 MHz): δ 1.48 (s, 9H), 1.55 (s, 9H), 1.93 (s, 3H), 6.43 (s, 1H), 7.42-7.45 (m, 2H), 7.50-7.53 (m, 1H), 7.90 (d, 2H, J = 7.7 Hz), 8.16 (d, 1H, J = 13.5 Hz). 13C {1H} NMR ($CDCl_3$, 125 MHz): δ 3.2, 27.9, 28.0, 68.2, 69.9, 84.6, 103.7, 128.0, 128.2, 128.4, 132.3, 138.6, 139.7, 149.4, 151.5. 189.6. HRMS (ESI-TOF) m/z: [M + Na]+ Calcd for $C_{22}H_{28}N_2O_5Na$ 423.1896; Found 423.1898.

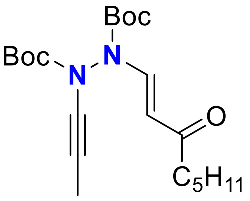

Di-tert-butyl (E)-1-(3-oxooct-1-en-1-yl)-2-(prop-1-yn-1-yl)hydrazine-1,2-dicarboxylate (4l) (96.9 mg, 66%). Purified by silica gel chromatography (EtOAc/Hexanes: 15:85 $^{v}/_{v}$). Yellow oil: 1H NMR ($CDCl_3$, 500 MHz): δ 0.86 (t, 3H, J = 6.9 Hz), 1.27-1.30 (m, 4H), 1.45 (s, 9H), 1.51 (s, 9H), 1.59 – 1.62 (m, 2H), 1.90 (s, 3H), 2.47 (t, 2H, J = 7.5 Hz), 5.67 (d, 1H, J = 13.1 Hz), 7.92 (d, 1H, J = 12.3 Hz). 13C {1H} NMR ($CDCl_3$, 125 MHz): δ 3.1, 13.8, 22.4, 24.2, 27.8, 27.9, 31.4, 41.7, 68.0, 69.8, 84.4, 108.2, 137.5, 149.5, 151.4,

199.2. HRMS (ESI-TOF) m/z: [M + Na]+ Calcd for $C_{21}H_{34}N_2O_5Na$ 417.2365; Found 417.2361.

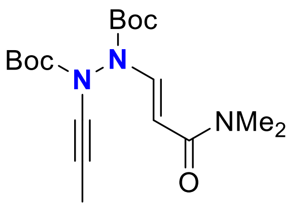

Di-tert-butyl (E)-1-(3-(dimethylamino)-3-oxoprop-1-en-1-yl)-2-(prop-1-yn-1-yl)hydrazine-1,2-dicarboxylate (4m). Purified by silica gel chromatography (EtOAc/Hexanes: 60:40 $^{v}/_{v}$). Pale yellow oil (758 mg, 86%): 1H NMR ($CDCl_3$, 500 MHz): δ 7.92 (d, 1H, J = 13.2 Hz), 5.70 (d, 1H, J = 13.2 Hz), 2.99 (s, 6H), 1.90 (s, 3H), 1.50 (s, 9H), 1.44 (s, 9H). 13C {1H} NMR ($CDCl_3$, 125 MHz): δ 166.5, 151.8, 149.5, 137.3, 98.5, 84.3, 84.2, 70.1, 67.8, 37.2, 35.7, 27.8, 27.7, 3.1. HRMS (ESI-TOF) m/z: [M + Na]+ Calcd for $C_{18}H_{29}N_3O_5Na$ 390.2005; Found 390.2005.

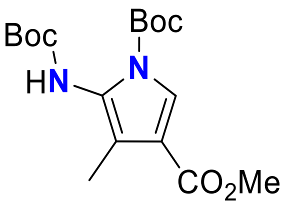

1-(tert-Butyl) 3-methyl 5-((tert-butoxycarbonyl)amino)-4-methyl-1H-pyrrole-1,3-dicarboxylate (7a). Purified by silica gel chromatography (EtOAc/Hexanes: 30:70 $^{v}/_{v}$). Pale yellow solid (55.0 mg, 17%): 1H NMR ($CDCl_3$, 400 MHz): δ 7.65 (s, 1H), 6.39 (bs, 1H), 3.79 (s, 3H), 2.12 (s, 3H), 1.57 (s, 9H), 1.45 (s, 9H). 13C {1H} NMR ($CDCl_3$, 100 MHz): δ 164.7, 153.7, 148.1, 124.2, 123.9, 119.1, 115.7, 85.0, 80.7, 51.1, 28.2, 27.9, 10.1. HRMS (EI) m/z: [M]+ Calcd for $C_{17}H_{26}N_2O_6$ 354.1791; Found 354.1788.

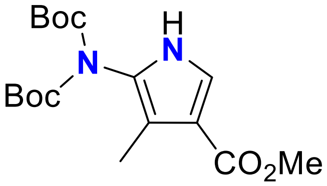

Methyl 5-(bis(tert-butoxycarbonyl)amino)-4-methyl-1H-pyrrole-3-carboxylate (11a). Purified by silica gel chromatography (EtOAc/Hexanes: 30:70 $^{v}/_{v}$). Brown solid (173.8 mg, 53%): 1H NMR ($CDCl_3$, 500 MHz): δ 8.38 (bs, 1H), 7.24 (d, 1H, J = 3.0 Hz), 3.77 (s, 3H), 2.10 (s, 3H), 1.40 (s, 18H). 13C {1H} NMR ($CDCl_3$, 125 MHz): δ 165.6, 151.2, 123.6, 121.5, 115.9, 113.9, 83.2, 50.7, 27.8, 9.3. HRMS (EI) m/z: [M]+ Calcd for $C_{17}H_{26}N_2O_6$ 354.1791; Found 354.1788.

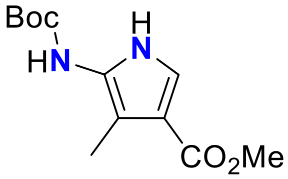

Methyl 5-((tert-butoxycarbonyl)amino)-4-methyl-1H-pyrrole-3-carboxylate (12a). Purified by silica gel chromatography (EtOAc/Hexanes: 30:70 $^{v}/_{v}$). Red solid (67.7 mg, 82%): 1H NMR ($CDCl_3$, 400 MHz): δ 9.66 (bs, 1H), 7.11 (d, 1H, J = 3.0 Hz), 6.46 (bs, 1H), 3.75 (s, 3H), 2.12 (s, 3H), 1.48 (s, 9H). 13C {1H} NMR ($CDCl_3$, 100 MHz): δ 165.8, 153.6, 125.0, 118.4, 113.4, 103.9, 81.4, 50.6, 28.2, 8.9. HRMS HRMS (EI) m/z: [M]+ Calcd for $C_{12}H_{18}N_2O_4$ 254.1267; Found 254.1265.

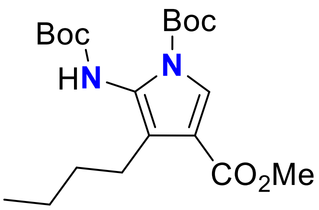

1-(tert-butyl) 3-methyl 5-((tertbutoxycarbonyl)amino)-4-butyl-1H-pyrrole-1,3-dicarboxylate (7b). Purified by silica gel chromatography (EtOAc/Hexanes: 20:80 v/v). Yellow oil (27.8 mg, 28%): 1H NMR ($CDCl_3$, 400 MHz): δ 7.69 (s, 1H), 6.09 (s, 1H), 3.79 (s, 3H), 2.59 (t, 2H, J = 7.8 Hz), 1.57 (s, 9H), 1.45 (s, 9H), 1.37 – 1.27 (m, 2H), 1.24 (s, 2H), 0.89 (t, 3H, J = 7.3 Hz). 13C {1H} NMR ($CDCl_3$, 100 MHz): δ 164.5, 124.7, 84.8, 80.7, 51.0, 31.9, 28.2, 27.9, 23.8, 22.6, 13.9. HRMS (ESI-TOF) m/z: [M + Na]+ Calcd for $C_{20}H_{32}N_2O_6Na$ 419.2158; Found 419.2157.

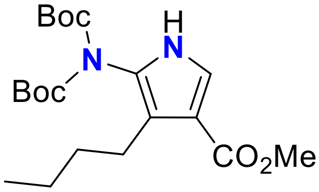

Methyl 5-(bis(tert-butoxycarbonyl)amino)-4-butyl-1H-pyrrole-3-carboxylate (11b). Purified by silica gel chromatography (EtOAc/Hexanes: 20:80 v/v). Yellow oil (34.9 mg, 36%): 1H NMR ($CDCl_3$, 400 MHz): δ 8.08 (s, 1H), 7.27 (d, 1H, J = 3.3 Hz), 3.78 (s, 3H), 2.54 (t, 2H, J = 7.7 Hz), 1.41 (s, 18H), 1.35-1.29 (m, 4H), 0.88 (t, 3H, J = 7.2 Hz). 13C {1H} NMR ($CDCl_3$, 100 MHz): δ 165.3, 151.2, 123.3, 121.8, 121.0, 113.6, 83.2, 50.7, 32.0, 27.8, 23.8, 22.8, 13.9. HRMS (ESI-TOF) m/z: [M + Na]+ Calcd for $C_{20}H_{32}N_2O_6Na$ 419.2158; Found 419.2160.

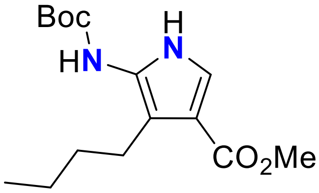

Methyl 5-((tert-butoxycarbonyl)amino)-4-butyl-1H-pyrrole-3-carboxylate (12b). Purified by silica gel chromatography (EtOAc/Hexanes: 20:80 v/v). Red oil (96.3 mg, 75%).: 1H NMR ($CDCl_3$, 400 MHz): δ 9.71 (s, 1H), 7.10 (d, 1H, J = 3.1 Hz), 6.42 (s, 1H), 3.75 (s, 3H), 2.56 (t, 2H, J = 7.4 *Hz*), 1.49 (s, 9H), 1.37 – 1.27 (m, 4H), 0.90 (t, 3H, J = 7.3 Hz). 13C {1H} NMR ($CDCl_3$, 100 MHz): δ 165.5, 153.6, 124.8, 118.6, 112.8, 109.2, 81.4, 50.6, 32.9, 28.2, 23.4, 22.6, 13.9. HRMS (ESI-TOF) m/z: [M + Na]+ Calcd for $C_{15}H_{24}N_2O_4Na$ 319.1634; Found 319.1637.

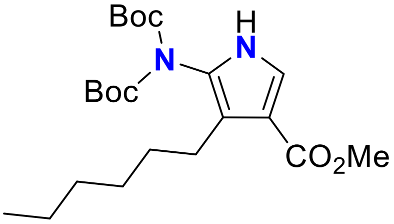

Methyl 5-(bis(tert-butoxycarbonyl)amino)-4-hexyl-1H-pyrrole-3-carboxylate (11c). Purified by silica gel chromatography (EtOAc/Hexanes: 20:80 v/v). Yellow oil (32.0 mg, 34%): 1H NMR ($CDCl_3$, 500 MHz): δ 8.31 (s, 1H), 7.27 (s, 1H), 3.78 (s, 3H), 2.57–2.49 (m, 2H), 1.51 (s, 4H), 1.41 (s, 18H), 1.31 – 1.25 (m, 4H), 0.86 (t, 3H, J = 6.9 Hz). 13C {1H} NMR ($CDCl_3$, 125 MHz): δ 165.3, 151.3, 123.2, 121.8, 121.0, 113.5, 83.2, 50.6, 31.8, 29.9, 29.5, 27.8, 24.2, 22.6,

14.1. HRMS (ESI-TOF) m/z: [M + Na]+ Calcd for $C_{22}H_{36}N_2O_6Na$ 447.2471; Found 447.2471.

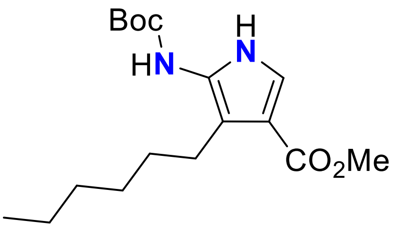

Methyl 5-((tert-butoxycarbonyl)amino)-4-hexyl-1H-pyrrole-3-carboxylate (12c). Purified by silica gel chromatography (EtOAc/Hexanes: 20:80 $^v/_v$). Brown solid (83.2 mg, 71%): 1H NMR ($CDCl_3$, 400 MHz): δ 9.71 (s, 1H). 7.11 (d, 1H, J = 3.1 Hz), 6.40 (s, 1H), 3.75 (s, 3H), 2.55 (t, 2H, J = 7.6 Hz,), 1.49 (s, 9H), 1.28 (s, 8H), 0.86 (t, 3H, J = 6.5 Hz). 13C {1H} NMR ($CDCl_3$, 100 MHz): δ 165.5, 153.5, 124.9, 118.6, 112.8, 109.0, 81.5, 50.6, 31.8, 30.7, 29.2, 28.2, 23.6, 22.7, 14.1. HRMS (ESI-TOF) m/z: [M + Na]+ Calcd for $C_{17}H_{28}N_2O_4Na$ 347.1947; Found 347.1950.

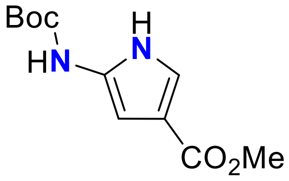

Methyl 5-((tert-butoxycarbonyl)amino)-1H-pyrrole-3-carboxylate (12d). Purified by silica gel chromatography (EtOAc/Hexanes: 20:80 $^v/_v$). Yellow oil (15.8 mg, 18%): 1H NMR ($CDCl_3$, 500 MHz): δ 10.02 (bs, 1H), 7.11 (s, 1H), 6.81 (s, 1H), 5.88 (s, 1H), 3.78 (s, 3H), 1.49 (s, 9H). 13C {1H} NMR ($CDCl_3$, 125 MHz): δ 165.4, 153.2, 128.9, 117.9, 114.6, 92.2, 81.6, 51.1, 28.2. HRMS (ESI-TOF) m/z: [M + Na]+ Calcd for $C_{11}H_{16}N_2O_4Na$ 263.1008; Found 263.1010.

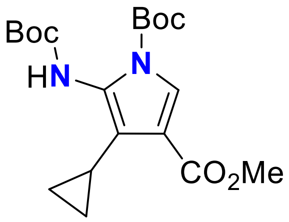

1-(tert-butyl) 3-methyl 5-((tert-butoxycarbonyl)amino)-4-cyclopropyl-1H-pyrrole-1,3-dicarboxylate (7e). Purified by silica gel chromatography (EtOAc/Hexanes: 20:80 $^v/_v$). Yellow oil (8.7 mg, 7%): 1H NMR ($CDCl_3$, 400 MHz): δ 7.60 (s, 1H), 6.55 (bs, 1H), 3.78 (s, 3H), 1.88-1.82 (m, 1H), 1.55 (s, 9H), 1.45 (s, 9H), 0.77 (d, 2H, J = 8.3 Hz), 0.58 (s, 2H). 13C {1H} NMR ($CDCl_3$, 100 MHz): δ 164.3, 153.5, 148.1, 124.7, 123.7, 122.8, 116.4, 85.0, 80.6, 51.1, 28.1, 27.8, 6.9, 6.1, HRMS (ESI-TOF) m/z: [M + Na]+ Calcd for $C_{19}H_{28}N_2O_6Na$ 403.1845; Found 403.1848.

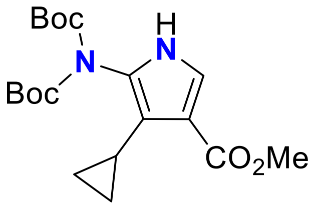

Methyl 5-(bis(tert-butoxycarbonyl)amino)-4-cyclopropyl-1H-pyrrole-3-carboxylate (11e). Purified by silica gel chromatography (EtOAc/Hexanes: 20:80 $^v/_v$). Pale yellow solid (34.4 mg, 28%): 1H NMR ($CDCl_3$, 400 MHz): δ 8.28 (bs, 1H), 7.23 (d, 1H, J = 3.3 Hz, 1H), 3.78 (s, 3H), 1.79-1.85 (m, 1H), 1.41 (s, 18H), 0.79– 0.75 (m, 2H), 0.56 – 0.53 (m, 2H). 13C {1H} NMR ($CDCl_3$, 100 MHz): δ 165.2, 151.2, 123.6, 121.6, 120.1,

114.9, 83.2, 50.7, 27.8, 6.3, 5.2. HRMS (ESI-TOF) m/z: [M + Na]+ Calcd for $C_{19}H_{28}N_2O_6Na$ 403.1845; Found 403.1845.

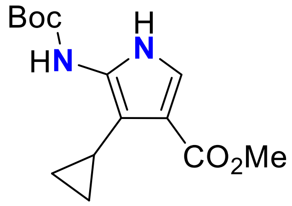

Methyl 5-((tert-butoxycarbonyl)amino)-4-cyclopropyl-1H-pyrrole-3-carboxylate (12e). Purified by silica gel chromatography (EtOAc/Hexanes: 20:80 $^{v}/_{v}$). Yellow oil (84.8 mg, 85%): 1H NMR ($CDCl_3$, 400 MHz): δ 9.85 (s, 1H), 7.05 (s, 1H), 6.77 (s, 1H), 3.77 (s, 3H), 1.63-1.58 (m, 1H), 1.50 (s, 9H), 0.89 – 0.87 (m, 2H), 0.46 (d, 2H, J = 4.0 Hz). 13C {1H} NMR ($CDCl_3$, 100 MHz): δ 165.3, 153.1, 127.3, 117.7, 114.2, 106.6, 81.6, 50.7, 28.3, 6.0, 4.6. HRMS (ESI-TOF) m/z: [M + Na]+ Calcd for $C_{14}H_{20}N_2O_4Na$ 303.1321; Found 303.1329.

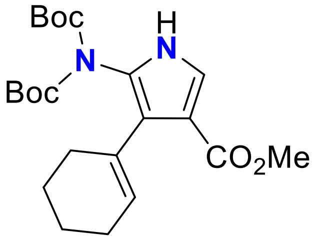

Methyl 5-(bis(tert-butoxycarbonyl)amino)-4-(cyclohex-1-en-1-yl)-1H-pyrrole-3-carboxylate (11f). Purified by silica gel chromatography (EtOAc/Hexanes: 15:85 $^{v}/_{v}$). Brown-red oil (52.0 mg, 50%): 1H NMR ($CDCl_3$, 400 MHz): δ 8.21 (s, 1H), 7.26 (d, 1H , J = 3.3 Hz), 5.56 (s, 1H), 3.76 (s, 3H), 2.27 – 2.16 (m, 2H), 2.15 – 2.06 (m, 2H), 1.75 – 1.63 (m, 2H), 1.63 (dd, 2H, J = 5.9 and 2.1 Hz), 1.40 (s, 18H).). 13C {1H} NMR ($CDCl_3$, 100 MHz): δ 165.0, 151.3, 130.9, 125.9, 123.2, 122.7, 122.0, 113.0, 82.9, 50.8, 28.8, 27.8, 25.4, 23.0, 22.1 HRMS (ESI-TOF) m/z: [M + Na]+ Calcd for $C_{22}H_{32}N_2O_6Na$ 443.2158; Found 443.2153.

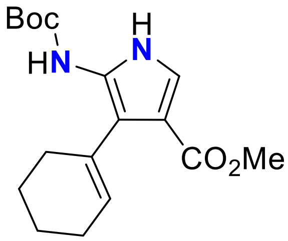

Methyl 5-((tert-butoxycarbonyl)amino)-4-(cyclohex-1-en-1-yl)-1H-pyrrole-3-carboxylate (12f). Purified by silica gel chromatography (EtOAc/Hexanes: 15:85 $^{v}/_{v}$). (33.4 mg, 42%): 1H NMR ($CDCl_3$, 400 MHz): δ 10.06 (s, 1H), 7.09 (s, 1H), 6.75 (s, 1H), 5.59 (s, 1H), 3.75 (s, 3H), 2.20 (s, 4H), 1.72 (d, 4H, J = 15.5 Hz), 1.51 (s, 9H), 13C {1H} NMR ($CDCl_3$, 100 MHz): δ 165.1. 153.2, 132.3, 126.2, 125.5, 117.9, 112.7, 109.5, 81.6, 50.8, 29.7, 28.2, 25.6, 23.0, 22.2. HRMS (ESI-TOF) m/z: [M + Na]+ Calcd for $C_{17}H_{24}N_2O_4Na$ 343.1634; Found 343.1636.

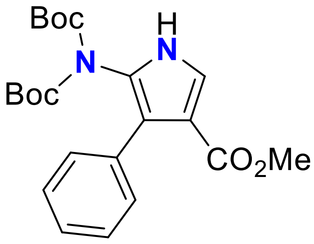

Methyl 5-(bis(tert-butoxycarbonyl)amino)-4-phenyl-1H-pyrrole-3-carboxylate (11g). Purified by silica gel chromatography (EtOAc/Hexanes: 20:80 $^{v}/_{v}$). Brownish oil (31.3 mg, 19%): 1H NMR ($CDCl_3$, 500 MHz): δ 8.92 (bs, 1H), 7.36 (d, 1H, J = 3.3 Hz), 7.34-7.28 (m, 4H), 7.26-7.23 (m, 1H), 3.69 (s, 3H), 1.30 (s, 18H). 13C {1H} NMR

($CDCl_3$, 125 MHz): δ 165.0, 150.8, 132.9, 129.5, 127.7, 126.8, 123.9, 122.5, 120.5, 113.0, 83.2, 50.8, 27.7. HRMS (ESI-TOF) m/z: [M + Na]+ Calcd for $C_{22}H_{28}N_2O_6Na$ 439.1845; Found 439.1852.

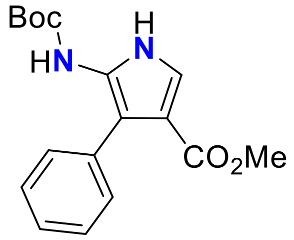

Methyl 5-((tert-butoxycarbonyl)amino)-4-phenyl-1H-pyrrole-3-carboxylate (12g). Purified by silica gel chromatography (EtOAc/Hexanes: 40:60 v/v). Amorphous pale brown solid (69.2 mg, 57%): 1H NMR ($CDCl_3$, 500 MHz): δ 10.22 (bs, 1H), 7.43-7.40 (m, 2H), 7.35-7.29 (m, 3H), 7.22 (d, 1H, J = 3.1 Hz), 6.72 (bs, 1H), 3.67 (s, 3H), 1.47 (s, 9H). 13C {1H} NMR ($CDCl_3$, 125 MHz): δ 165.0, 153.2, 133.0, 130.1, 128.3, 126.7, 126.5, 118.5, 112.7, 107.3, 81.8, 50.7, 28.2. HRMS (EI) m/z: [M]+ Calcd for $C_{17}H_{20}N_2O_4$ 316.1423; Found 316.1434.

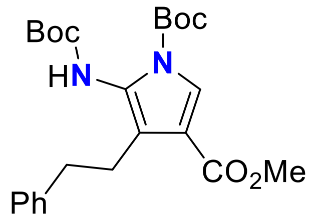

1-(tert-butyl) 3-methyl 5-((tert-butoxycarbonyl)amino)-4-phenethyl-1H-pyrrole-1,3-dicarboxylate (7h). Purified by silica gel chromatography (EtOAc/Hexanes: 20:80 v/v). Yellow oil (15.4 mg, 12%): 1H NMR ($CDCl_3$, 400 MHz): δ 7.71 (s, 1H), 7.27 – 7.15 (m, 5H), 5.82 (bs, 1H), 3.82 (s, 3H), 2.86 (d, 4H, J = 7.0 Hz), 1.56 (s, 9H), 1.45 (s, 9H). 13C {1H} NMR ($CDCl_3$, 100 MHz): δ 164.4, 154.1, 147.9, 142.5, 128.6, 128.2, 125.7, 124.7, 124.2, 123.5, 114.8, 84.9, 80.7, 51.2, 36.0, 28.2, 27.9, 26.8. HRMS (ESI-TOF) m/z: [M + Na]+ Calcd for $C_{24}H_{32}N_2O_6Na$ 467.2158; Found 467.2156.

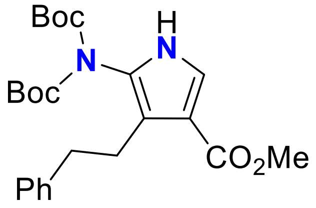

Methyl 5-(bis(tert-butoxycarbonyl)amino)-4-phenethyl-1H-pyrrole-3-carboxylate (11h). Purified by silica gel chromatography (EtOAc/Hexanes: 20:80 v/v). Pale yellow solid (27 mg, 21%): 1H NMR ($CDCl_3$, 400 MHz): δ 8.37 (s, 1H), 7.30 – 7.27 (m, 5H), 7.20 – 7.15 (m, 1H), 3.81 (s, 3H), 2.80 (d, 4H, J = 1.5 Hz), 1.42 (s, 18H). 13C {1H} NMR ($CDCl_3$, 100 MHz): δ 165.2, 151.3, 142.6, 128.4, 128.3, 125.7, 123.3, 122.0, 120.3, 113.6, 83.4, 50.8, 50.8, 36.4, 28.2, 27.9, 26.8. HRMS (ESI-TOF) m/z: [M + Na]+ Calcd for $C_{24}H_{32}N_2O_6Na$ 467.2158; Found 467.2155.

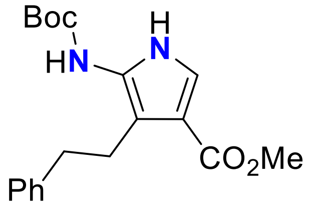

Methyl 5-((tert-butoxycarbonyl)amino)-4-phenethyl-1H-pyrrole-3-carboxylate (12h). Purified by silica gel chromatography (EtOAc/Hexanes: 20:80 v/v). (59.4 mg, 63%): 1H NMR ($CDCl_3$, 400 MHz): δ 9.60 (bs, 1H), 7.28 – 7.11 (m, 5H), 5.44 (s, 1H), 3.81 (s, 3H), 2.89 (t, 2H, J = 6.1

Hz), 2.83 (t, 2H, J = 6.1 Hz), 1.43 (s, 9H). 13C {1H} NMR ($CDCl_3$, 100 MHz): δ 165.53. 153.4, 142.6, 128.7, 128.3, 125.9, 118.6, 112.6, 107.6, 81.0, 50.7, 36.8, 28.1, 26.4. HRMS (ESI-TOF) m/z: [M + Na]+ Calcd for $C_{19}H_{24}N_2O_4Na$ 367.1634; Found 367.1634.

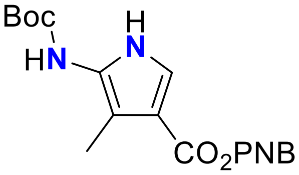

4-nitrobenzyl 5-((tert-butoxycarbonyl)amino)-4-methyl-1H-pyrrole-3-carboxylate (12i) (61.2 mg, 59%). Purified by silica gel chromatography ($MeOH/CH_2Cl_2$: 0.5:99.5 v/v). Yellow solid: 1H NMR ($CDCl_3$, 400 MHz): δ 1.50 (s, 9H), 2.14 (s, 3H), 5.33 (s, 2H), 6.41 (s, 1H), 7.20 (d, 1H, J = 3.1 Hz), 7.54 (d, 2H, J = 8.7 Hz), 8.20 (d, 2H, J = 8.7 Hz), 9.74 (s, 1H). 13C {1H} NMR ($CDCl_3$, 100 MHz): δ 8.9, 28.2, 63.7, 81.6, 103.6, 112.7, 123.7, 125.4, 128.0, 144.4, 147.5, 153.5, 164.6. HRMS (ESI-TOF) m/z: [M + Na]+ Calcd for $C_{18}H_{21}N_3O_6Na$ 398.1328; Found 398.1325.

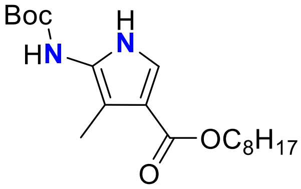

Octyl 5-((tert-butoxycarbonyl)amino)-4-methyl-1H-pyrrole-3-carboxylate (12j). Purified by silica gel chromatography (EtOAc/Hexanes: 15:85 v/v). Brownish oil (208.8 mg, 78%): 1H NMR ($CDCl_3$, 400 MHz): δ 9.63 (bs, 1H), 7.12 (d, 1H, J = 3.0 Hz), 6.43 (bs, 1H), 4.17 (t, 2H, J = 6.7 Hz), 2.13 (s, 3H), 1.64-1.70 (m, 2H), 1.49 (s, 9H), 1.26-1.40 (m, 10H), 0.86 (t, 3H, J = 6.7 Hz). 13C {1H} NMR ($CDCl_3$, 100 MHz): δ 165.5, 153.5, 124.9, 118.4, 113.8, 103.7, 81.4, 63.6, 31.8, 29.22, 29.17, 28.8, 28.2 (3C), 26.1, 22.6, 14.0, 9.0. HRMS (ESI-TOF) m/z: [M + Na]+ Calcd for $C_{19}H_{32}N_2O_4Na$ 375.2260, Found 375.2261.

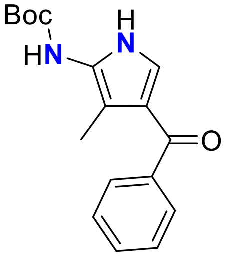

tert-Butyl (4-benzoyl-3-methyl-1H-pyrrol-2-yl)carbamate (12k). (21.6 mg, 26%). Purified by silica gel chromatography (EtOAc/Hexanes: 20:80 v/v). Orange oil: 1H NMR ($CDCl_3$, 500 MHz): δ 1.50 (s, 9H), 2.23 (s, 3H), 6.54 (s, 1H), 6.82 (d, 1H, J = 3.1 Hz), 7.39 – 7.42 (m, 2H), 7.47 – 7.50 (m, 1H), 7.73-7.74 (m, 2H), 9.84 (s, 1H). 13C {1H} NMR ($CDCl_3$, 125 MHz): δ 9.1, 28.2, 81.6, 103.8, 121.6, 121.8, 126.1, 128.0, 128.9, 131.1, 140.6, 153.5, 192.1. HRMS (ESI-TOF) m/z: [M + Na]+ Calcd for $C_{17}H_{20}N_2O_3Na$ 323.1372; Found 323.1374.

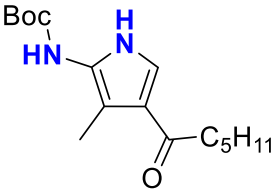

tert-Butyl (4-hexanoyl-3-methyl-1H-pyrrol-2-yl)carbamate (12l). (31.7 mg, 47%). Purified by silica gel chromatography (EtOAc/Hexanes: 15:85 v/v). Yellow oil: 1H NMR ($CDCl_3$, 500 MHz): δ 0.85-0.88 (m, 3H), 1.29-1.32 (m, 4H), 1.49 (s, 8H), 1.84-1.70 (m, 2H), 1.96 (s, 3H), 2.60 (t, 2H, J = 7.6 Hz), 6.61 (d, 1H, J = 2.7 Hz), 6.83 (bs, 1H), 10.31 (bs, 1H). 13C {1H} NMR ($CDCl_3$, 125 MHz): δ 9.5,

13.9, 22.5, 25.7, 28.1, 31.7, 37.3, 81.9, 103.8, 117.6, 125.1, 132.0, 152.1, 188.7. HRMS (ESI-TOF) m/z: [M + Na]+ Calcd for $C_{16}H_{26}N_2O_3Na$ 317.1841; Found 317.1839.

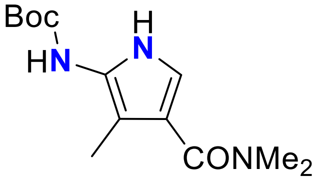

tert-Butyl (4-(dimethylcarbamoyl)-3-methyl-1H-pyrrol-2-yl)carbamate (12m). Purified by silica gel chromatography (EtOAc/Hexanes: 80:20 $^v/_v$). Pale brown foam (54.5 mg, 40%): 1H NMR ($CDCl_3$, 400 MHz): δ 9.51 (bs, 1H), 6.56 (d, 1H, J = 2.7 Hz), 6.49 (bs, 1H), 3.04 (s, 6H), 1.96 (s, 3H), 1.48 (s, 9H). 13C {1H} NMR ($CDCl_3$, 125 MHz): δ 168.5, 153.6, 124.3, 117.1, 112.9, 103.2, 81.1, 28.23 (3C), 28.18 (2C), 8.6. HRMS (ESI-TOF) m/z: [M + Na]+ Calcd for $C_{13}H_{21}N_3O_3Na$ 290.1481; Found 290.1476.

4. ^{1}H and ^{13}C NMR Spectra.

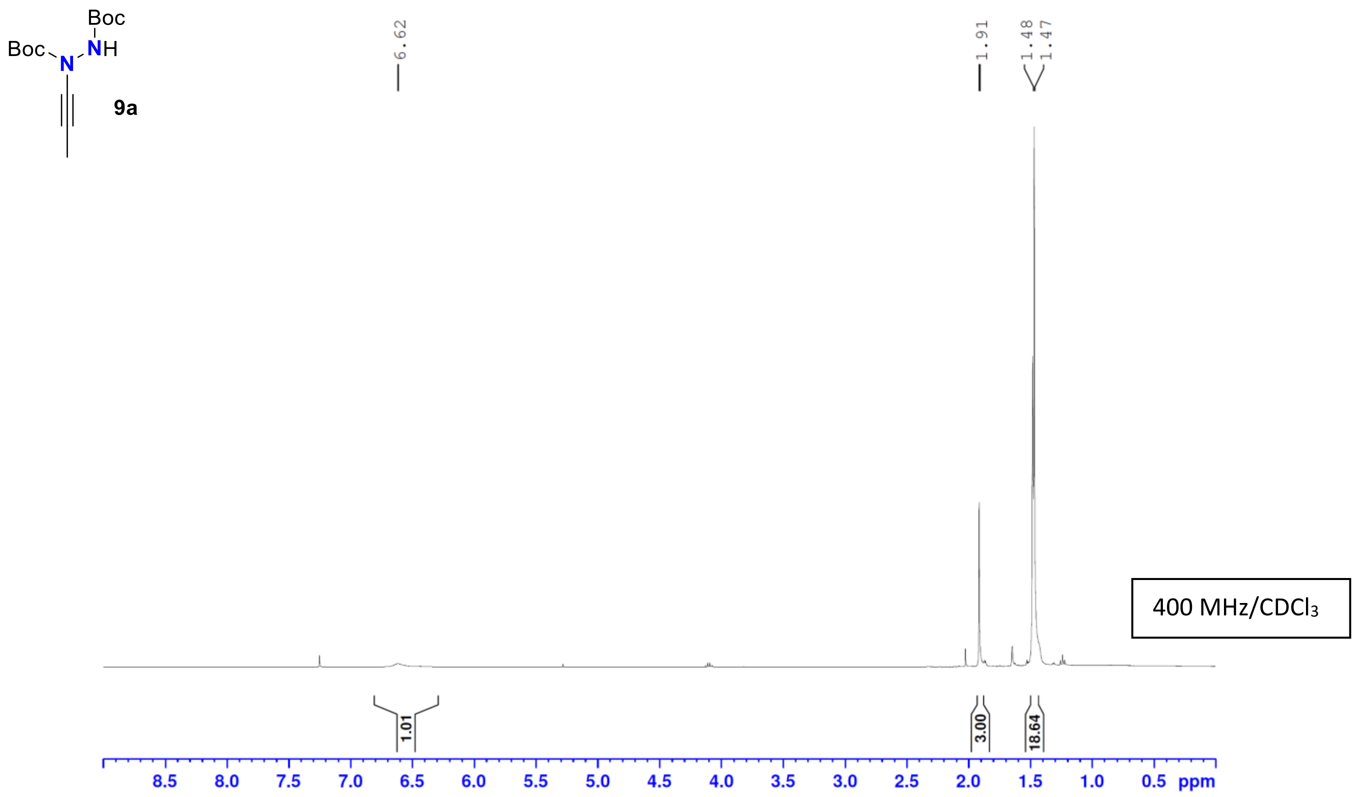

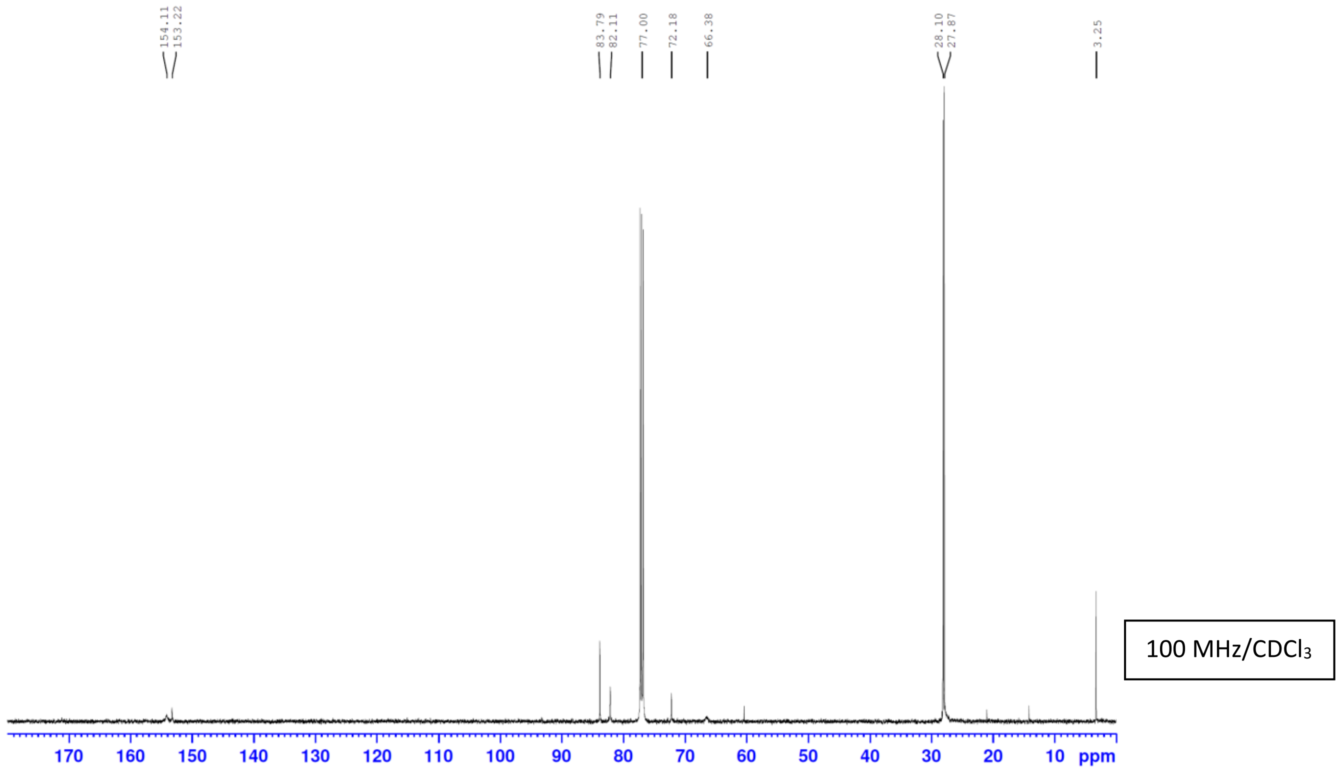

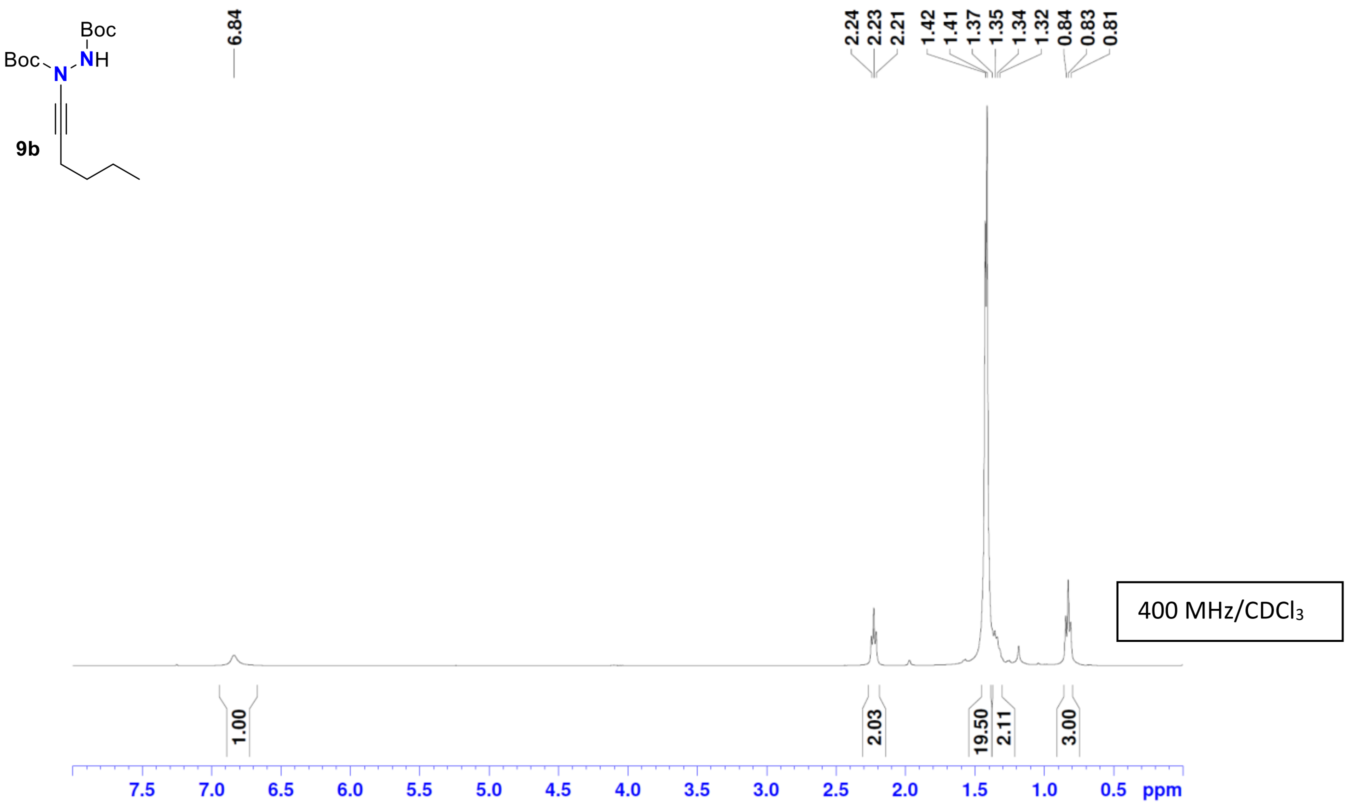
Boc
Boc
NH
N
9b
6.84
2.24
2.23
2.21
1.42
1.41
1.37
1.35
1.34
1.32
0.84
0.83
0.81
400 MHz/CDCl3
1.00
2.03
19.50
2.11
3.00
7.5
7.0
6.5
6.0
5.5
5.0
4.5
4.0
3.5
3.0
2.5
2.0
1.5
1.0
0.5
ppm

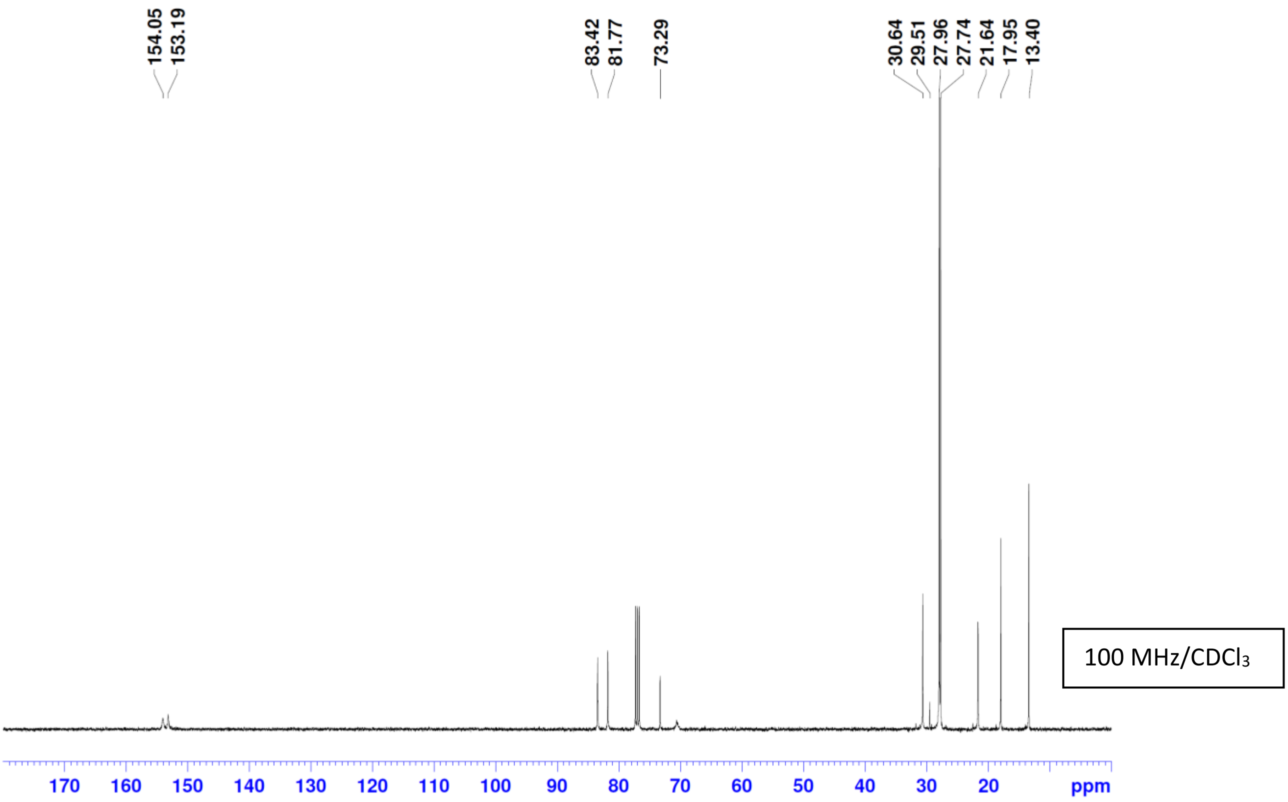
154.05
153.19
83.42
81.77
73.29
30.64
29.51
27.96
27.74
21.64
17.95
13.40
100 MHz/CDCl3
170
160
150
140
130
120
110
100
90
80
70
60
50
40
30
20
ppm

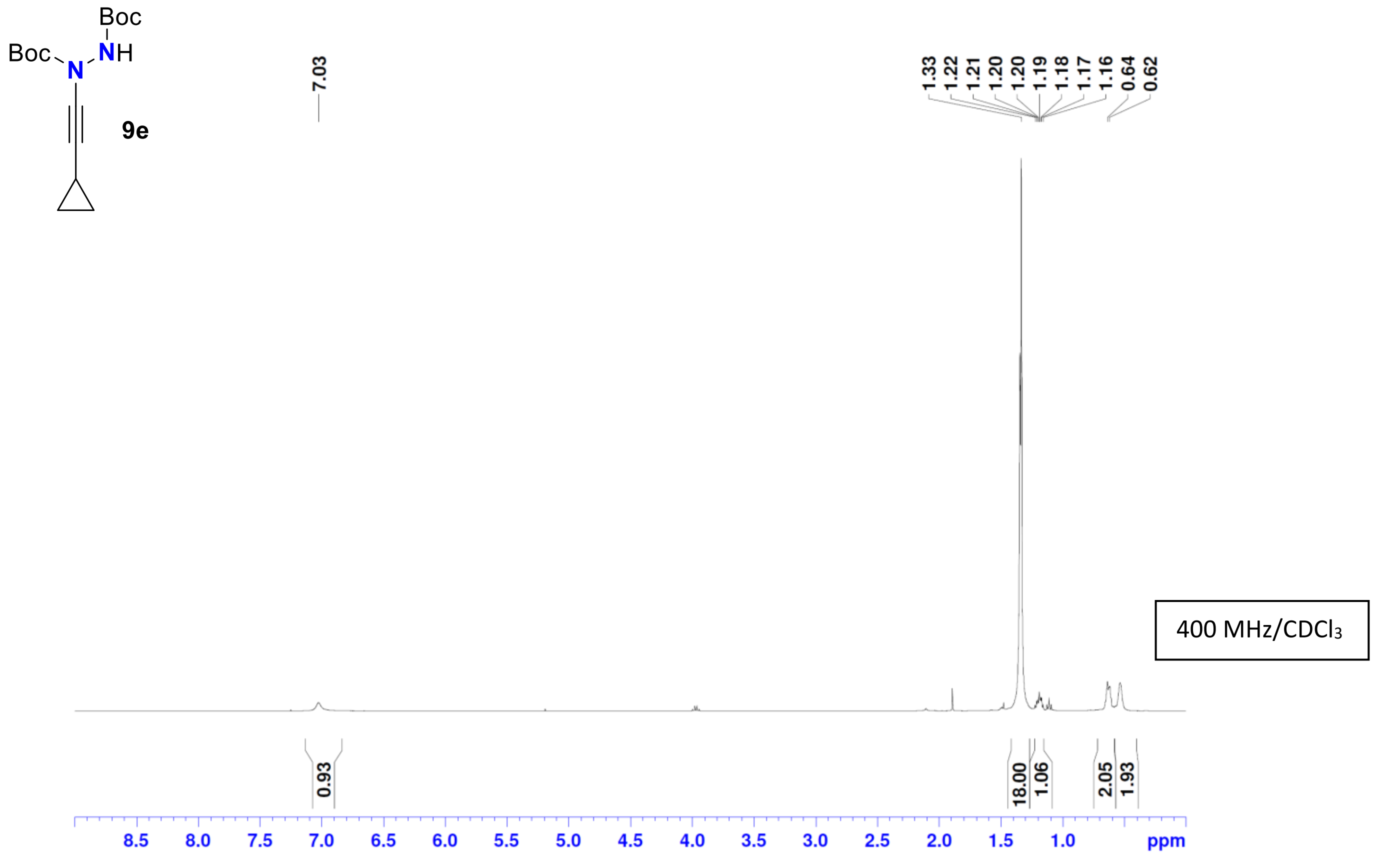
Boc
Boc
NH
N
9e
7.03
1.33
1.22
1.21
1.20
1.20
1.19
1.18
1.17
1.16
0.64
0.62
400 MHz/CDCl3
0.93
18.00
1.06
2.05
1.93
8.5 8.0 7.5 7.0 6.5 6.0 5.5 5.0 4.5 4.0 3.5 3.0 2.5 2.0 1.5 1.0 ppm

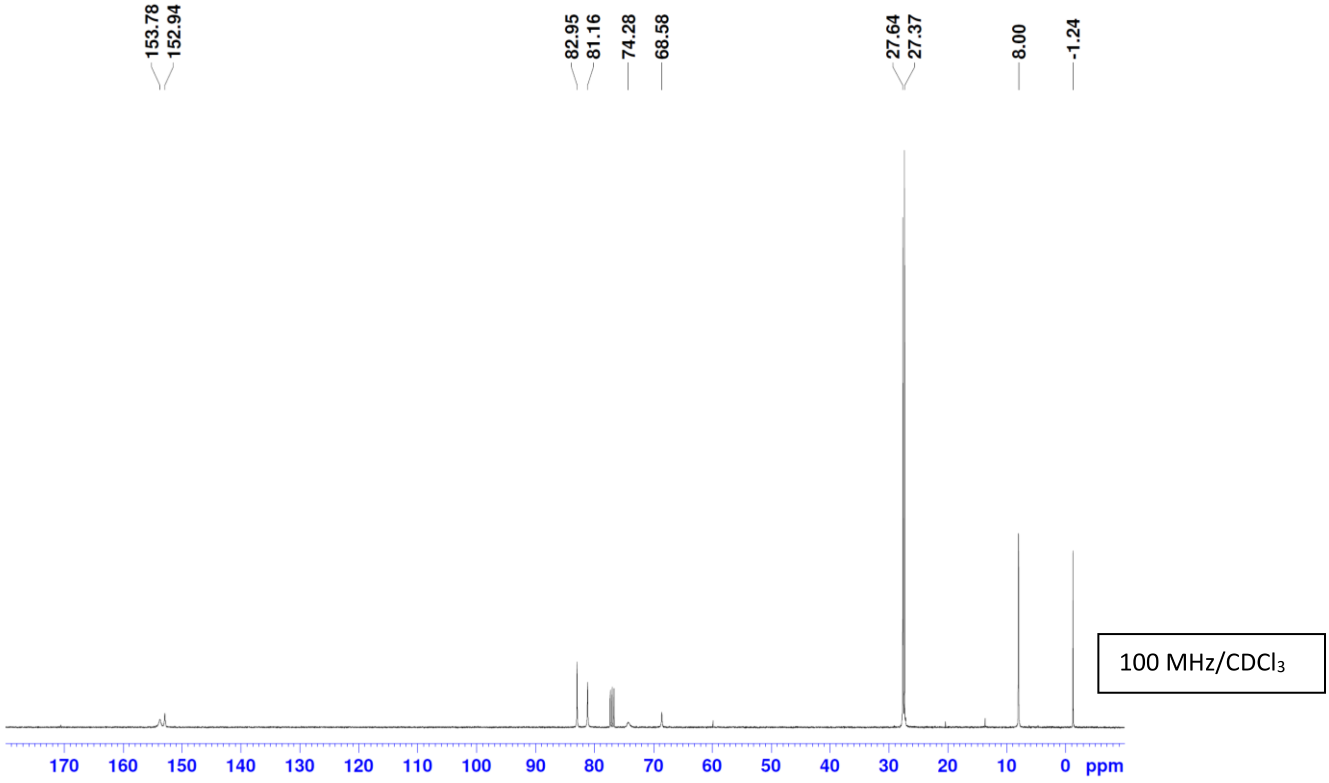
153.78
152.94
82.95
81.16
74.28
68.58
27.64
27.37
8.00
-1.24
100 MHz/CDCl3
170 160 150 140 130 120 110 100 90 80 70 60 50 40 30 20 10 0 ppm

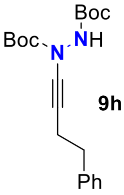
Boc
Boc
N
NH
Ph

9h

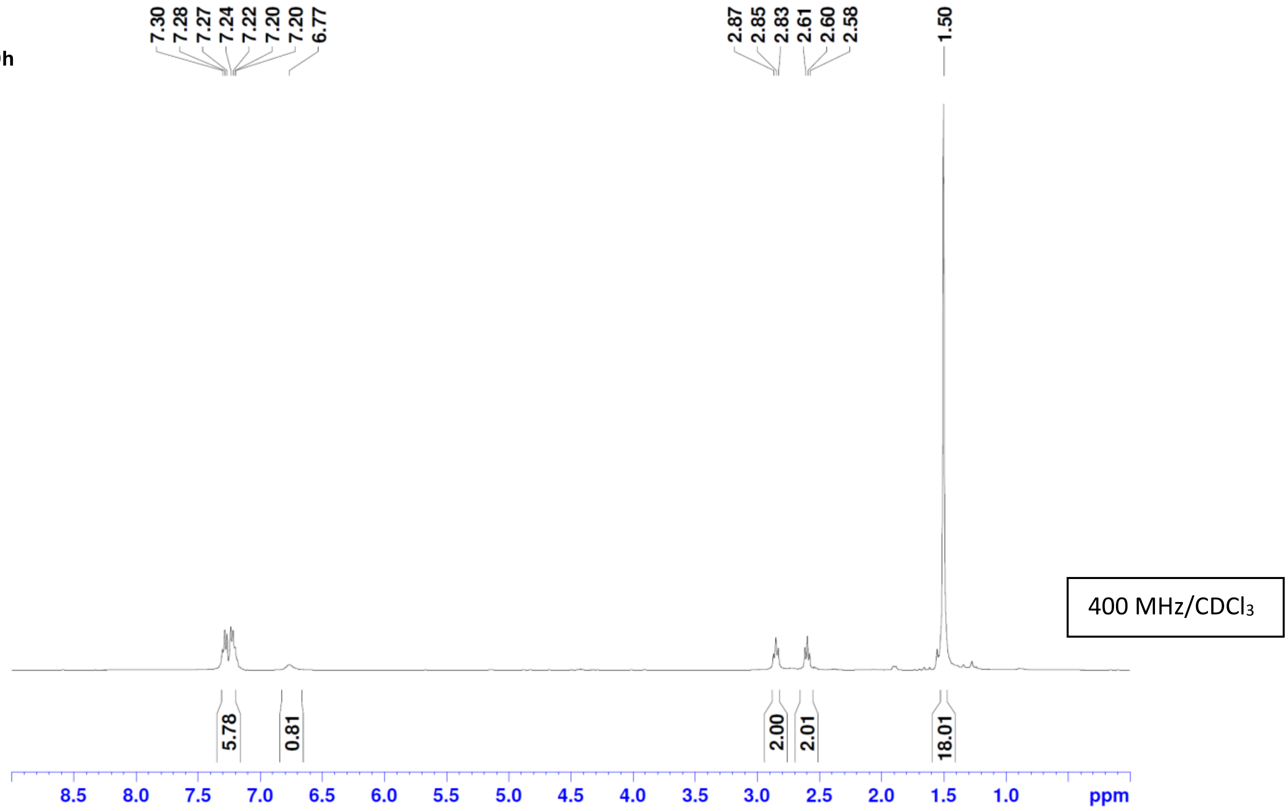
7.30
7.28
7.27
7.24
7.22
7.20
7.20
6.77
2.87
2.85
2.83
2.61
2.60
2.58
1.50
400 MHz/CDCl3
5.78
0.81
2.00
2.01
18.01
8.5
8.0
7.5
7.0
6.5
6.0
5.5
5.0
4.5
4.0
3.5
3.0
2.5
2.0
1.5
1.0
ppm

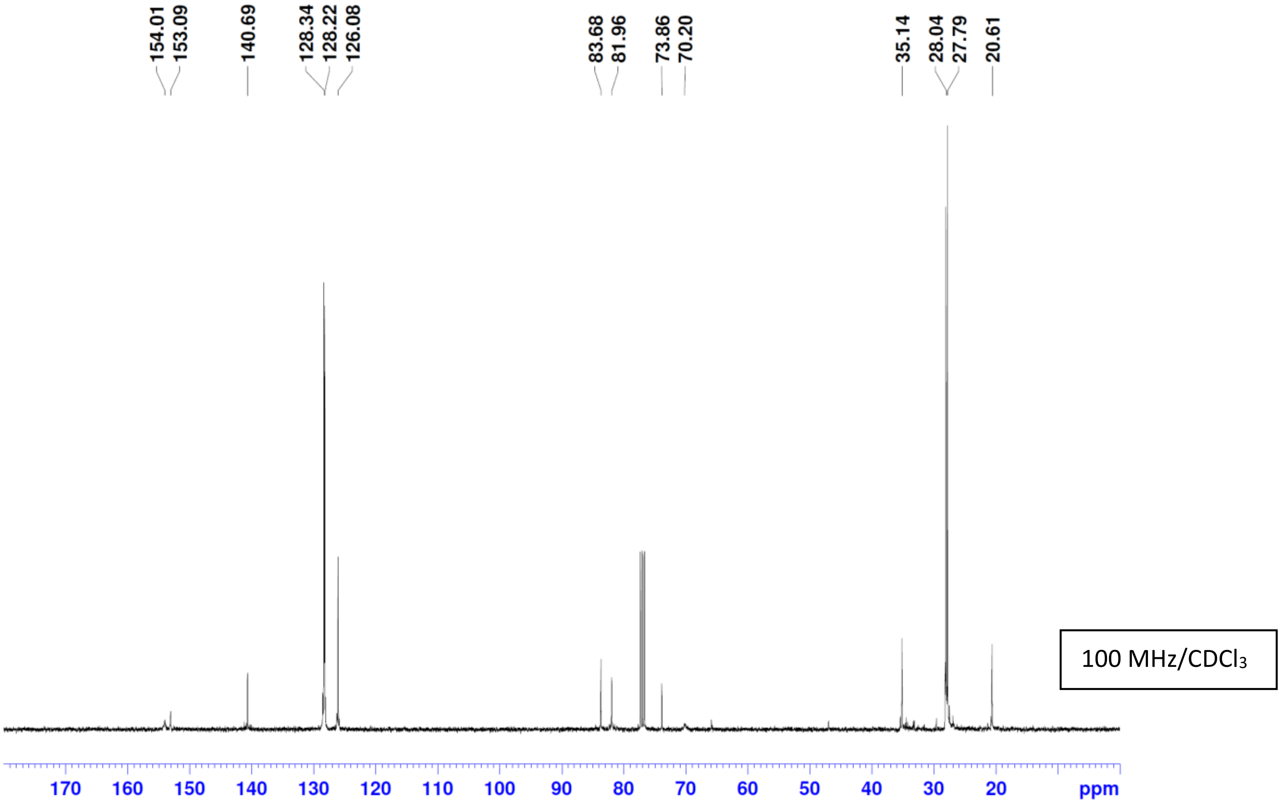
154.01
153.09
140.69
128.34
128.22
126.08
83.68
81.96
73.86
70.20
35.14
28.04
27.79
20.61
100 MHz/CDCl3
170
160
150
140
130
120
110
100
90
80
70
60
50
40
30
20
ppm

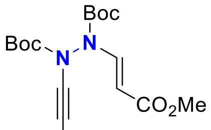
Boc
Boc
N
N
CO2Me

4a

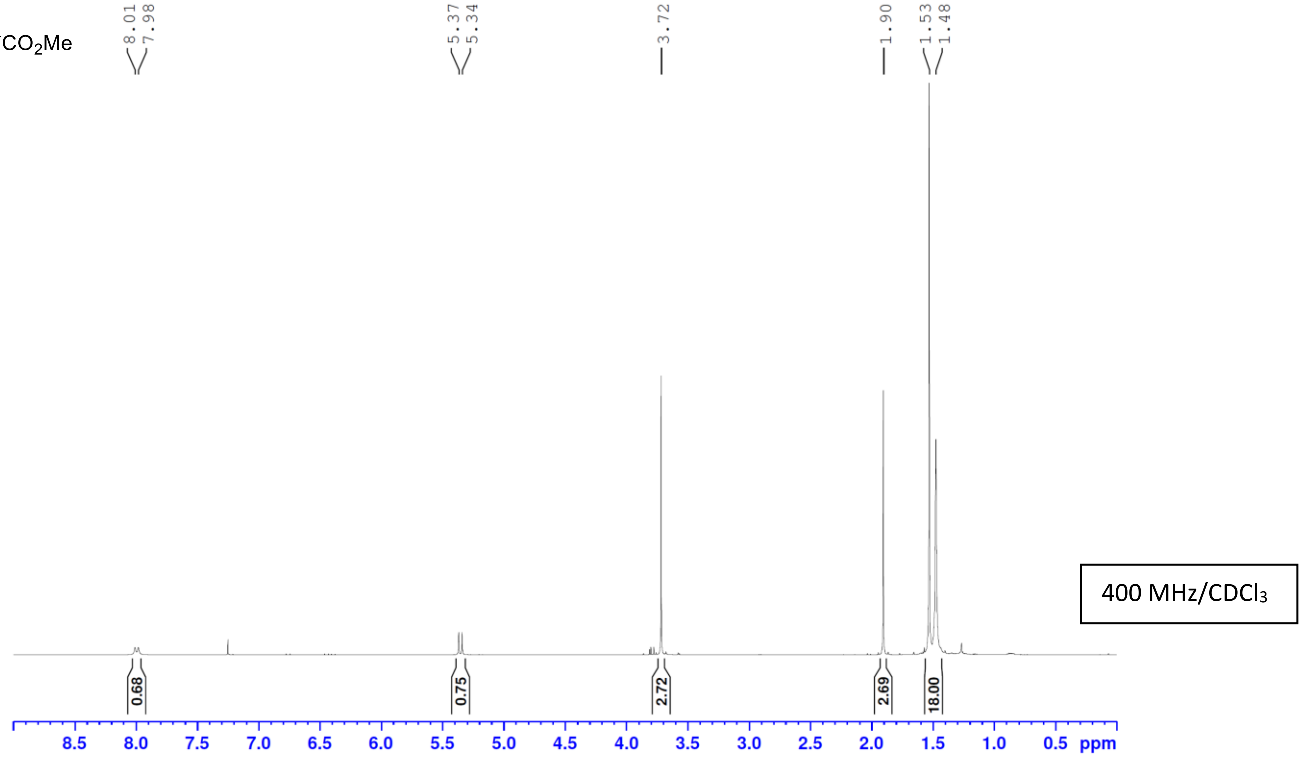
8.01
7.98
5.37
5.34
3.72
1.90
1.53
1.48
0.68
0.75
2.72
2.69
18.00
8.5 8.0 7.5 7.0 6.5 6.0 5.5 5.0 4.5 4.0 3.5 3.0 2.5 2.0 1.5 1.0 0.5 ppm
400 MHz/CDCl3

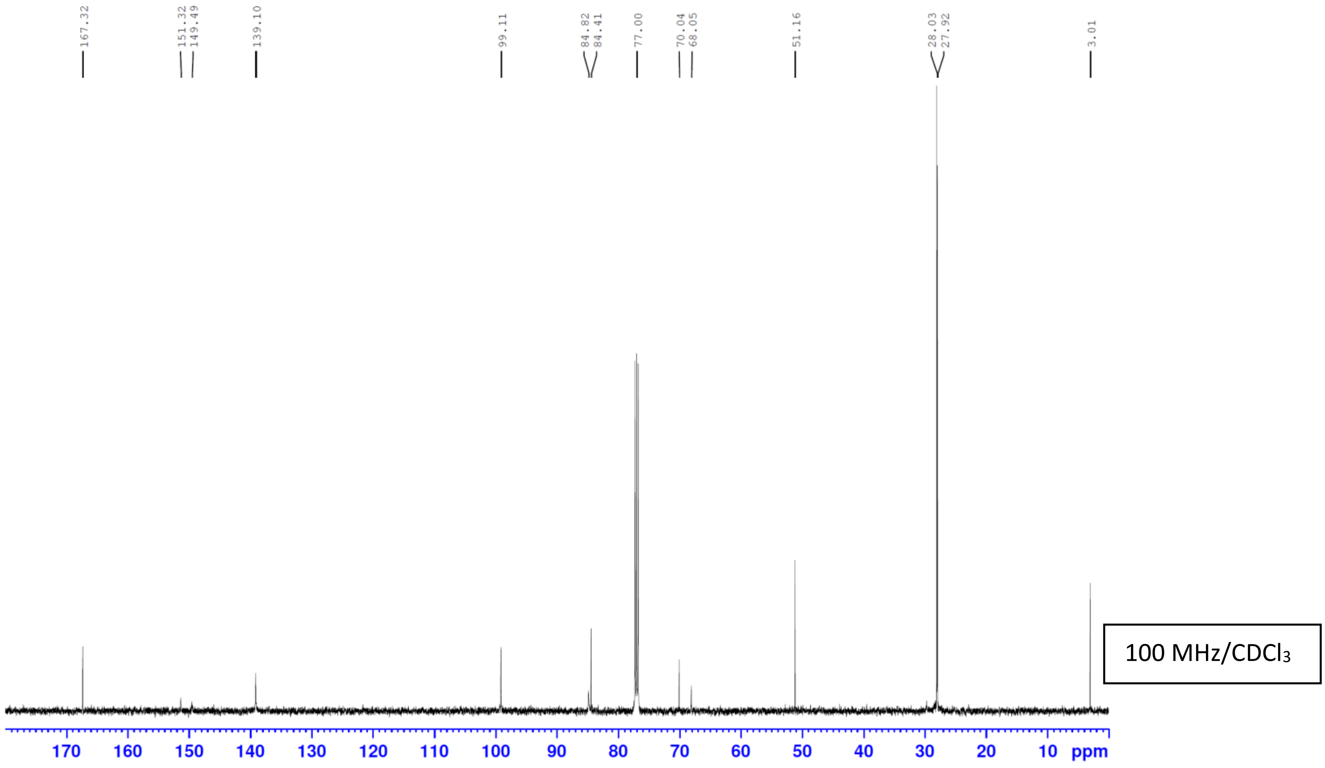
167.32
151.32
149.49
139.10
99.11
84.82
84.41
77.00
70.04
68.05
51.16
28.03
27.92
3.01
170 160 150 140 130 120 110 100 90 80 70 60 50 40 30 20 10 ppm
100 MHz/CDCl3

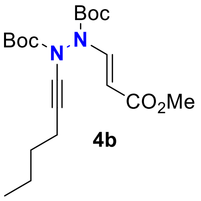
Boc
Boc
N
N
CO$_2$Me

4b

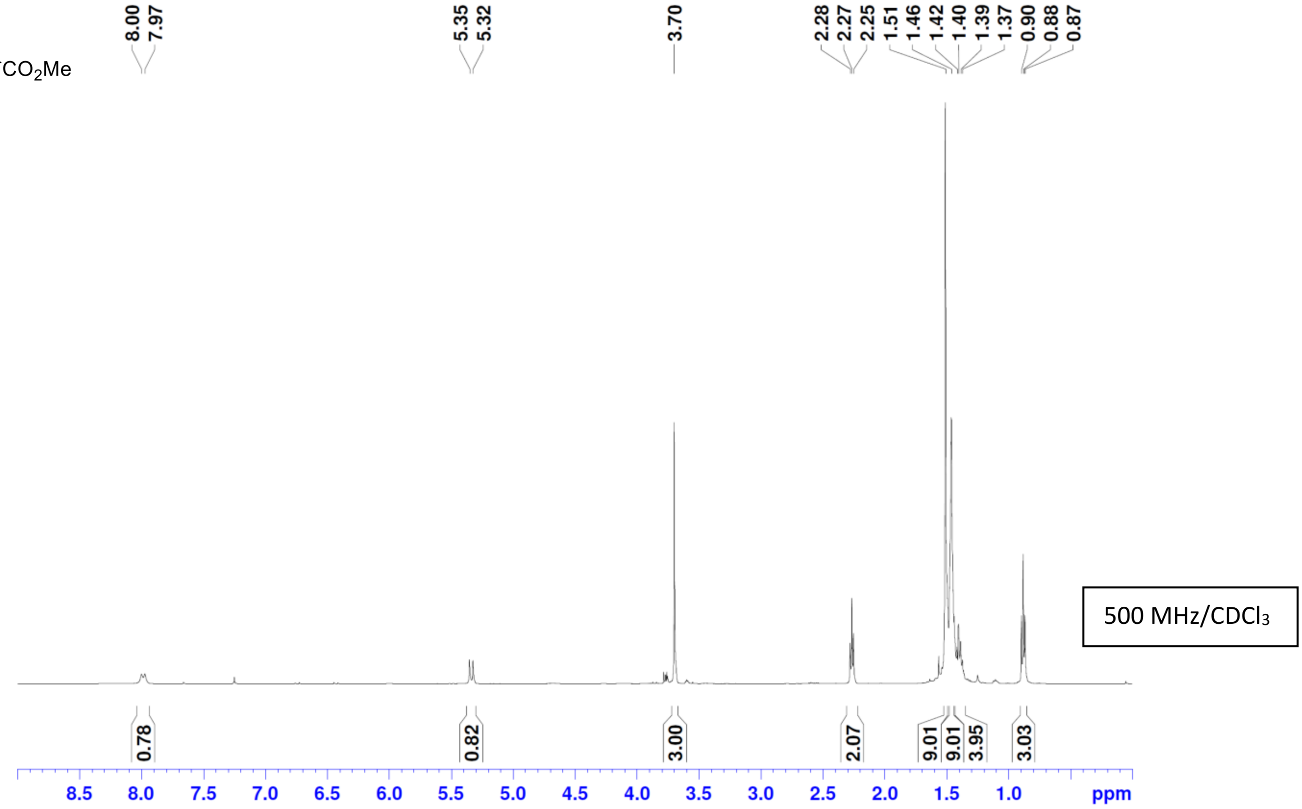
8.00
7.97
5.35
5.32
3.70
2.28
2.27
2.25
1.51
1.46
1.42
1.40
1.39
1.37
0.90
0.88
0.87
500 MHz/CDCl$_3$
0.78
0.82
3.00
2.07
9.01
9.01
3.95
3.03
8.5 8.0 7.5 7.0 6.5 6.0 5.5 5.0 4.5 4.0 3.5 3.0 2.5 2.0 1.5 1.0 ppm

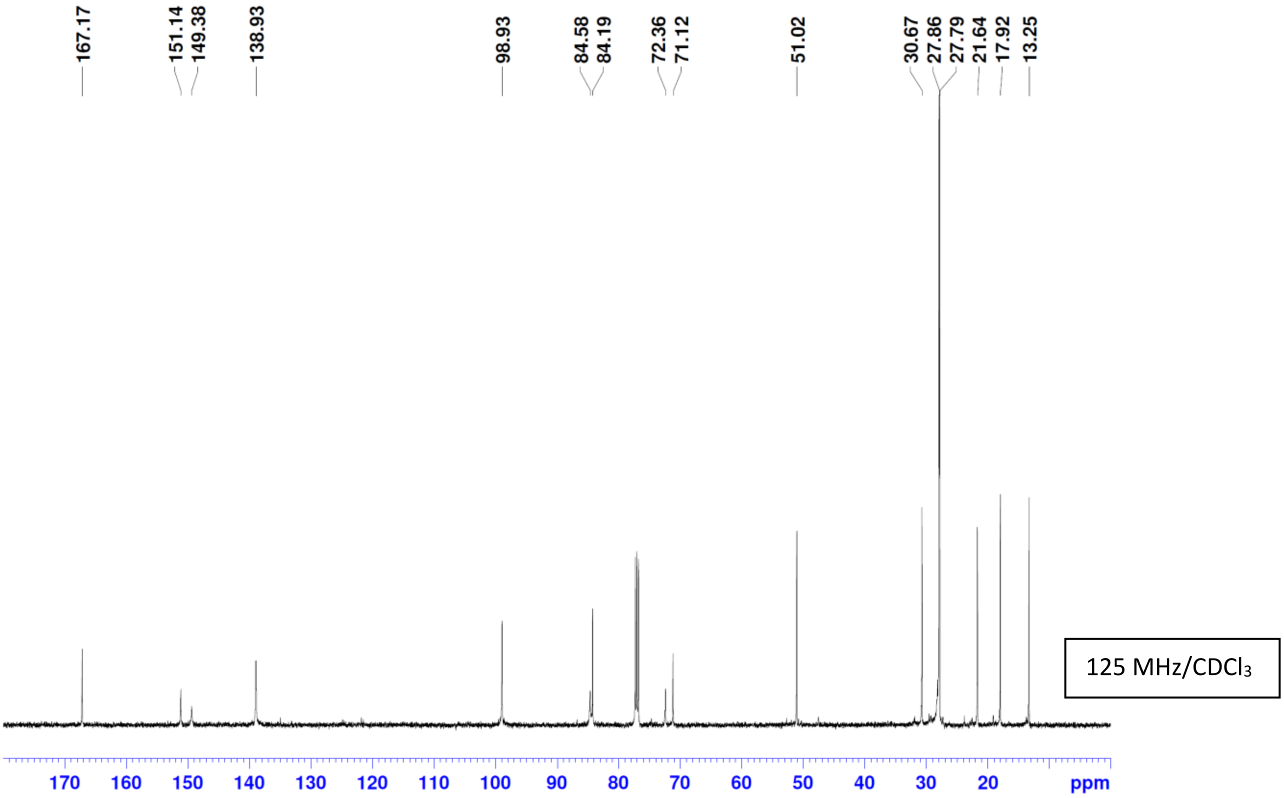
167.17
151.14
149.38
138.93
98.93
84.58
84.19
72.36
71.12
51.02
30.67
27.86
27.79
21.64
17.92
13.25
125 MHz/CDCl$_3$
170 160 150 140 130 120 110 100 90 80 70 60 50 40 30 20 ppm

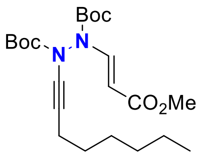
Boc
Boc
N
N
CO2Me

4c

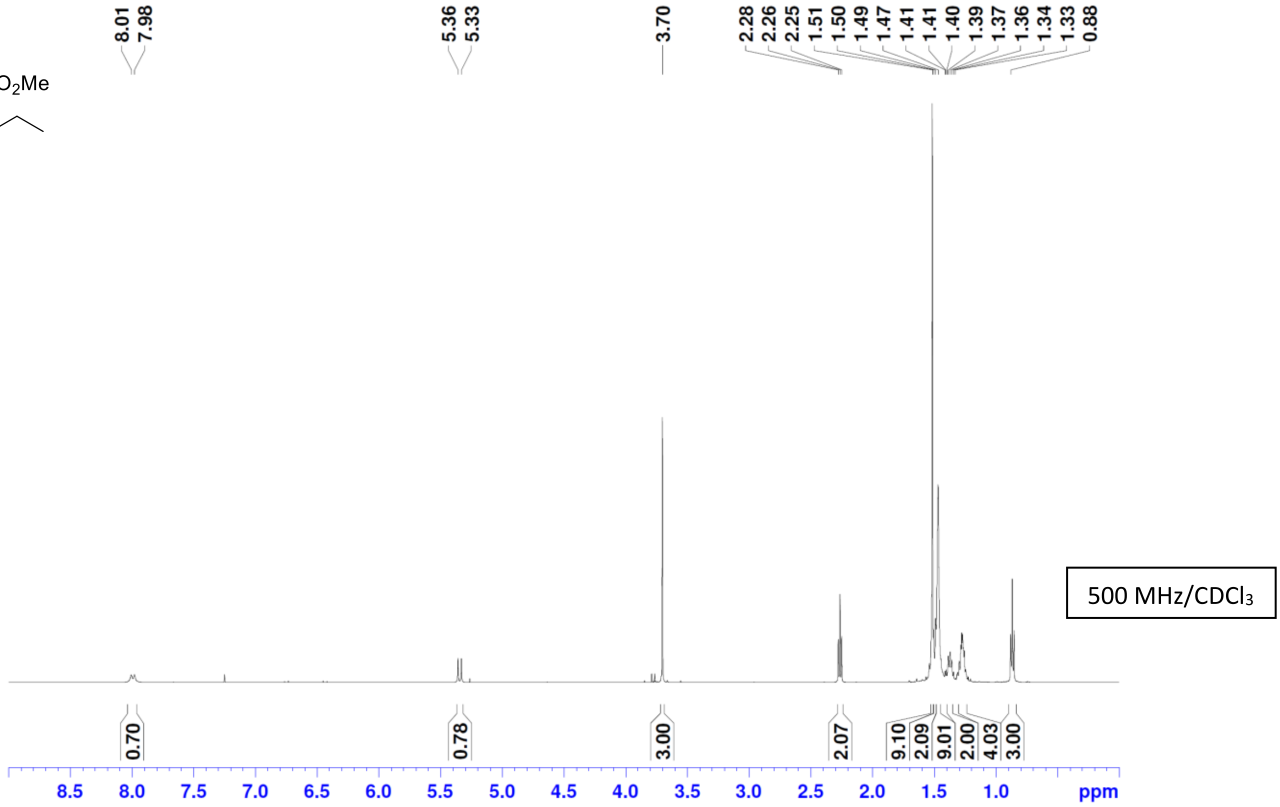
8.01
7.98
5.36
5.33
3.70
2.28
2.26
2.25
1.51
1.50
1.49
1.47
1.41
1.41
1.40
1.39
1.37
1.36
1.34
1.33
0.88
0.70
0.78
3.00
2.07
9.10
2.09
9.01
2.00
4.03
3.00
8.5 8.0 7.5 7.0 6.5 6.0 5.5 5.0 4.5 4.0 3.5 3.0 2.5 2.0 1.5 1.0 ppm
500 MHz/CDCl3

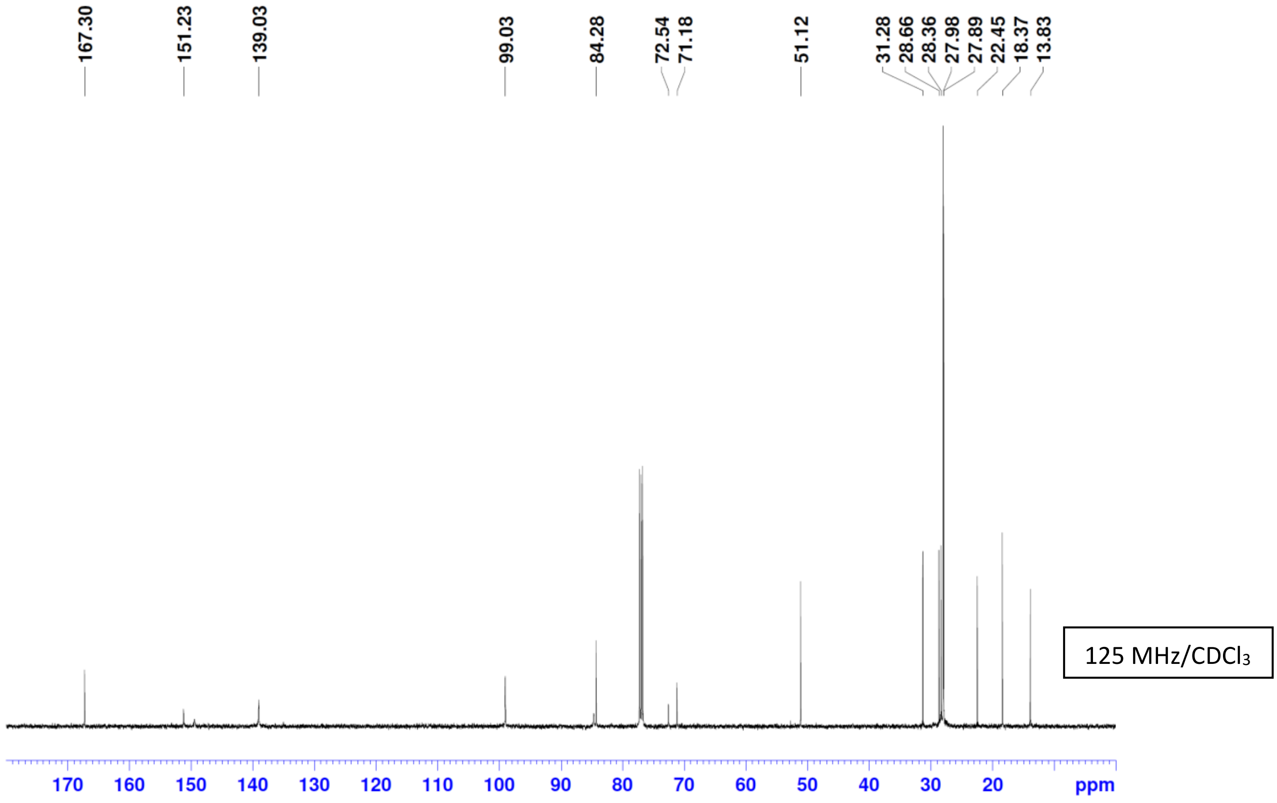
167.30
151.23
139.03
99.03
84.28
72.54
71.18
51.12
31.28
28.66
28.36
27.98
27.89
22.45
18.37
13.83
170 160 150 140 130 120 110 100 90 80 70 60 50 40 30 20 ppm
125 MHz/CDCl3

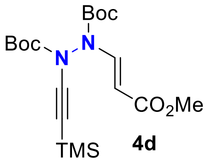
Boc
Boc
N
N
CO2Me
TMS

4d

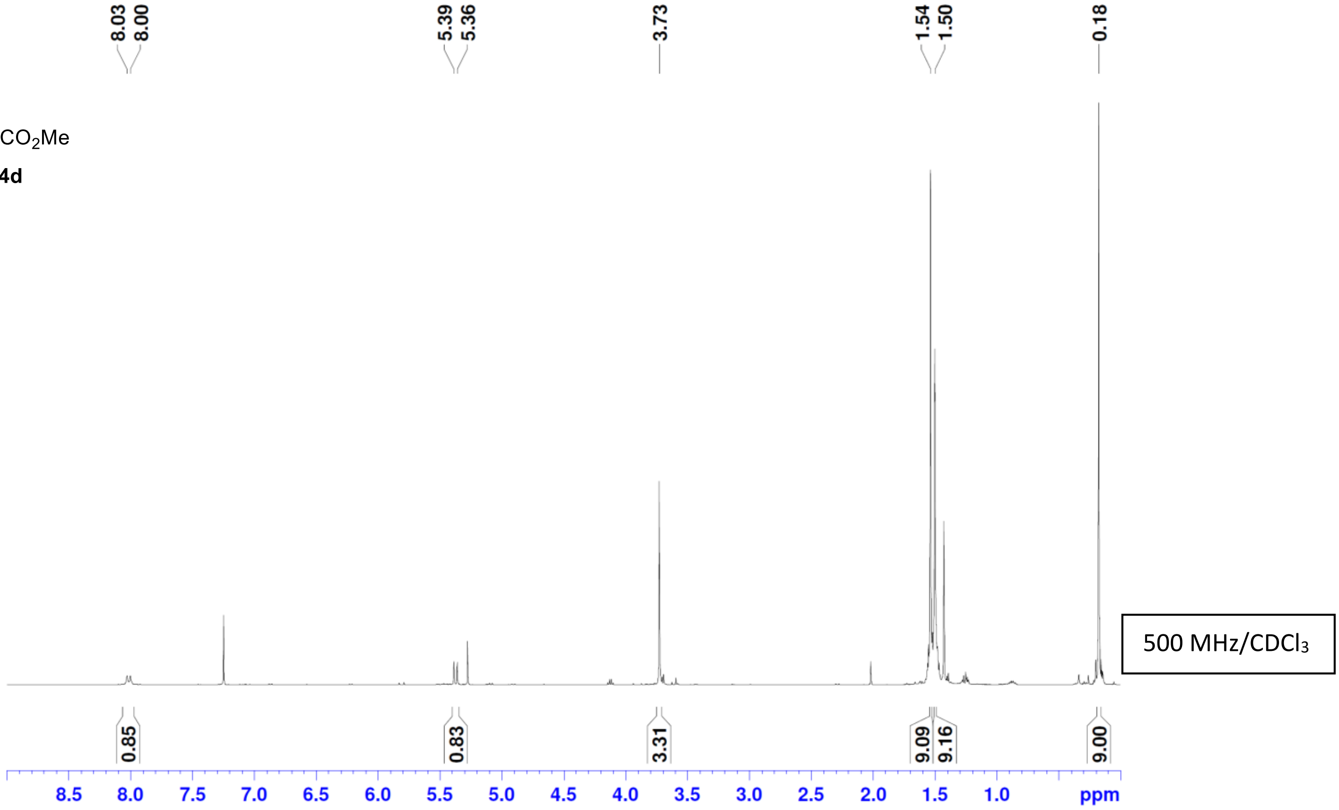
8.03
8.00
5.39
5.36
3.73
1.54
1.50
0.18
500 MHz/CDCl3
0.85
0.83
3.31
9.09
9.16
9.00
8.5 8.0 7.5 7.0 6.5 6.0 5.5 5.0 4.5 4.0 3.5 3.0 2.5 2.0 1.5 1.0 ppm

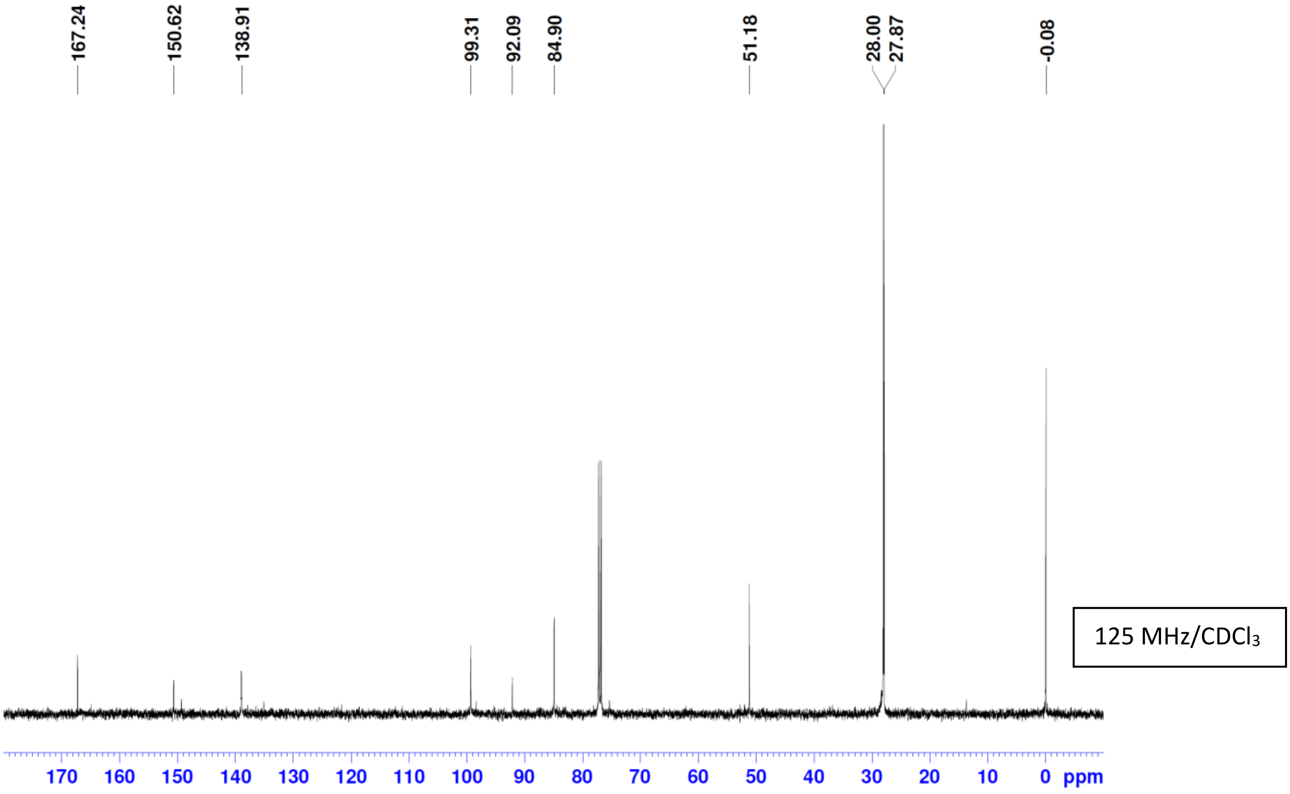
167.24
150.62
138.91
99.31
92.09
84.90
51.18
28.00
27.87
-0.08
125 MHz/CDCl3
170 160 150 140 130 120 110 100 90 80 70 60 50 40 30 20 10 0 ppm

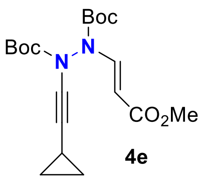

4e

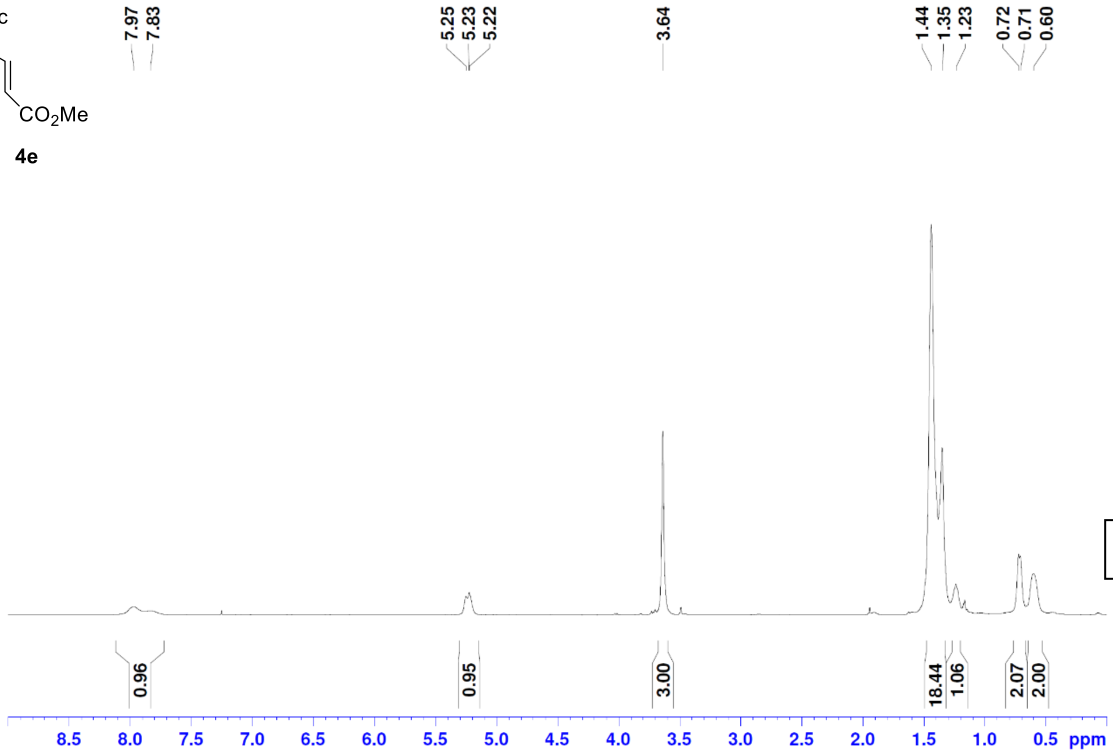

400 MHz/$CDCl_3$

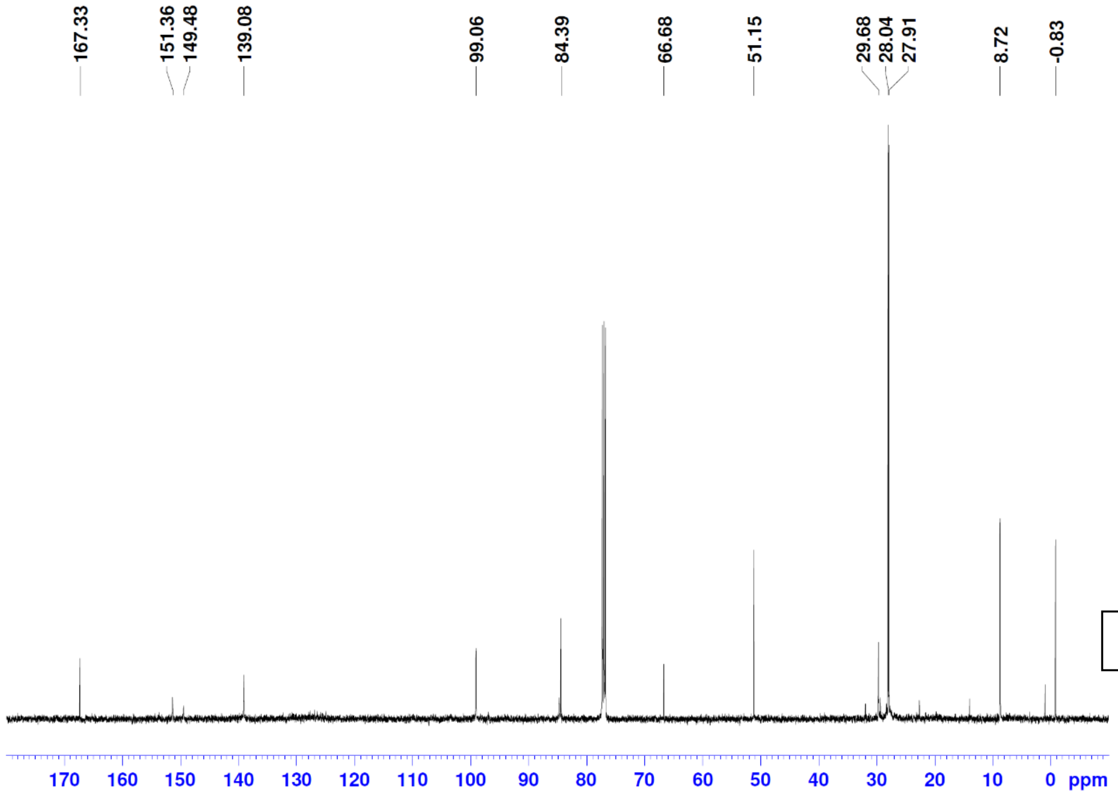

100 MHz/$CDCl_3$

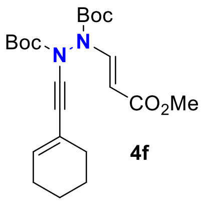
Boc
Boc
N
N
CO_2Me

4f

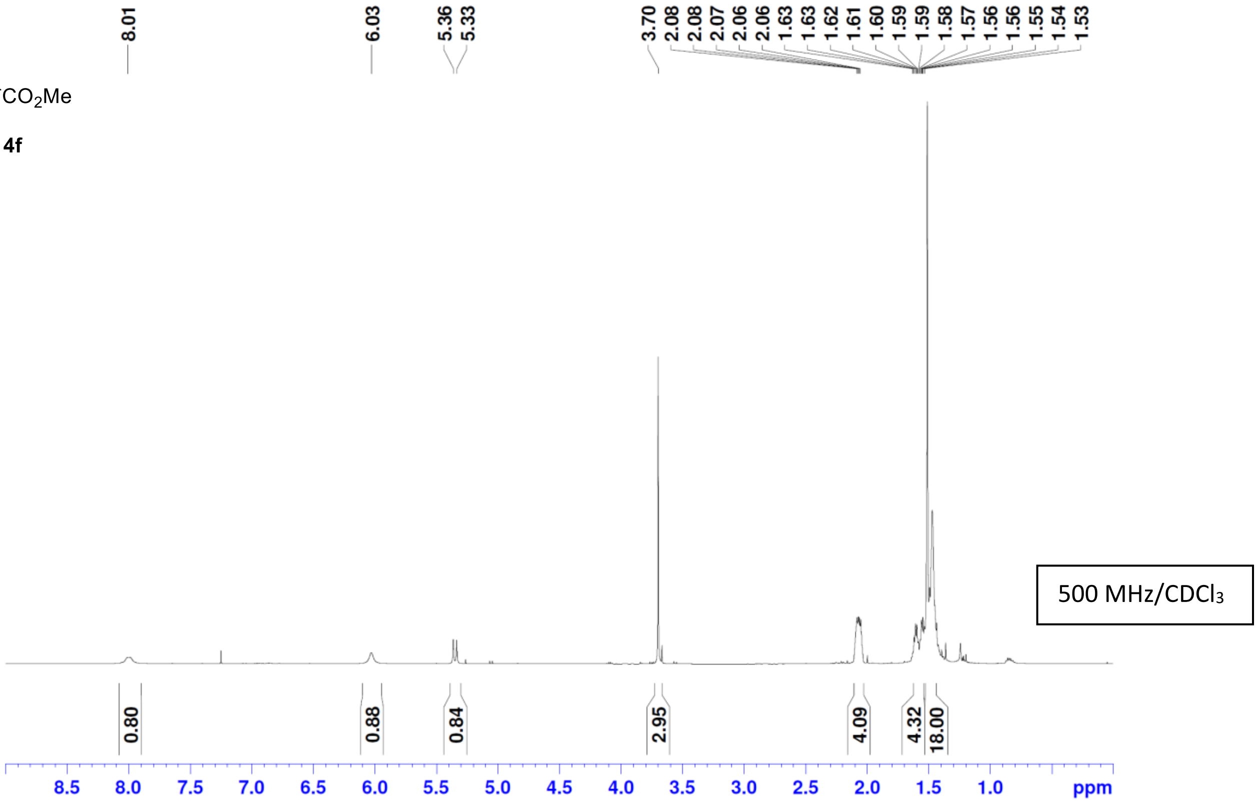
8.01
6.03
5.36
5.33
3.70
2.08
2.08
2.07
2.06
2.06
1.63
1.63
1.62
1.61
1.60
1.59
1.59
1.58
1.57
1.56
1.56
1.55
1.54
1.53
500 MHz/$CDCl_3$
0.80
0.88
0.84
2.95
4.09
4.32
18.00
8.5
8.0
7.5
7.0
6.5
6.0
5.5
5.0
4.5
4.0
3.5
3.0
2.5
2.0
1.5
1.0
ppm

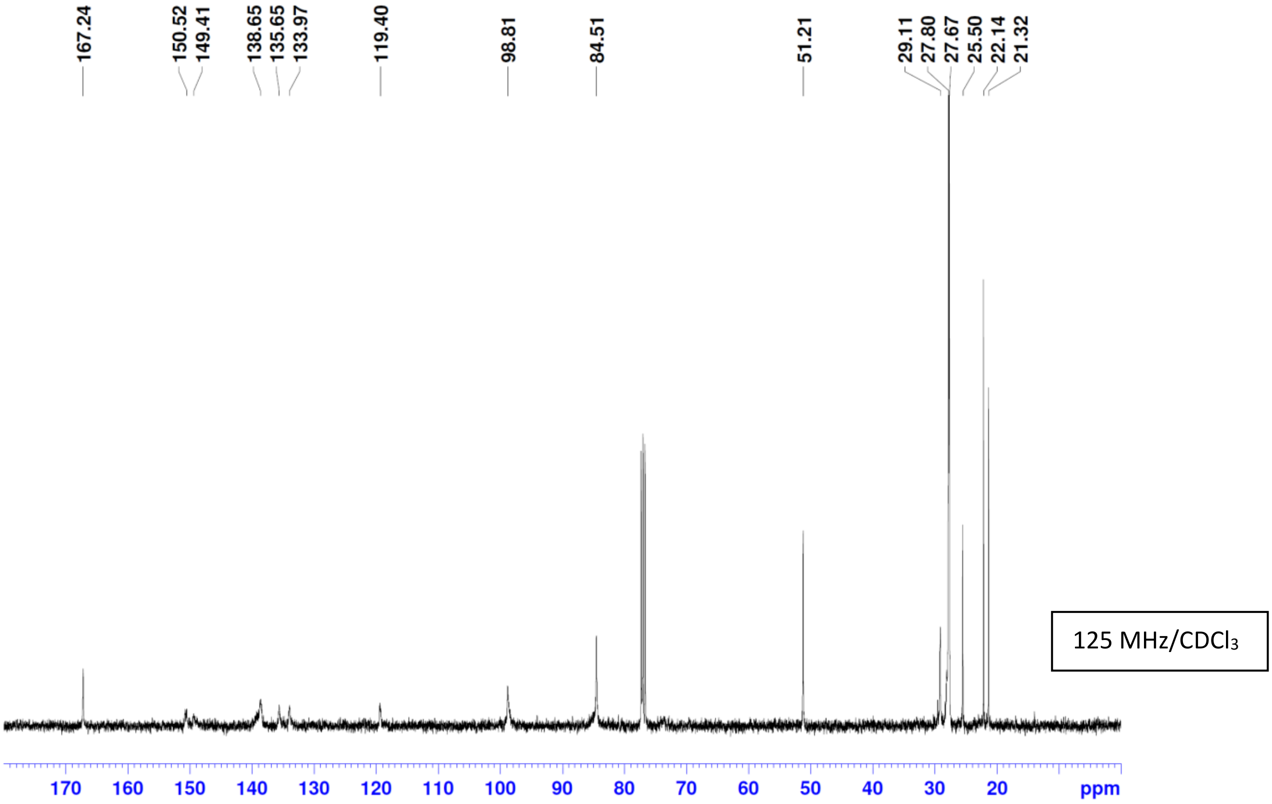
167.24
150.52
149.41
138.65
135.65
133.97
119.40
98.81
84.51
51.21
29.11
27.80
27.67
25.50
22.14
21.32
125 MHz/$CDCl_3$
170
160
150
140
130
120
110
100
90
80
70
60
50
40
30
20
ppm

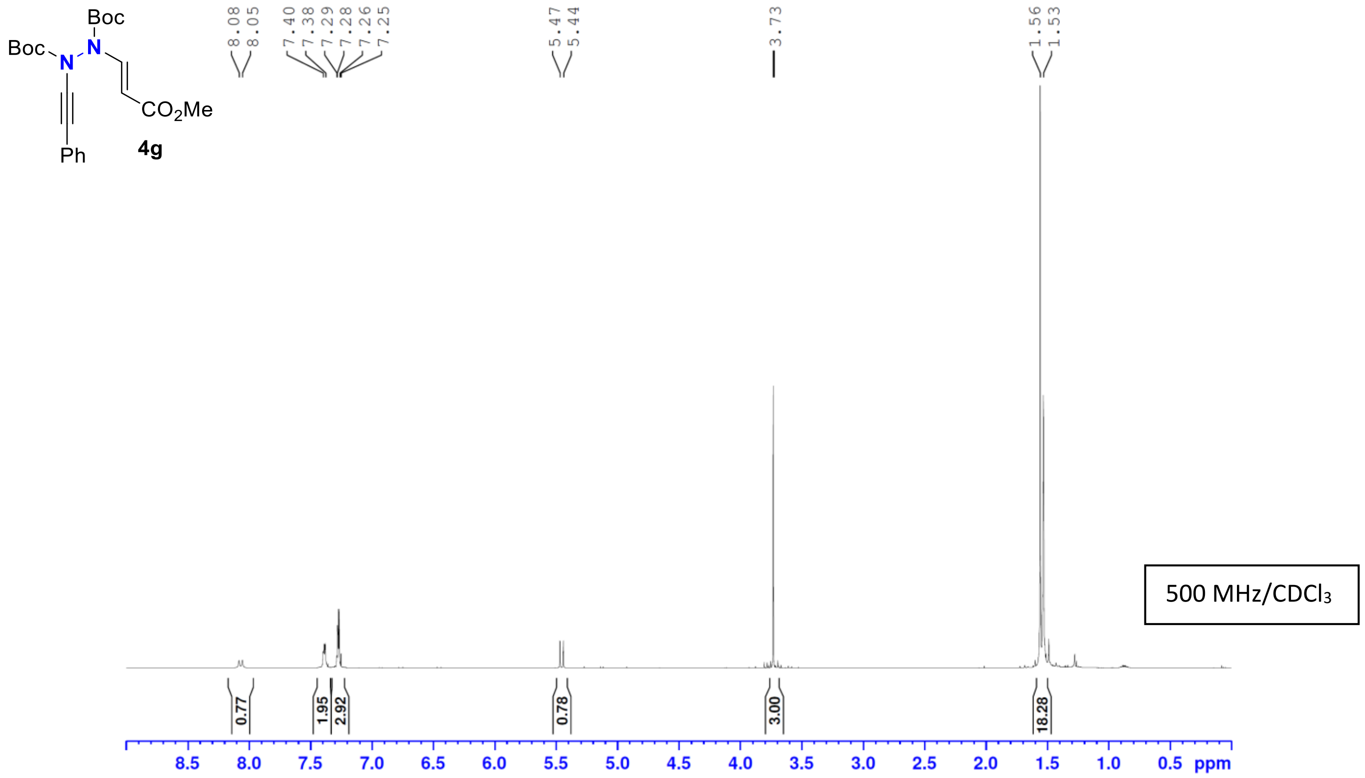

Boc
Boc
N
N
CO2Me
Ph
4g
8.08
8.05
7.40
7.38
7.29
7.28
7.26
7.25
5.47
5.44
3.73
1.56
1.53
500 MHz/CDCl3
0.77
1.95
2.92
0.78
3.00
18.28
8.5 8.0 7.5 7.0 6.5 6.0 5.5 5.0 4.5 4.0 3.5 3.0 2.5 2.0 1.5 1.0 0.5 ppm

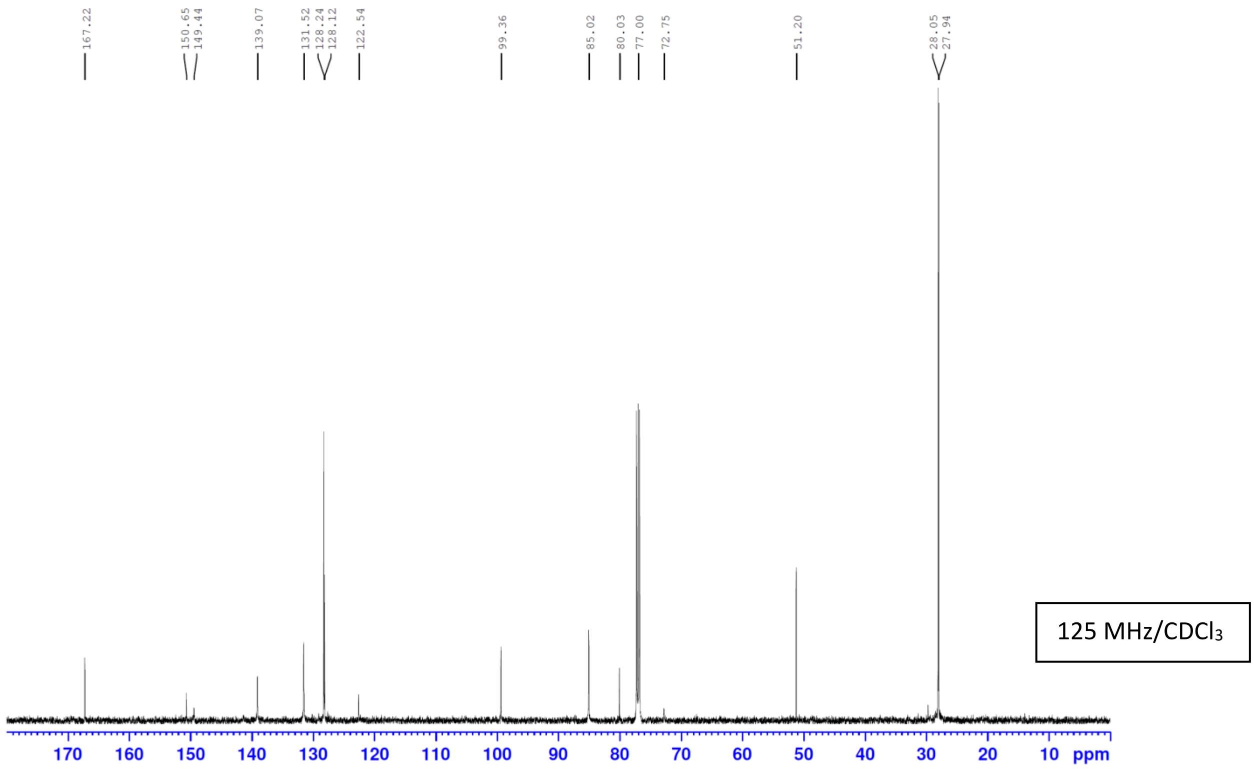

167.22
150.65
149.44
139.07
131.52
128.24
128.12
122.54
99.36
85.02
80.03
77.00
72.75
51.20
28.05
27.94
125 MHz/CDCl3
170 160 150 140 130 120 110 100 90 80 70 60 50 40 30 20 10 ppm

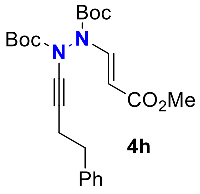
Boc
Boc
N
N
CO2Me
Ph

4h

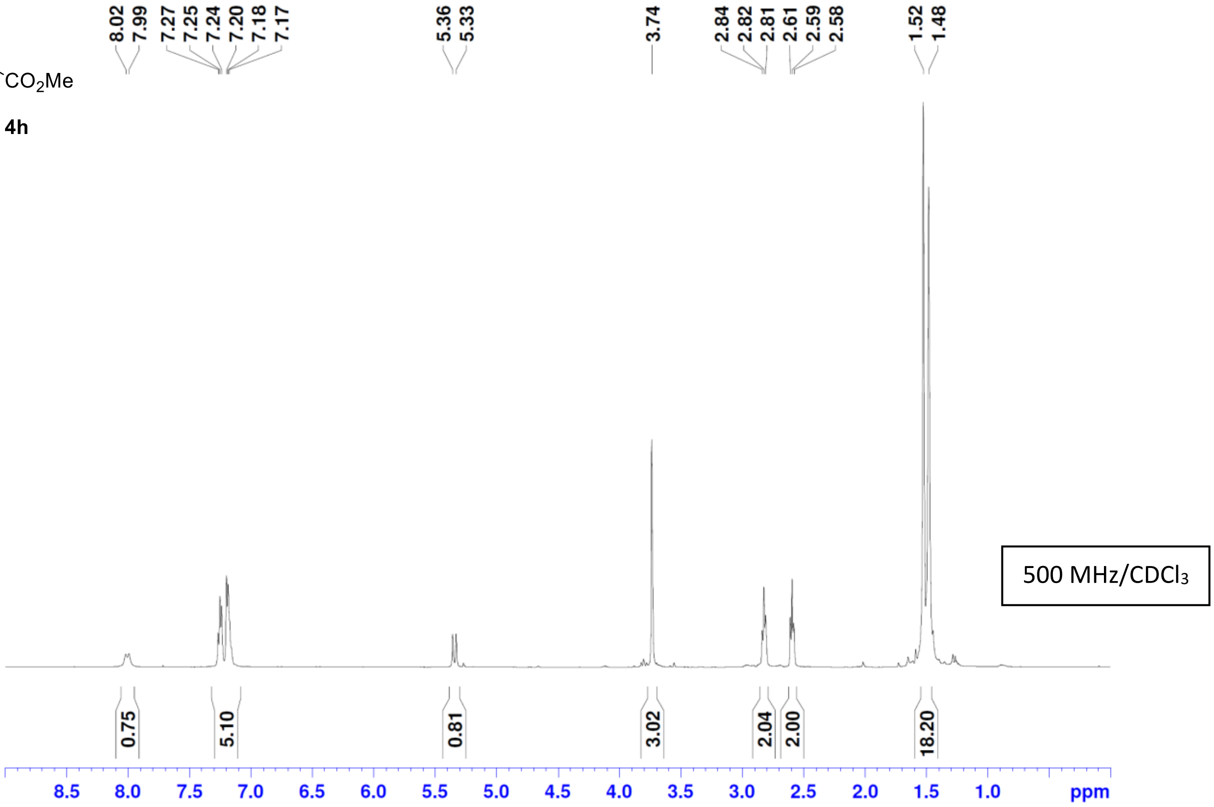
8.02
7.99
7.27
7.25
7.24
7.20
7.18
7.17
5.36
5.33
3.74
2.84
2.82
2.81
2.61
2.59
2.58
1.52
1.48
500 MHz/CDCl3
0.75
5.10
0.81
3.02
2.04
2.00
18.20
8.5 8.0 7.5 7.0 6.5 6.0 5.5 5.0 4.5 4.0 3.5 3.0 2.5 2.0 1.5 1.0 ppm

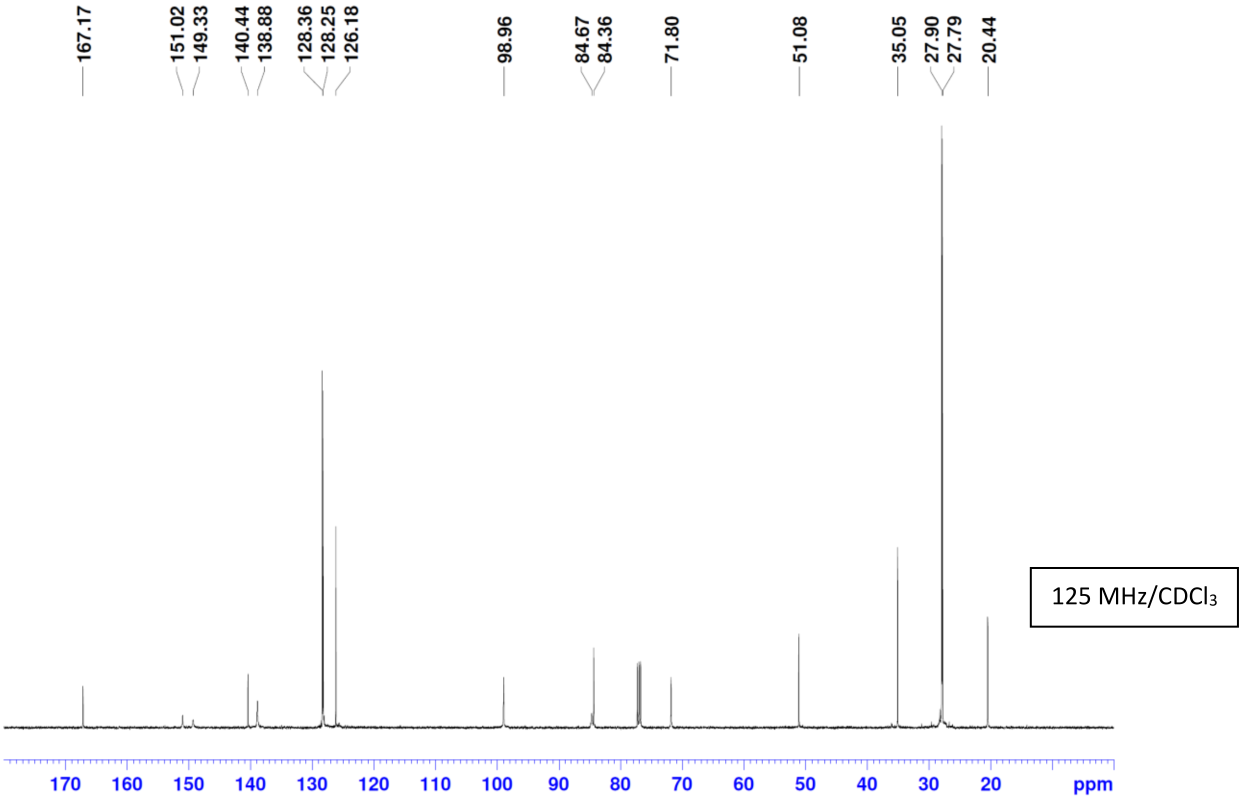
167.17
151.02
149.33
140.44
138.88
128.36
128.25
126.18
98.96
84.67
84.36
71.80
51.08
35.05
27.90
27.79
20.44
125 MHz/CDCl3
170 160 150 140 130 120 110 100 90 80 70 60 50 40 30 20 ppm

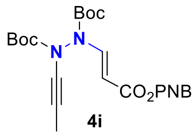

4i

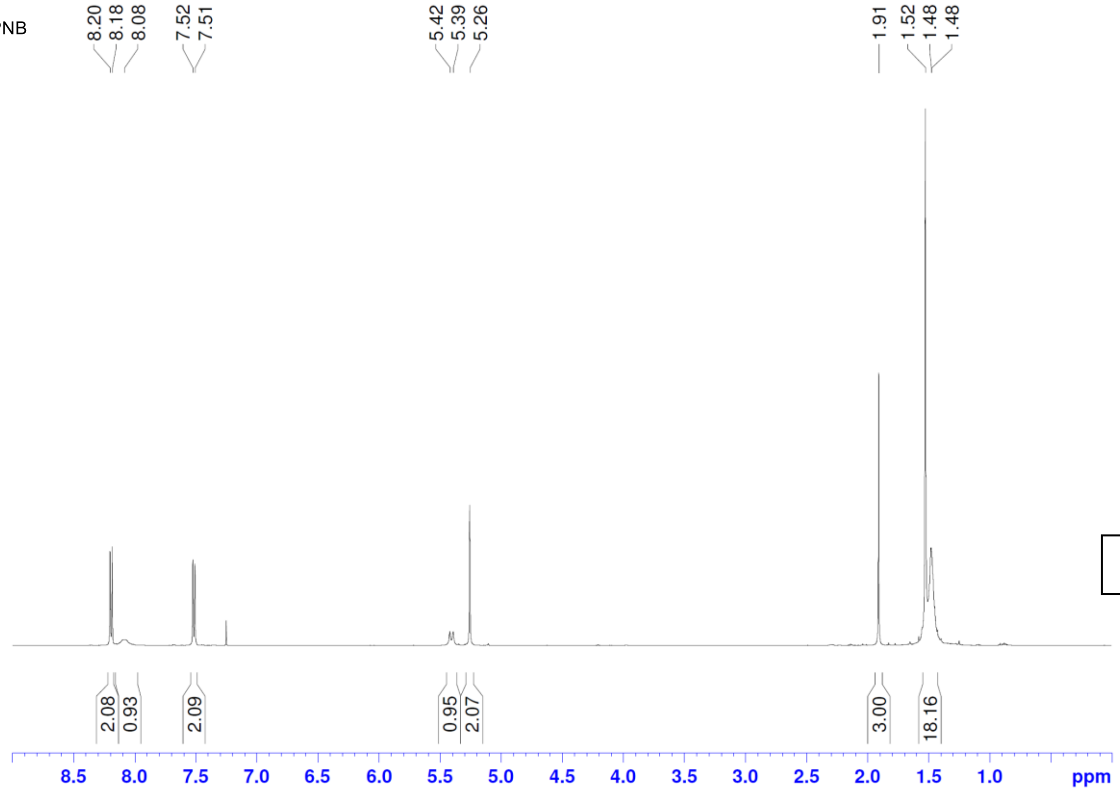

500 MHz/CDCl₃

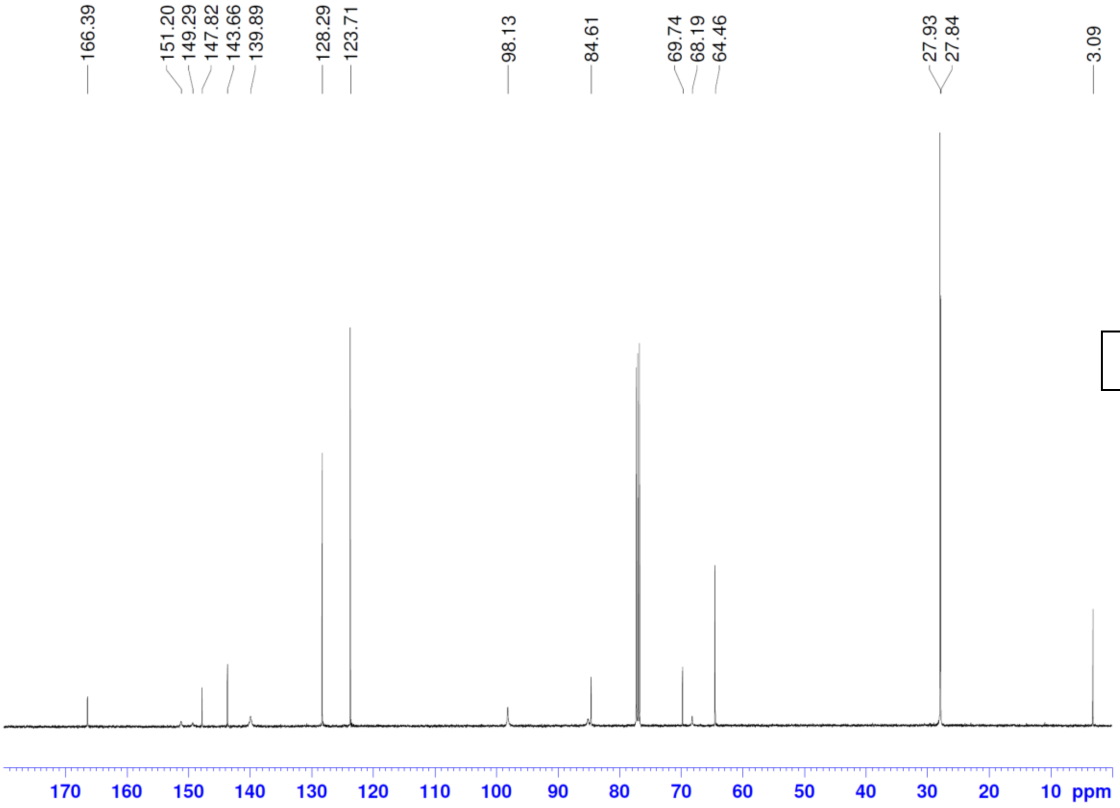

125 MHz/CDCl₃

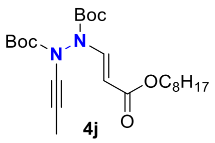

4j

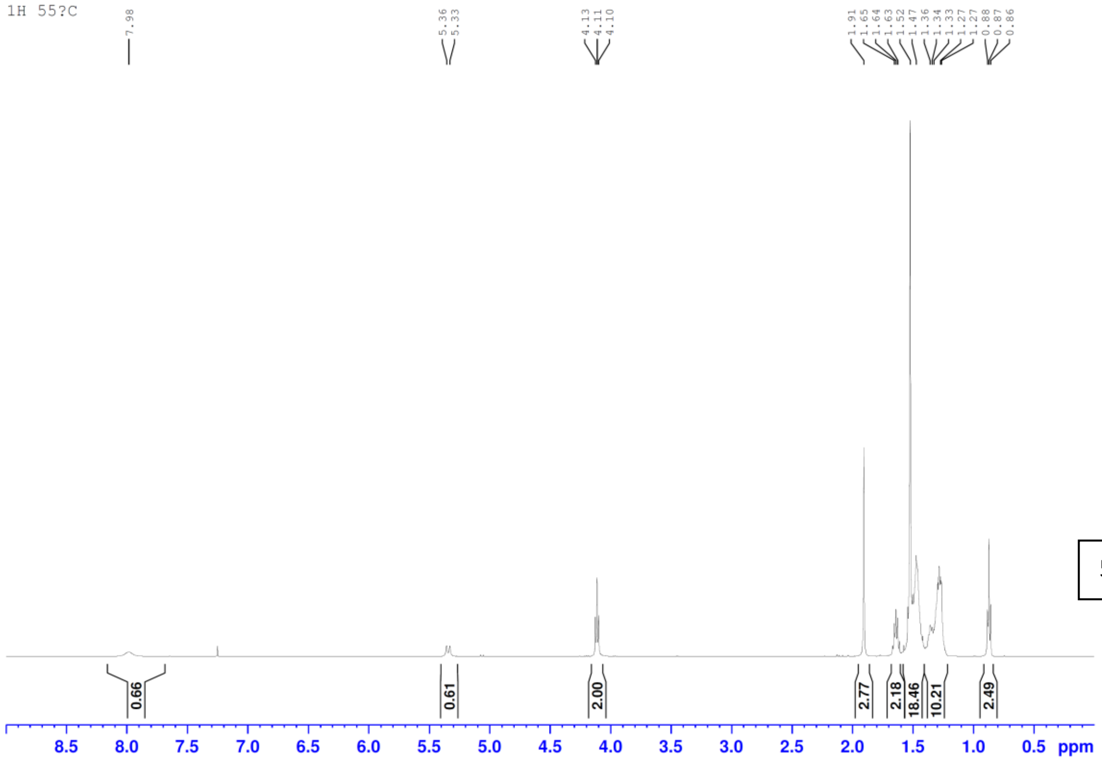

500 MHz/CDCl$_3$

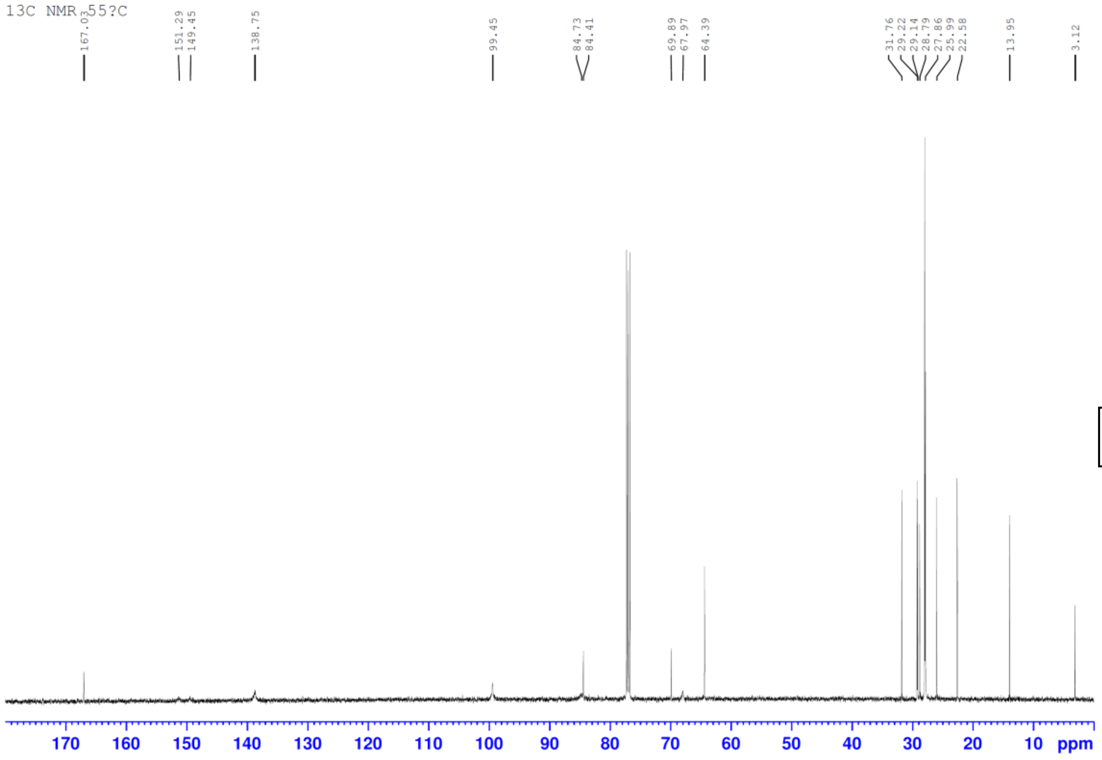

125 MHz/CDCl$_3$

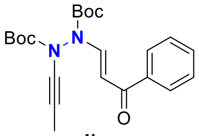
Boc
Boc
N
N
O

4k

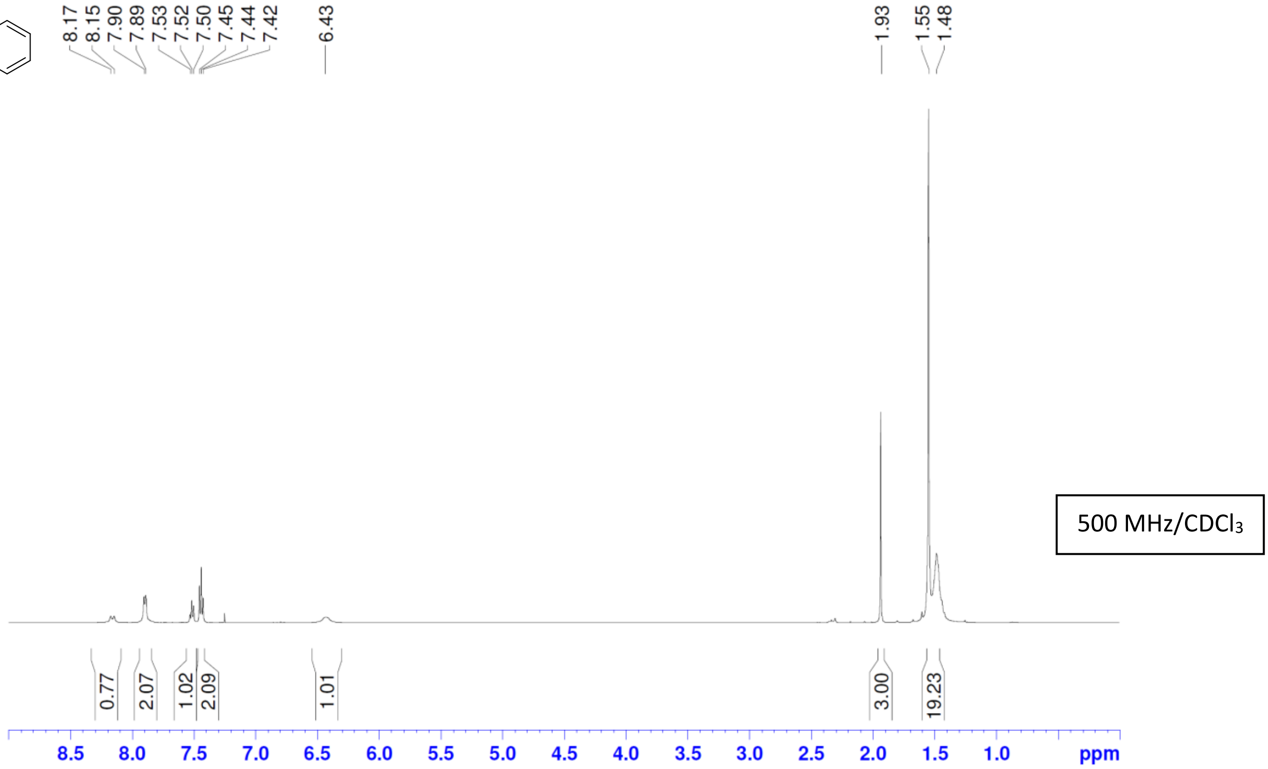
8.17
8.15
7.90
7.89
7.53
7.52
7.50
7.45
7.44
7.42
6.43
1.93
1.55
1.48
500 MHz/CDCl3
0.77
2.07
1.02
2.09
1.01
3.00
19.23
8.5 8.0 7.5 7.0 6.5 6.0 5.5 5.0 4.5 4.0 3.5 3.0 2.5 2.0 1.5 1.0 ppm

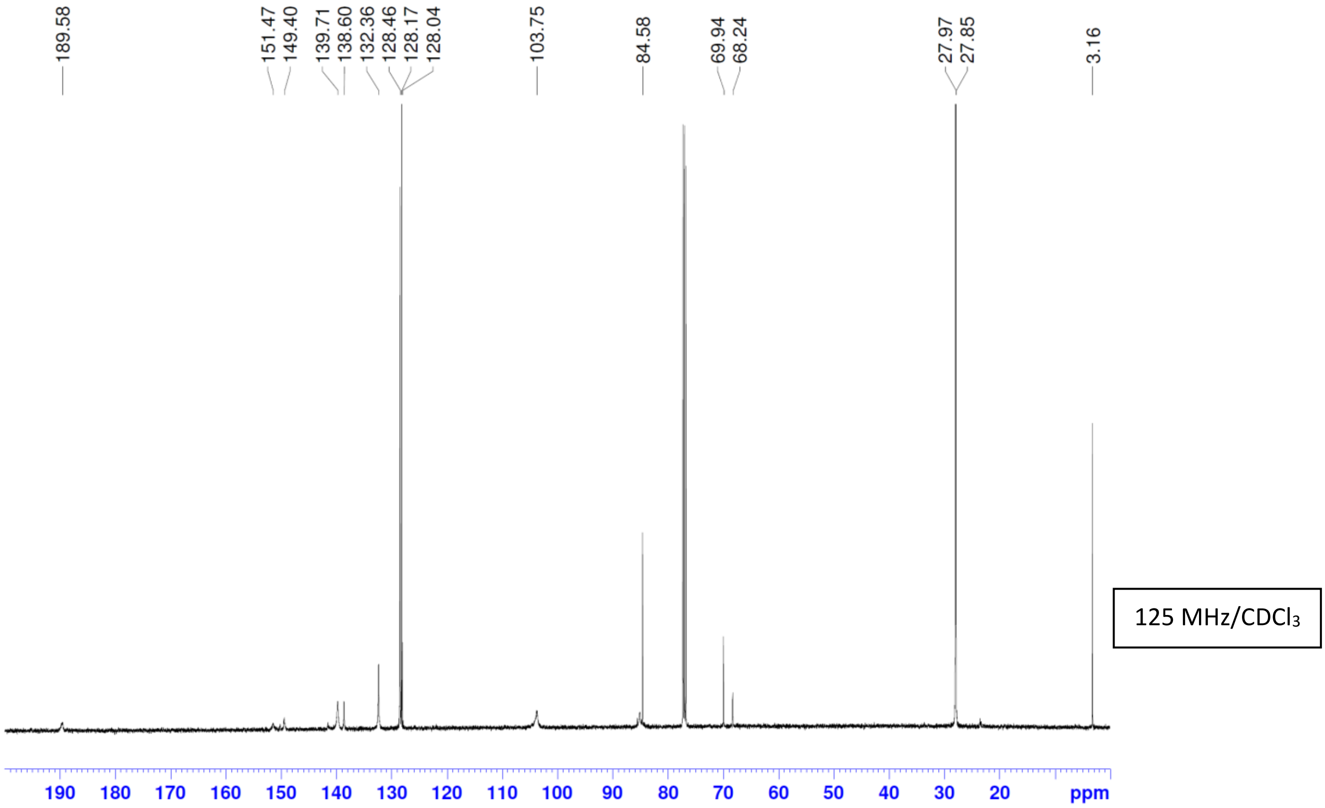
189.58
151.47
149.40
139.71
138.60
132.36
128.46
128.17
128.04
103.75
84.58
69.94
68.24
27.97
27.85
3.16
125 MHz/CDCl3
190 180 170 160 150 140 130 120 110 100 90 80 70 60 50 40 30 20 ppm

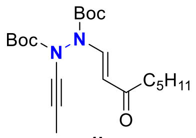
Boc
Boc
N
N
C5H11
O

4l

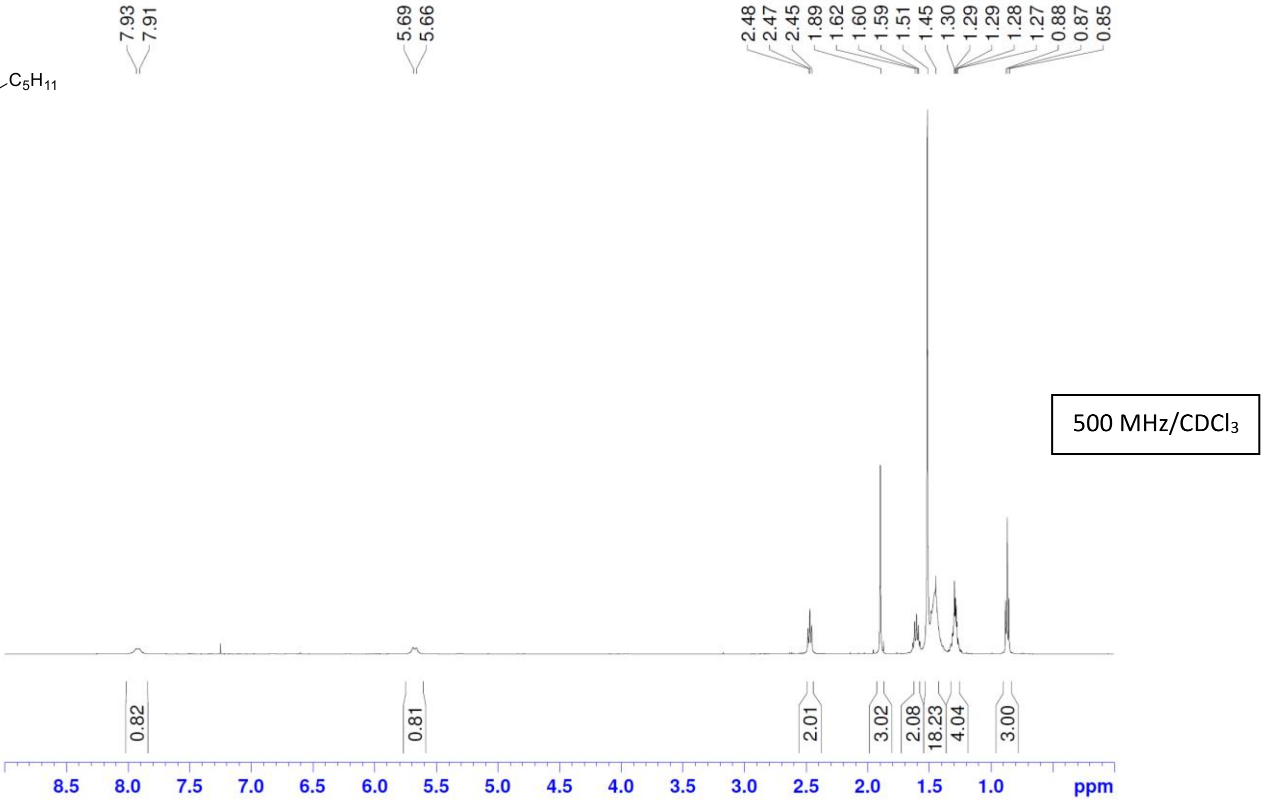
7.93
7.91
5.69
5.66
2.48
2.47
2.45
1.89
1.62
1.60
1.59
1.51
1.45
1.30
1.29
1.29
1.28
1.27
0.88
0.87
0.85
500 MHz/CDCl3
0.82
0.81
2.01
3.02
2.08
18.23
4.04
3.00
8.5
8.0
7.5
7.0
6.5
6.0
5.5
5.0
4.5
4.0
3.5
3.0
2.5
2.0
1.5
1.0
ppm

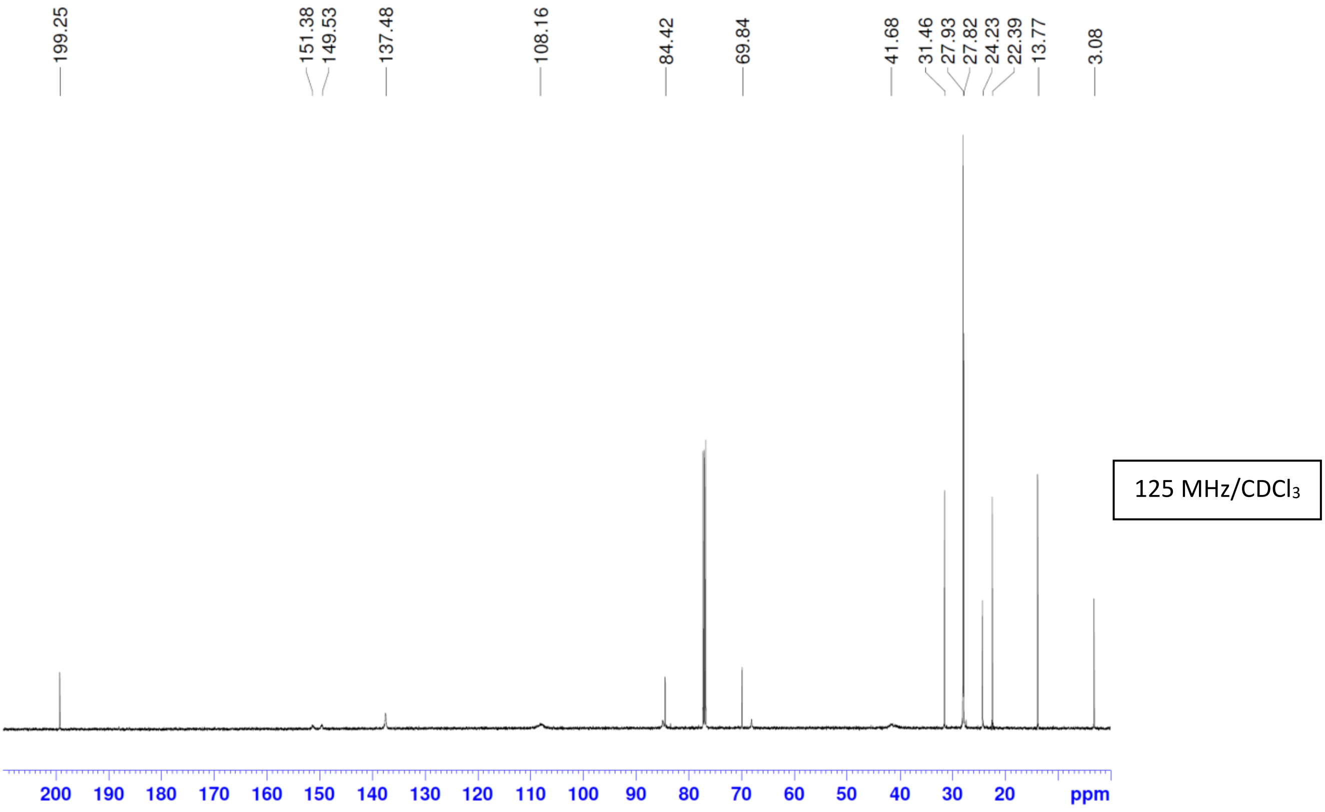
199.25
151.38
149.53
137.48
108.16
84.42
69.84
41.68
31.46
27.93
27.82
24.23
22.39
13.77
3.08
125 MHz/CDCl3
200
190
180
170
160
150
140
130
120
110
100
90
80
70
60
50
40
30
20
ppm

500 MHz/$CDCl_3$

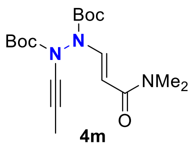

4m

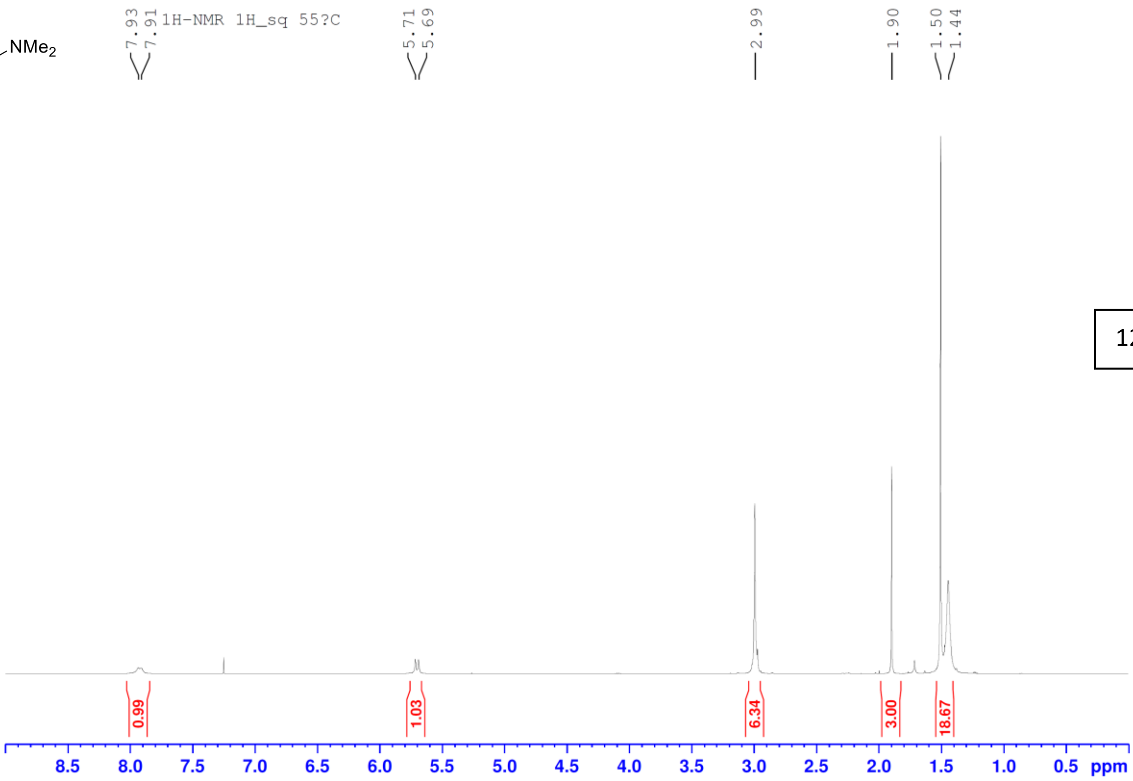

125 MHz/$CDCl_3$

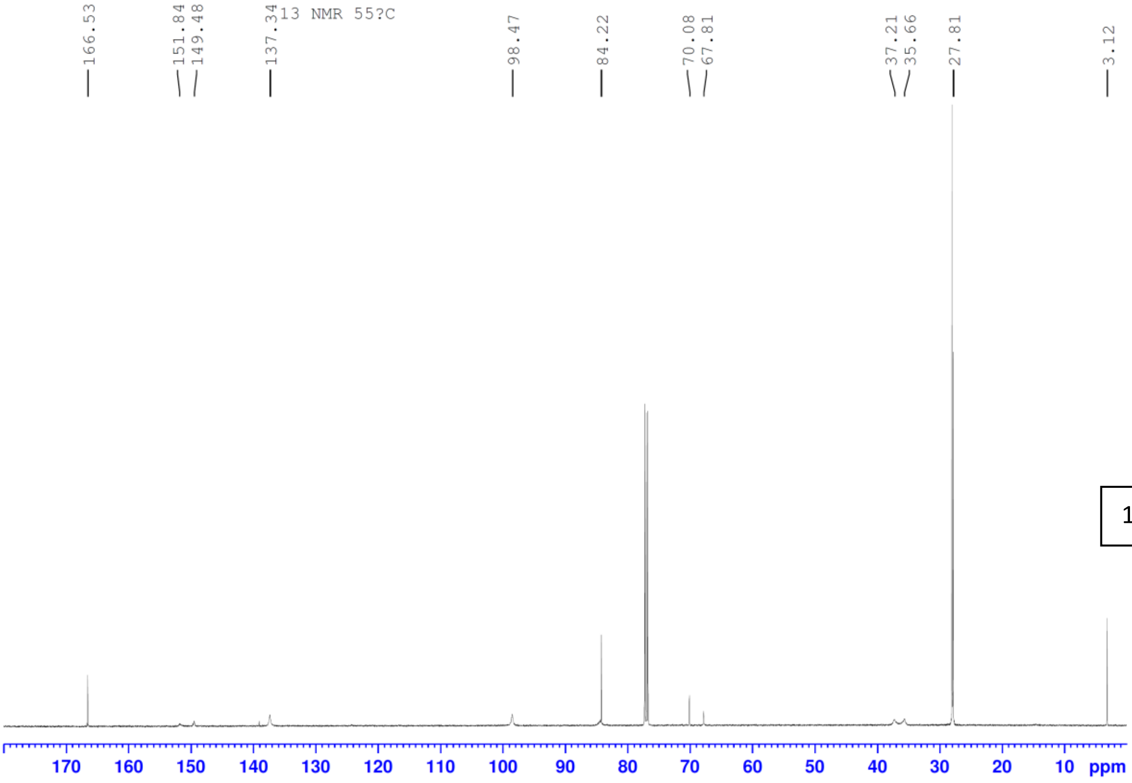

125 MHz/$CDCl_3$

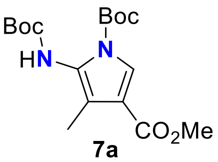
Boc
HN
Boc
N
CO2Me

7a

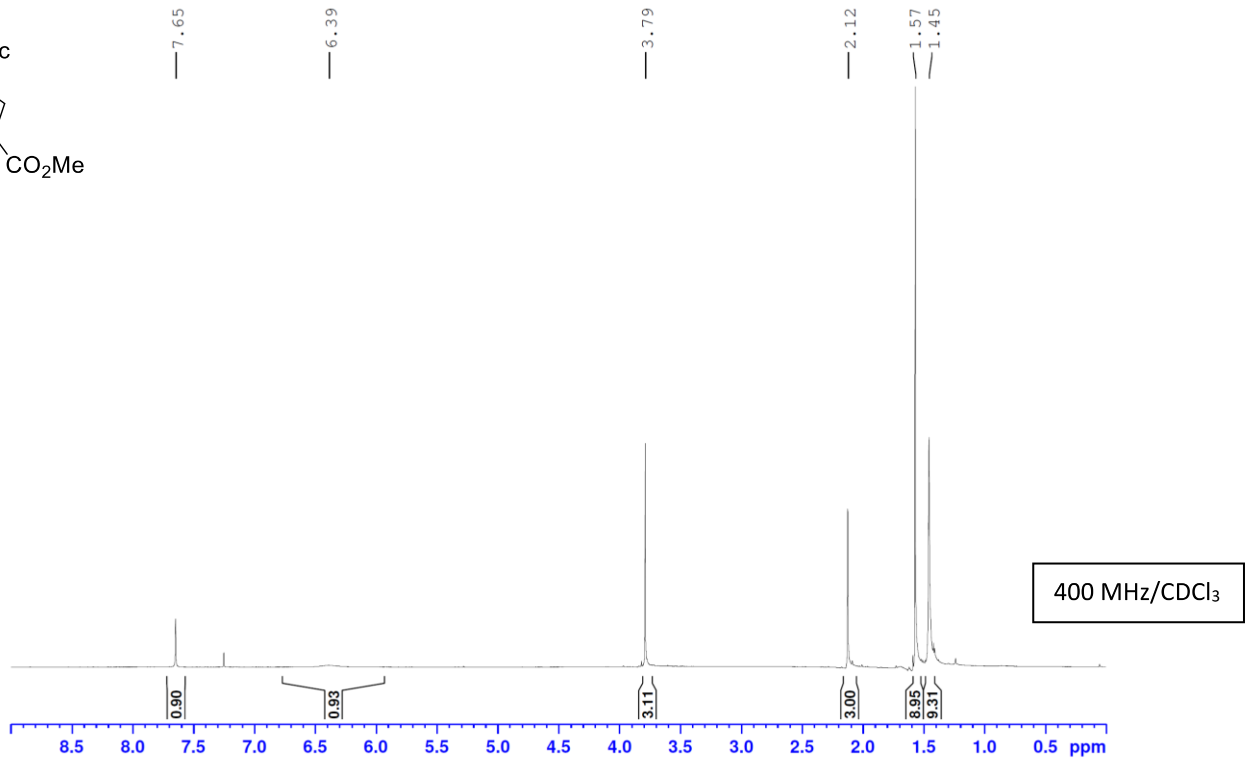
7.65
6.39
3.79
2.12
1.57
1.45
400 MHz/CDCl3
0.90
0.93
3.11
3.00
8.95
9.31
8.5
8.0
7.5
7.0
6.5
6.0
5.5
5.0
4.5
4.0
3.5
3.0
2.5
2.0
1.5
1.0
0.5
ppm

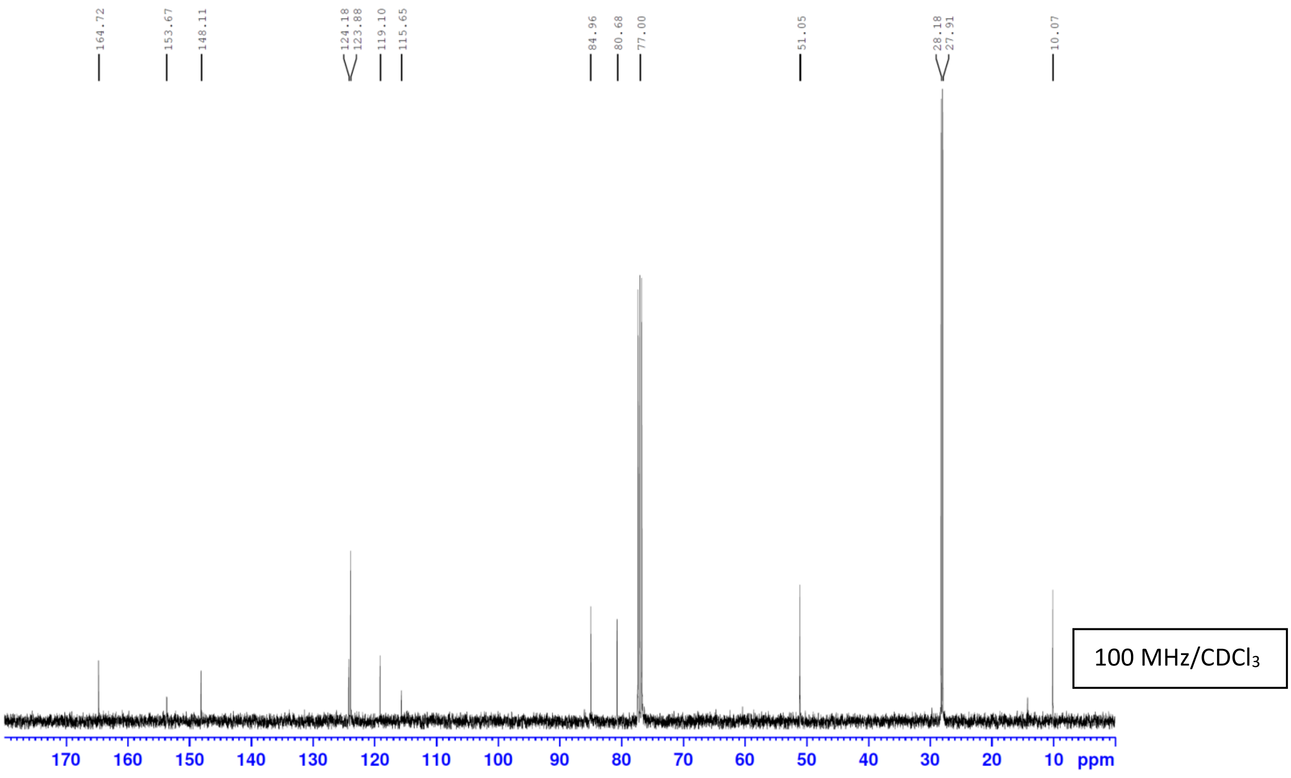
164.72
153.67
148.11
124.18
123.88
119.10
115.65
84.96
80.68
77.00
51.05
28.18
27.91
10.07
100 MHz/CDCl3
170
160
150
140
130
120
110
100
90
80
70
60
50
40
30
20
10
ppm

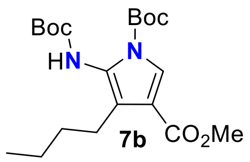

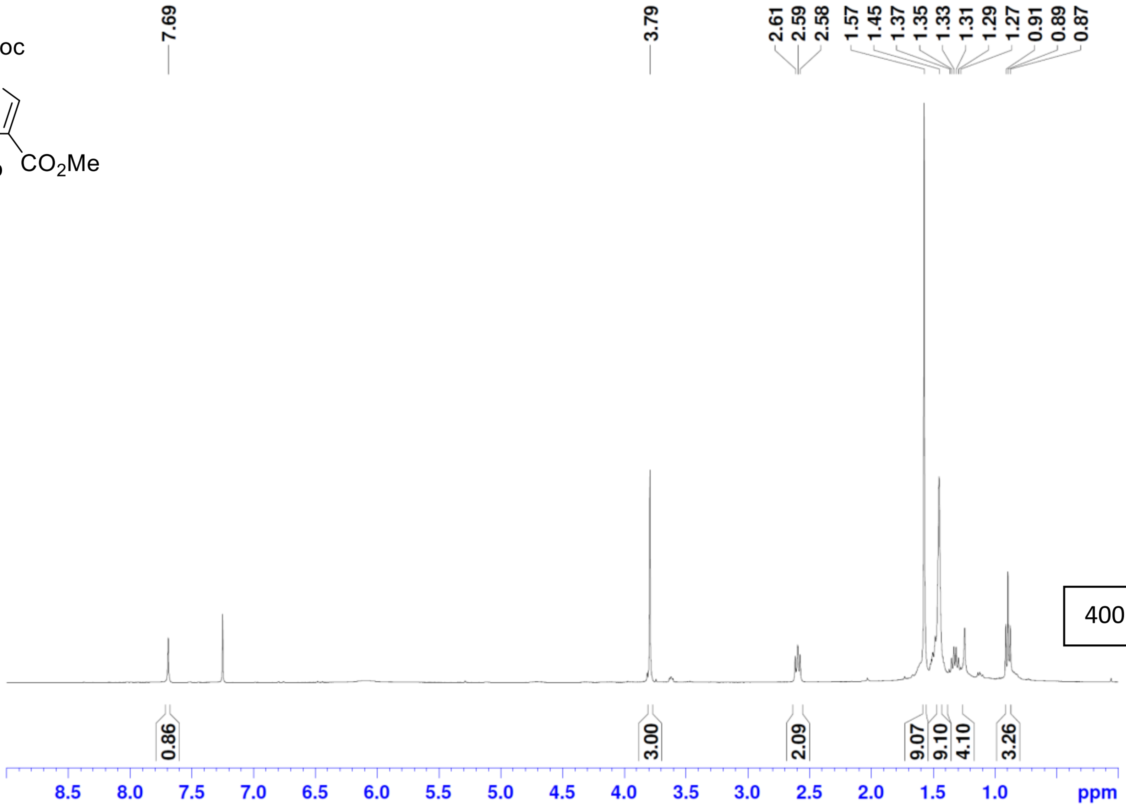

400 MHz/$CDCl_3$

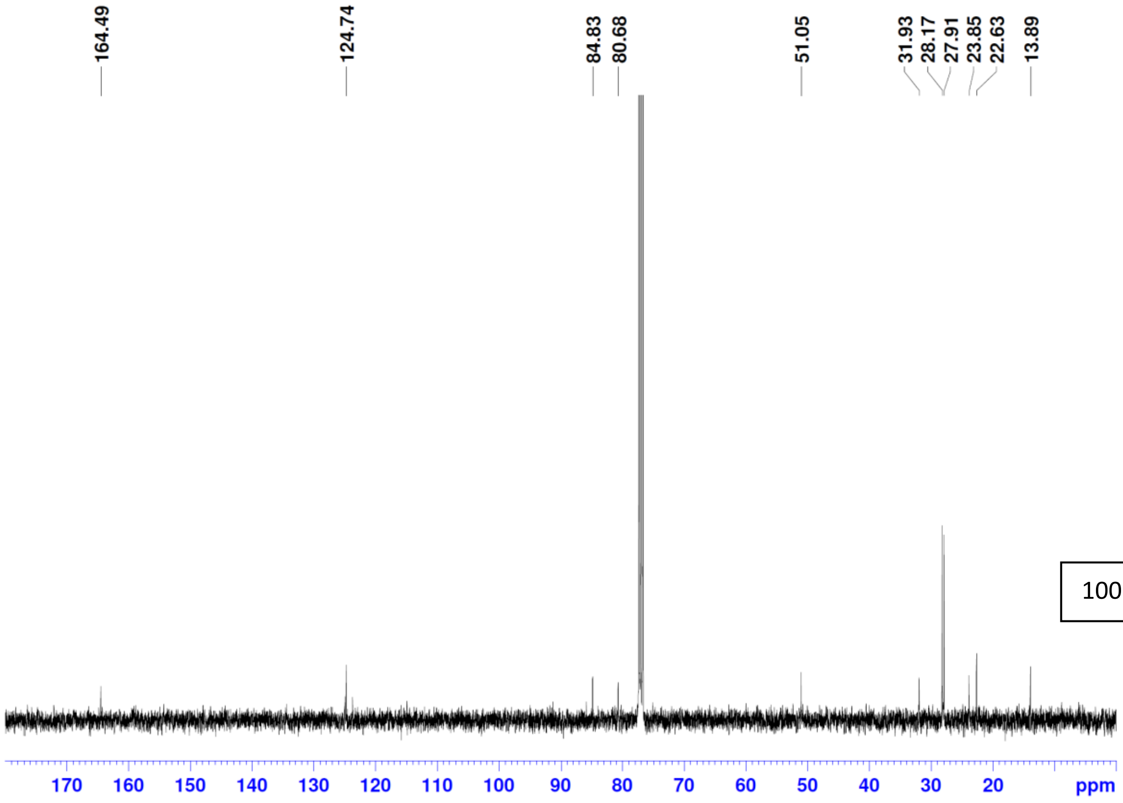

100 MHz/$CDCl_3$

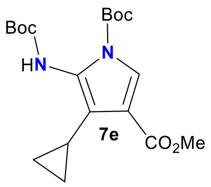
Boc
Boc
HN
N
7e
CO_2Me

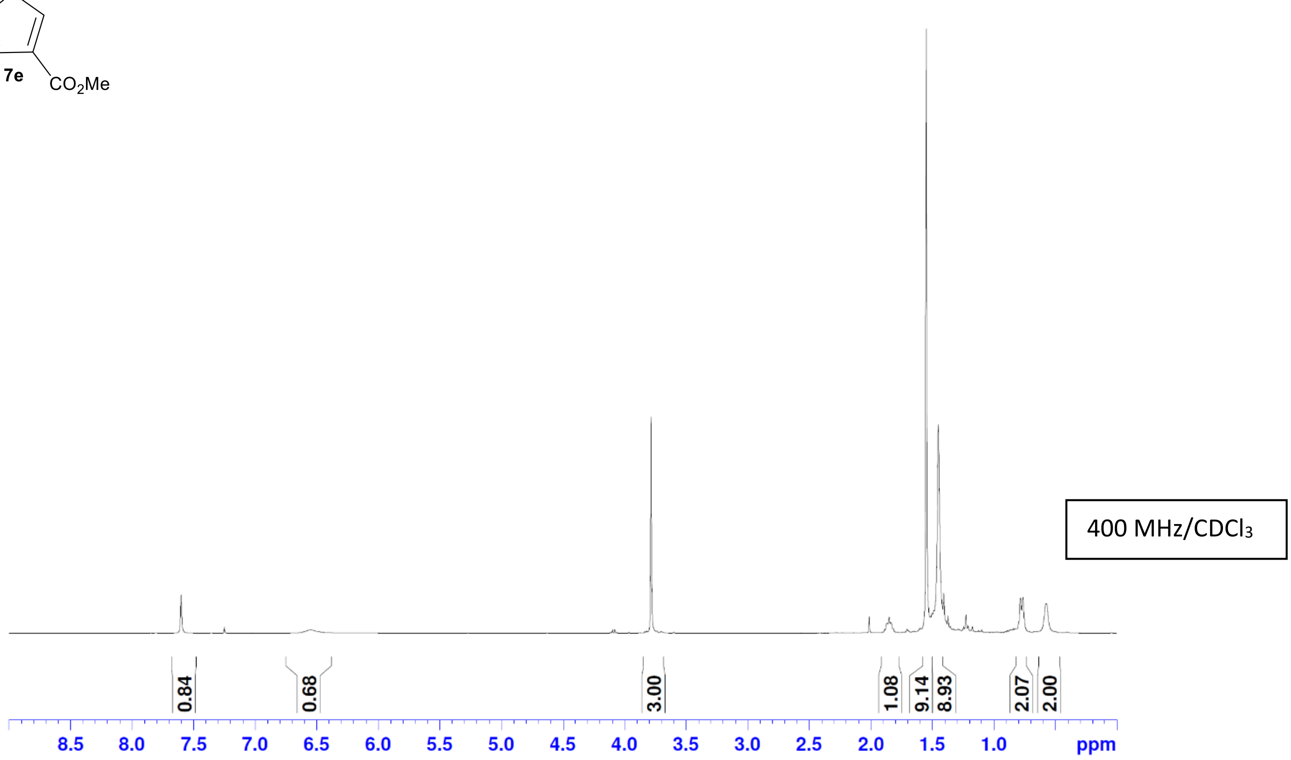
7.60
6.55
3.78
1.88
1.87
1.86
1.85
1.84
1.82
1.55
1.45
0.78
0.78
0.76
0.58
400 MHz/$CDCl_3$
0.84
0.68
3.00
1.08
9.14
8.93
2.07
2.00
8.5 8.0 7.5 7.0 6.5 6.0 5.5 5.0 4.5 4.0 3.5 3.0 2.5 2.0 1.5 1.0 ppm

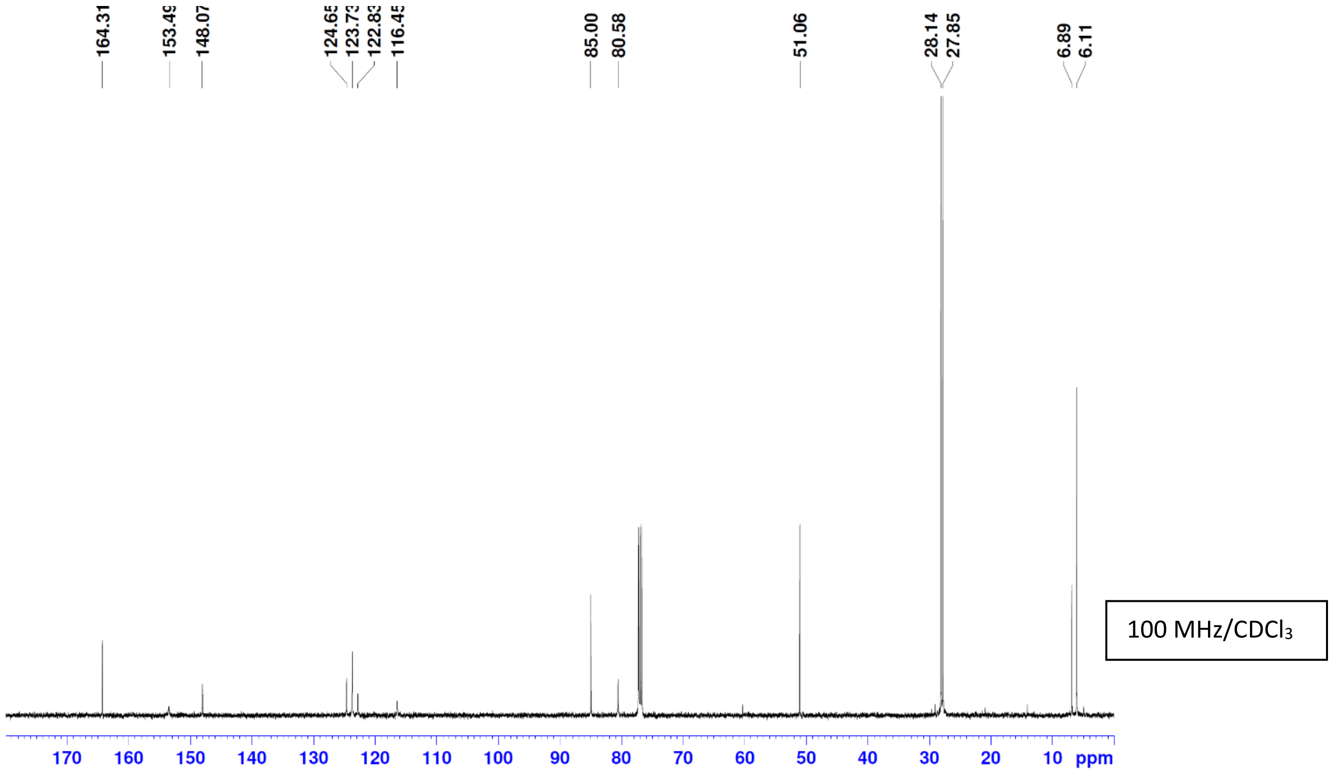
164.31
153.49
148.07
124.65
123.73
122.83
116.45
85.00
80.58
51.06
28.14
27.85
6.89
6.11
100 MHz/$CDCl_3$
170 160 150 140 130 120 110 100 90 80 70 60 50 40 30 20 10 ppm

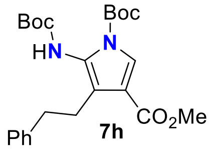
Boc
HN
Boc
N
CO2Me
Ph
7h

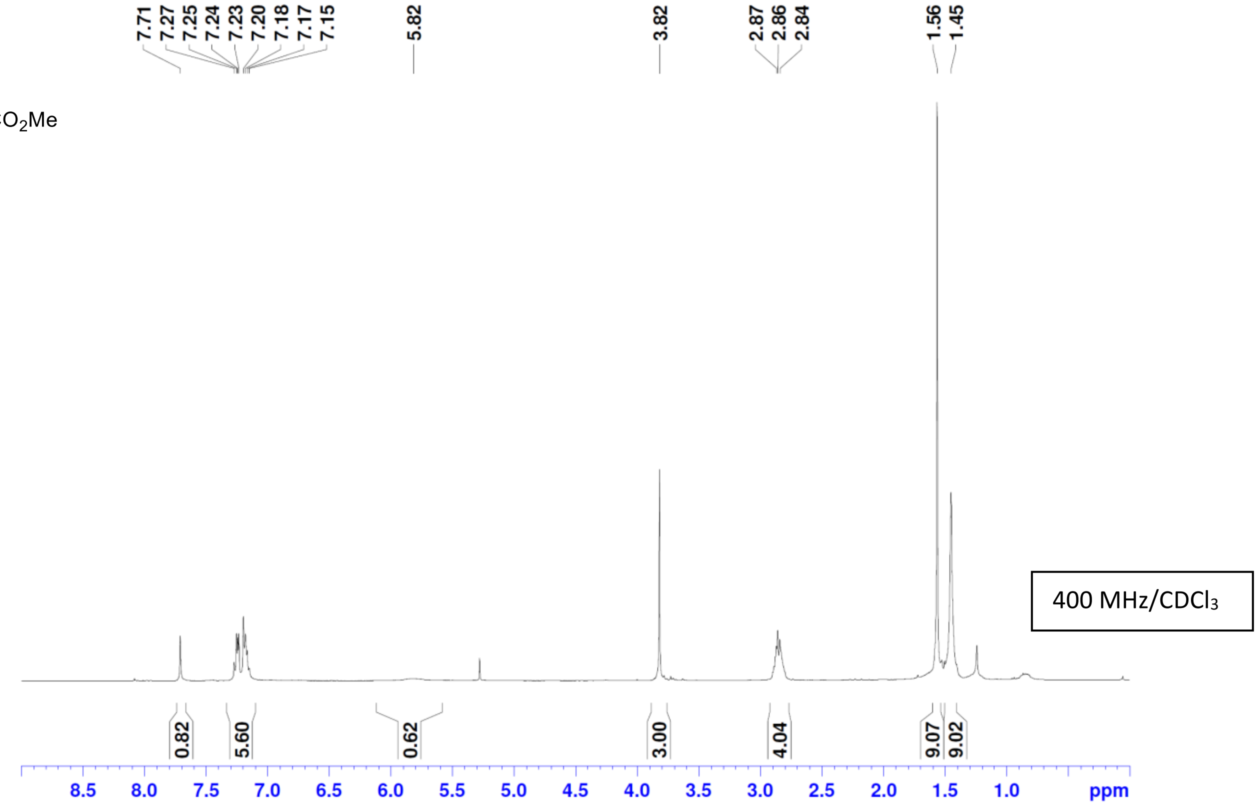
400 MHz/CDCl3
ppm

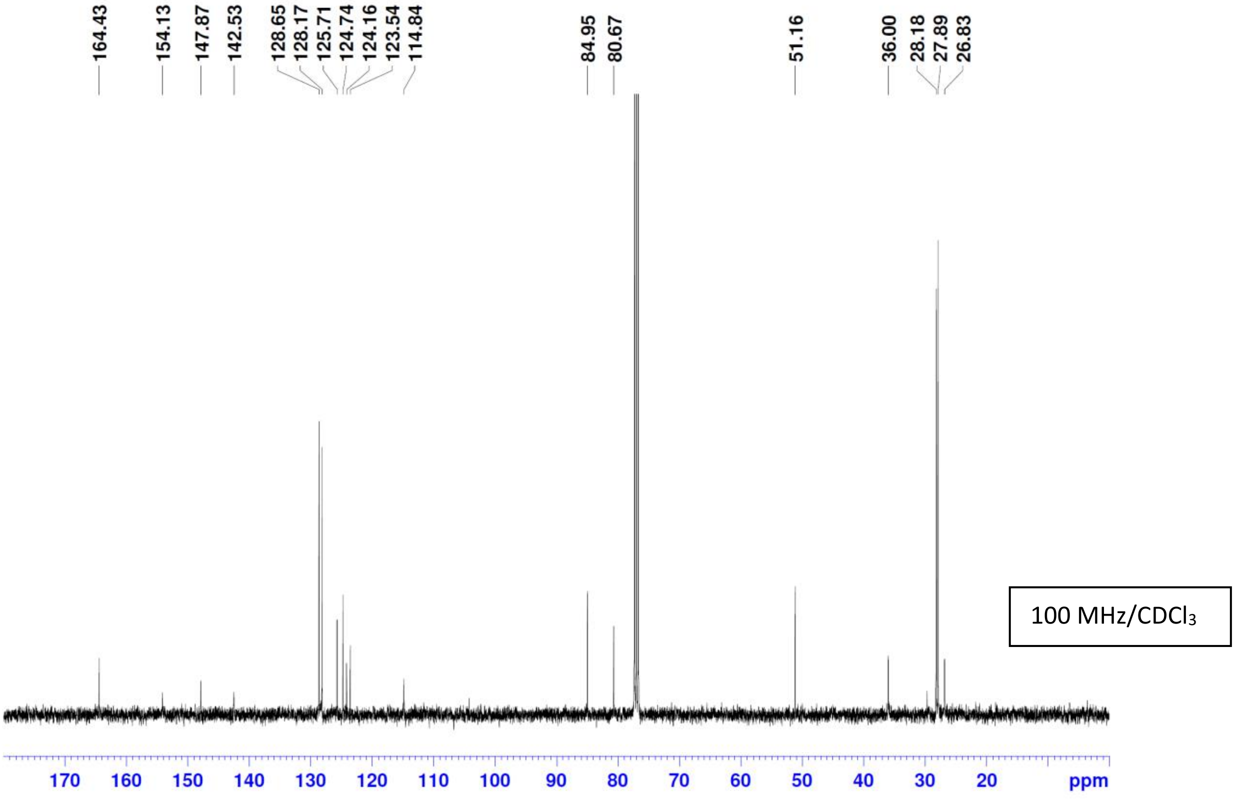
100 MHz/CDCl3
ppm

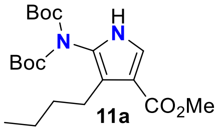
Boc
Boc
N
H
N
CO2Me
11a

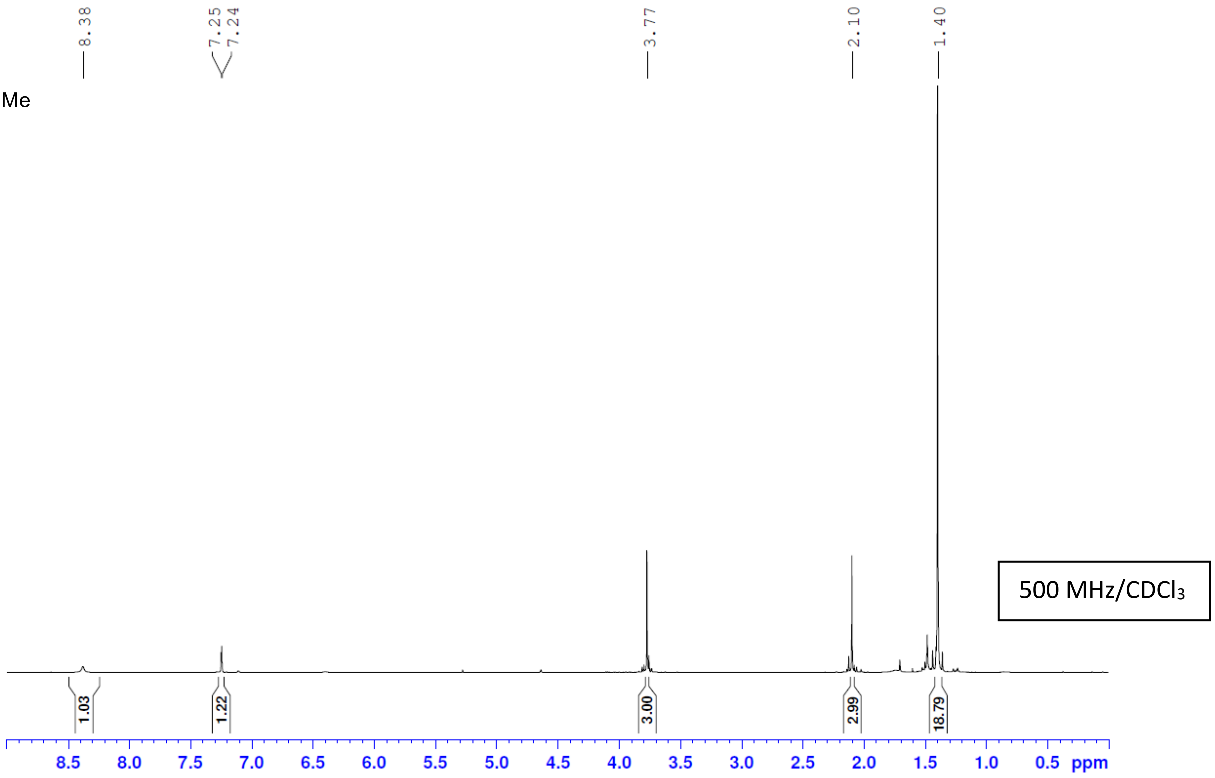
8.38
7.25
7.24
3.77
2.10
1.40
500 MHz/CDCl3
1.03
1.22
3.00
2.99
18.79
8.5 8.0 7.5 7.0 6.5 6.0 5.5 5.0 4.5 4.0 3.5 3.0 2.5 2.0 1.5 1.0 0.5 ppm

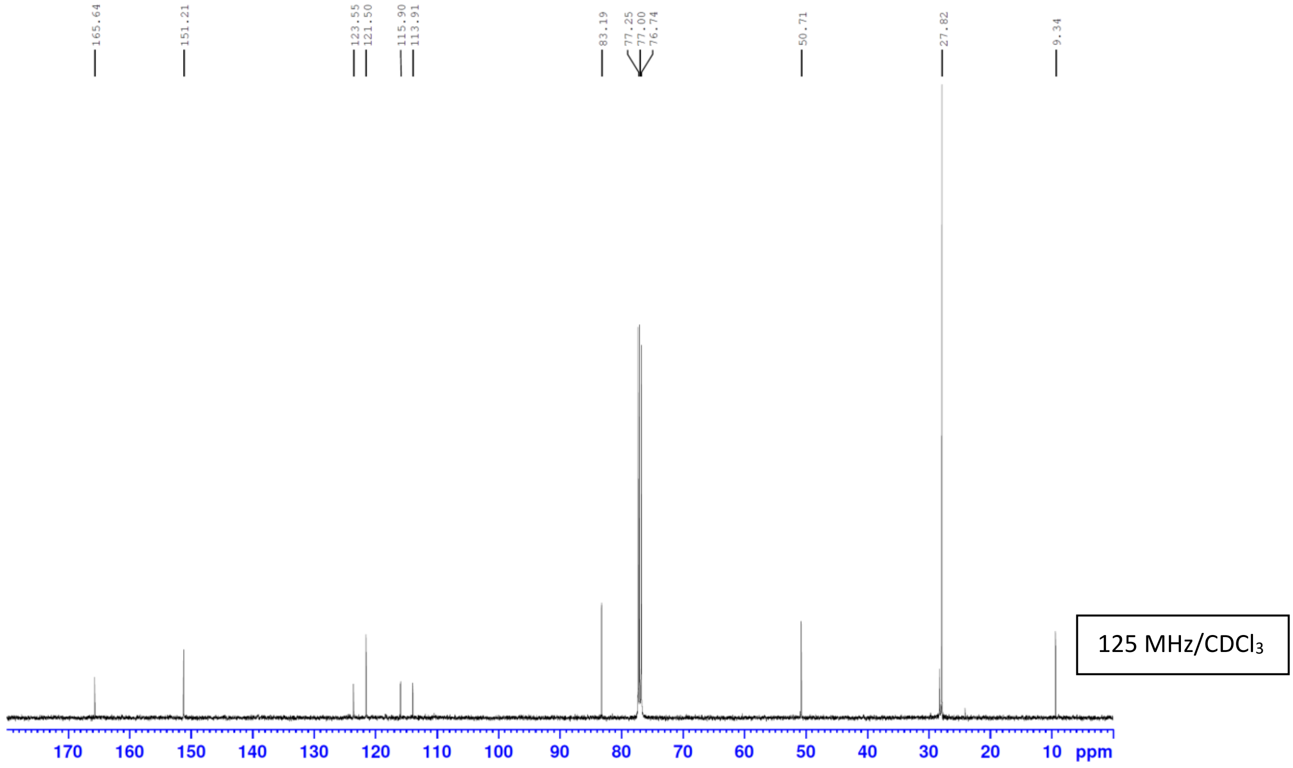
165.64
151.21
123.55
121.50
115.90
113.91
83.19
77.25
77.00
76.74
50.71
27.82
9.34
125 MHz/CDCl3
170 160 150 140 130 120 110 100 90 80 70 60 50 40 30 20 10 ppm

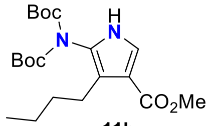
Boc
Boc
N
H
N
CO2Me
11b

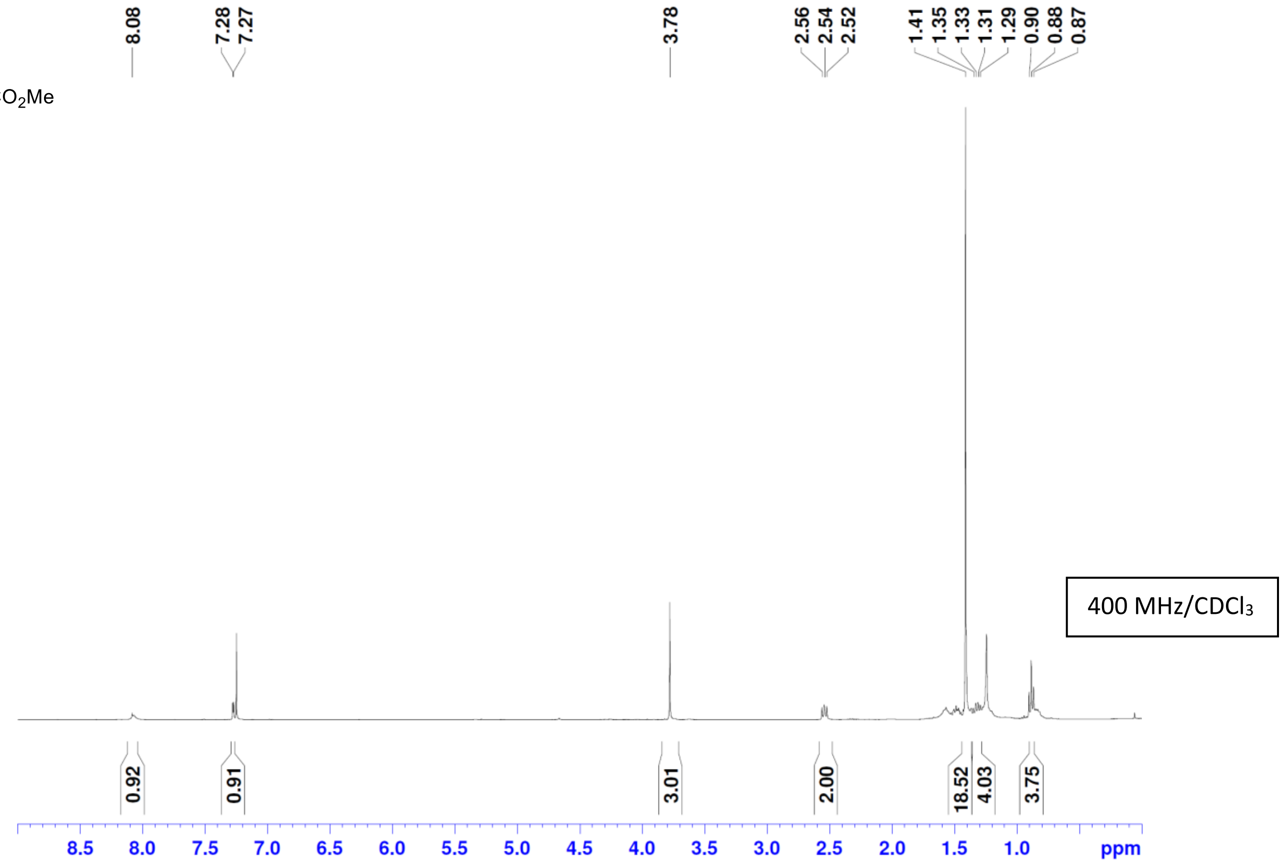
8.08
7.28
7.27
3.78
2.56
2.54
2.52
1.41
1.35
1.33
1.31
1.29
0.90
0.88
0.87
400 MHz/CDCl3
0.92
0.91
3.01
2.00
18.52
4.03
3.75
8.5 8.0 7.5 7.0 6.5 6.0 5.5 5.0 4.5 4.0 3.5 3.0 2.5 2.0 1.5 1.0 ppm

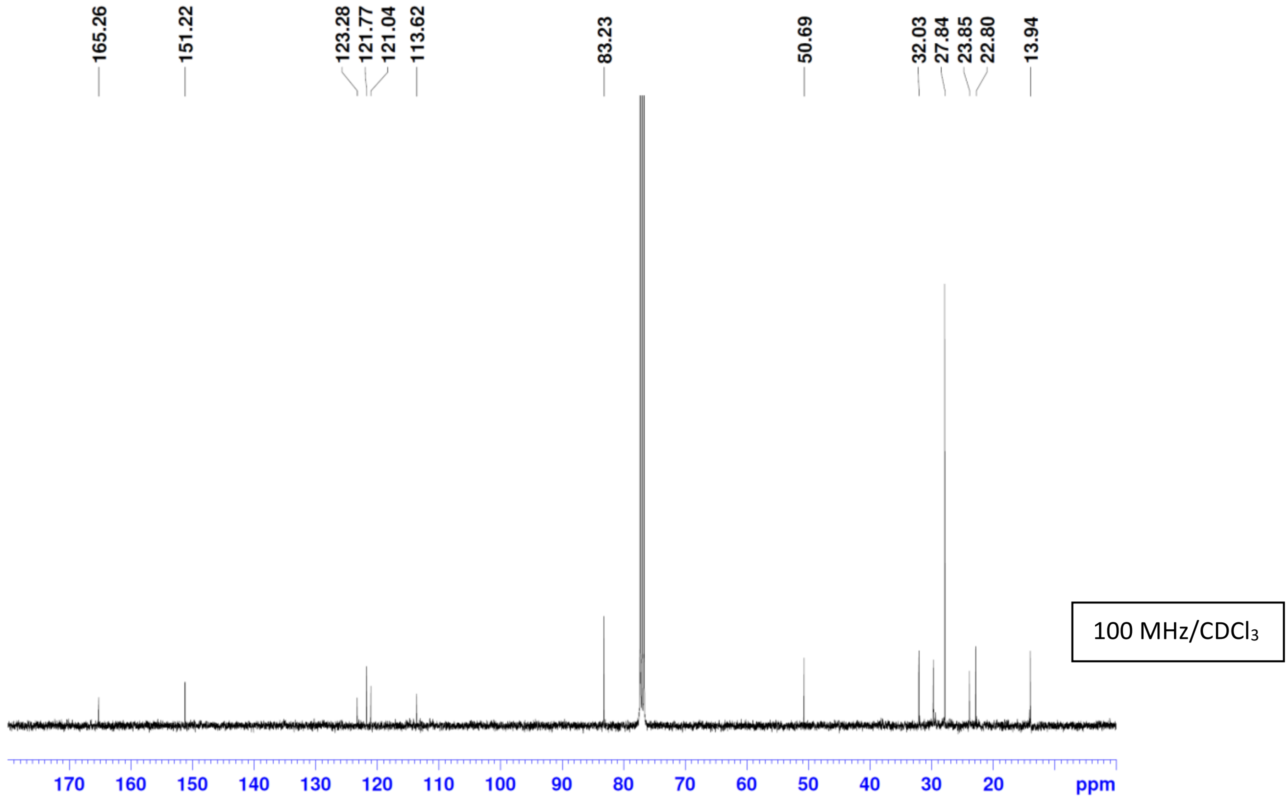
165.26
151.22
123.28
121.77
121.04
113.62
83.23
50.69
32.03
27.84
23.85
22.80
13.94
100 MHz/CDCl3
170 160 150 140 130 120 110 100 90 80 70 60 50 40 30 20 ppm

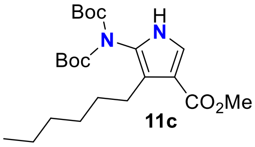
Boc
Boc
N
H
N
CO2Me

11c

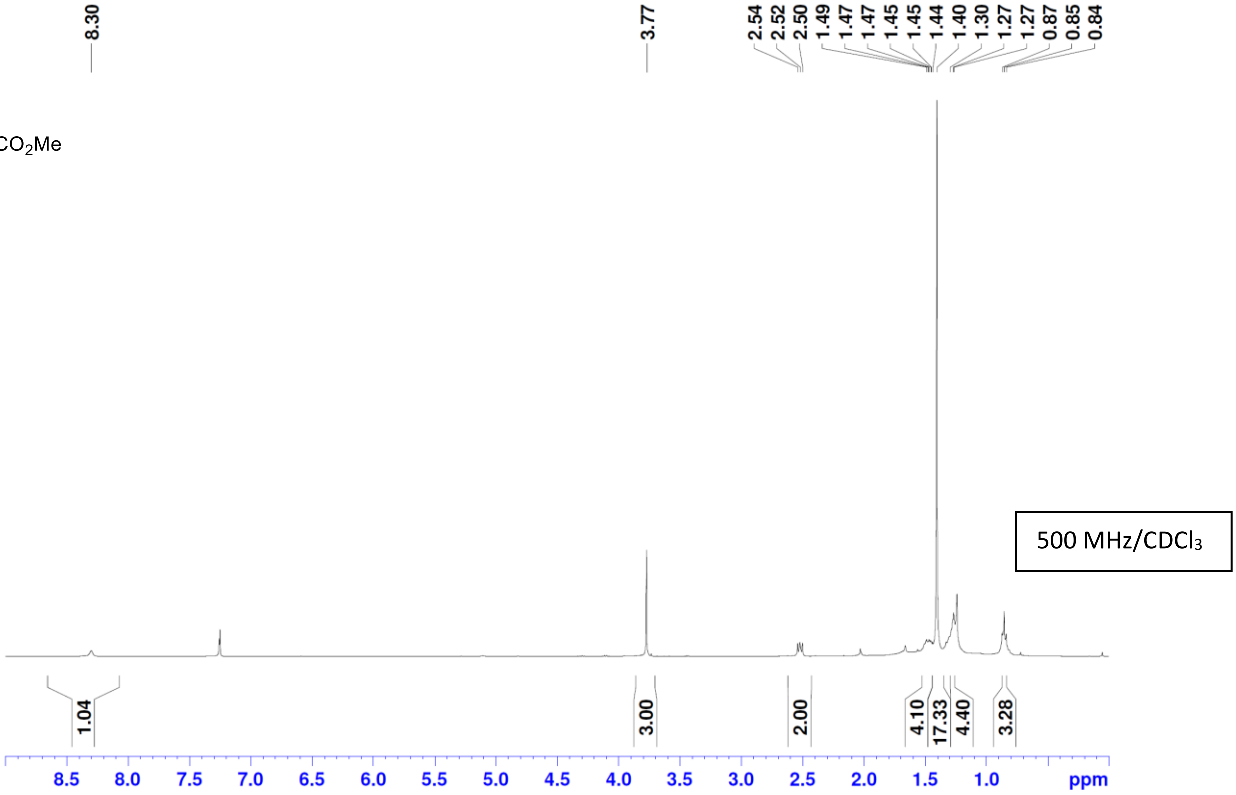
8.30
3.77
2.54
2.52
2.50
1.49
1.47
1.47
1.45
1.45
1.44
1.40
1.30
1.27
1.27
0.87
0.85
0.84
500 MHz/CDCl3
1.04
3.00
2.00
4.10
17.33
4.40
3.28
8.5 8.0 7.5 7.0 6.5 6.0 5.5 5.0 4.5 4.0 3.5 3.0 2.5 2.0 1.5 1.0 ppm

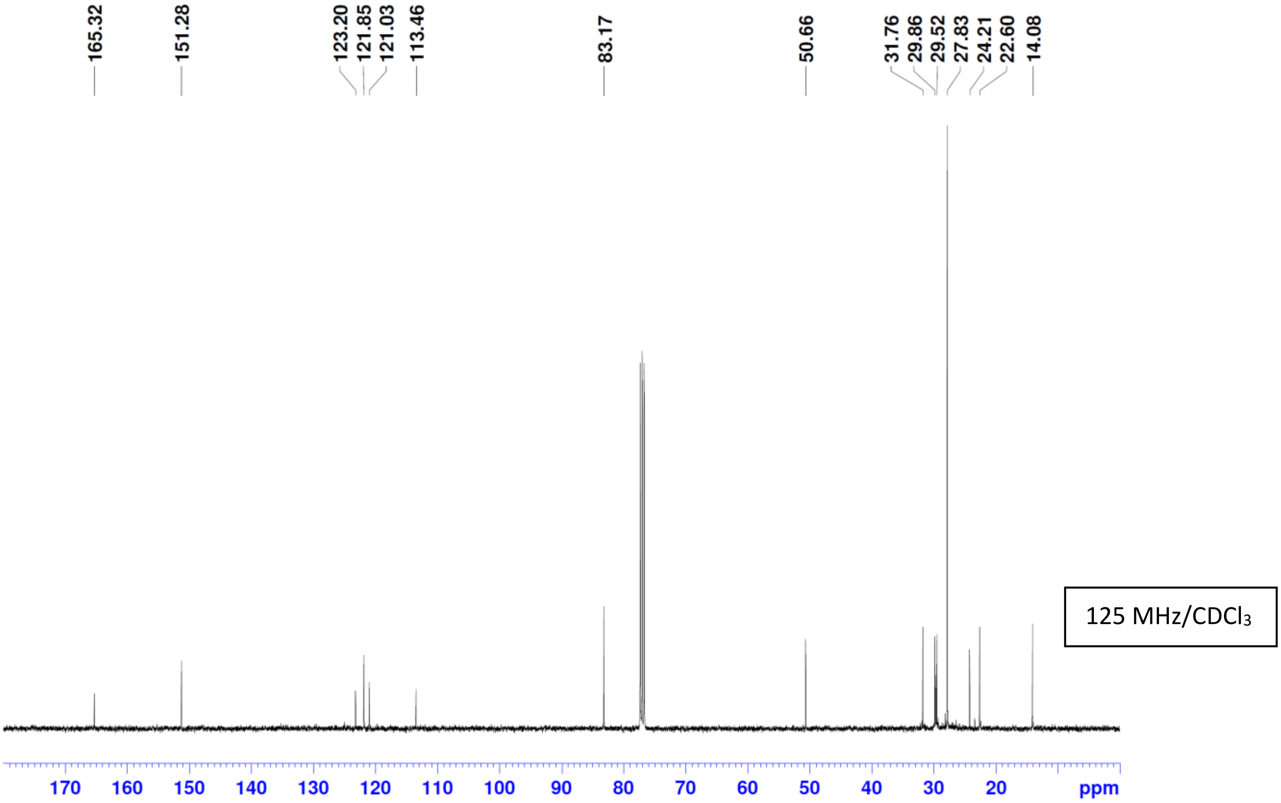
165.32
151.28
123.20
121.85
121.03
113.46
83.17
50.66
31.76
29.86
29.52
27.83
24.21
22.60
14.08
125 MHz/CDCl3
170 160 150 140 130 120 110 100 90 80 70 60 50 40 30 20 ppm

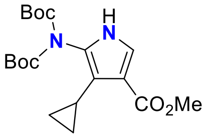
Boc
Boc
N
H
N
CO2Me

11e

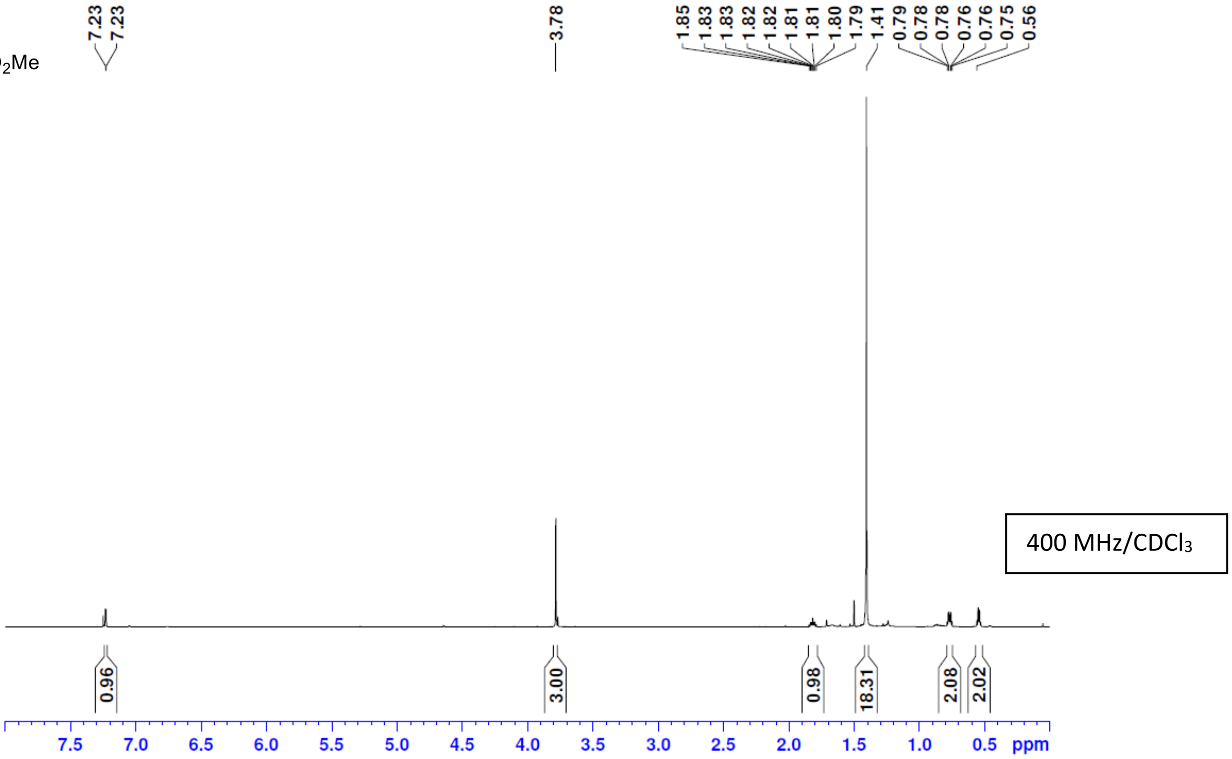
7.23
7.23
3.78
1.85
1.83
1.83
1.82
1.82
1.81
1.81
1.80
1.79
1.41
0.79
0.78
0.78
0.76
0.76
0.75
0.56
400 MHz/CDCl3
0.96
3.00
0.98
18.31
2.08
2.02
7.5 7.0 6.5 6.0 5.5 5.0 4.5 4.0 3.5 3.0 2.5 2.0 1.5 1.0 0.5 ppm

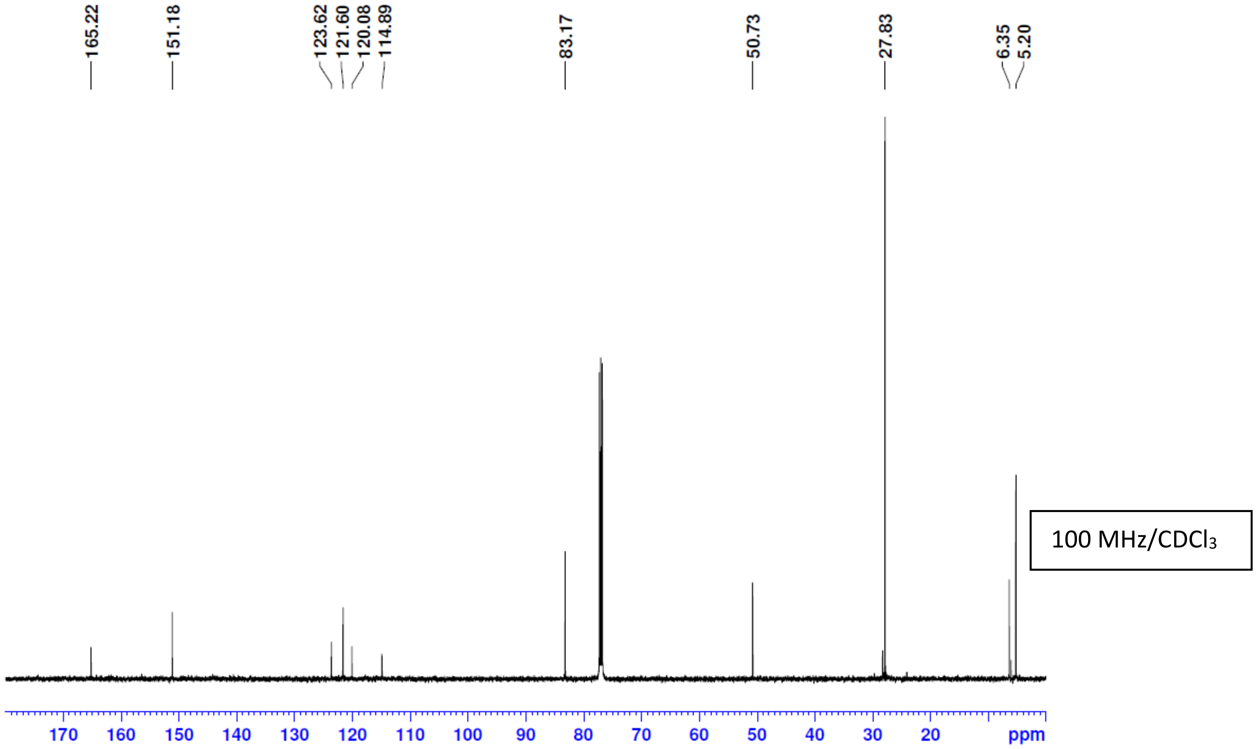
165.22
151.18
123.62
121.60
120.08
114.89
83.17
50.73
27.83
6.35
5.20
100 MHz/CDCl3
170 160 150 140 130 120 110 100 90 80 70 60 50 40 30 20 ppm

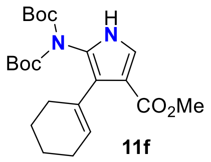
Boc
Boc
N
H
N
CO_2Me

11f

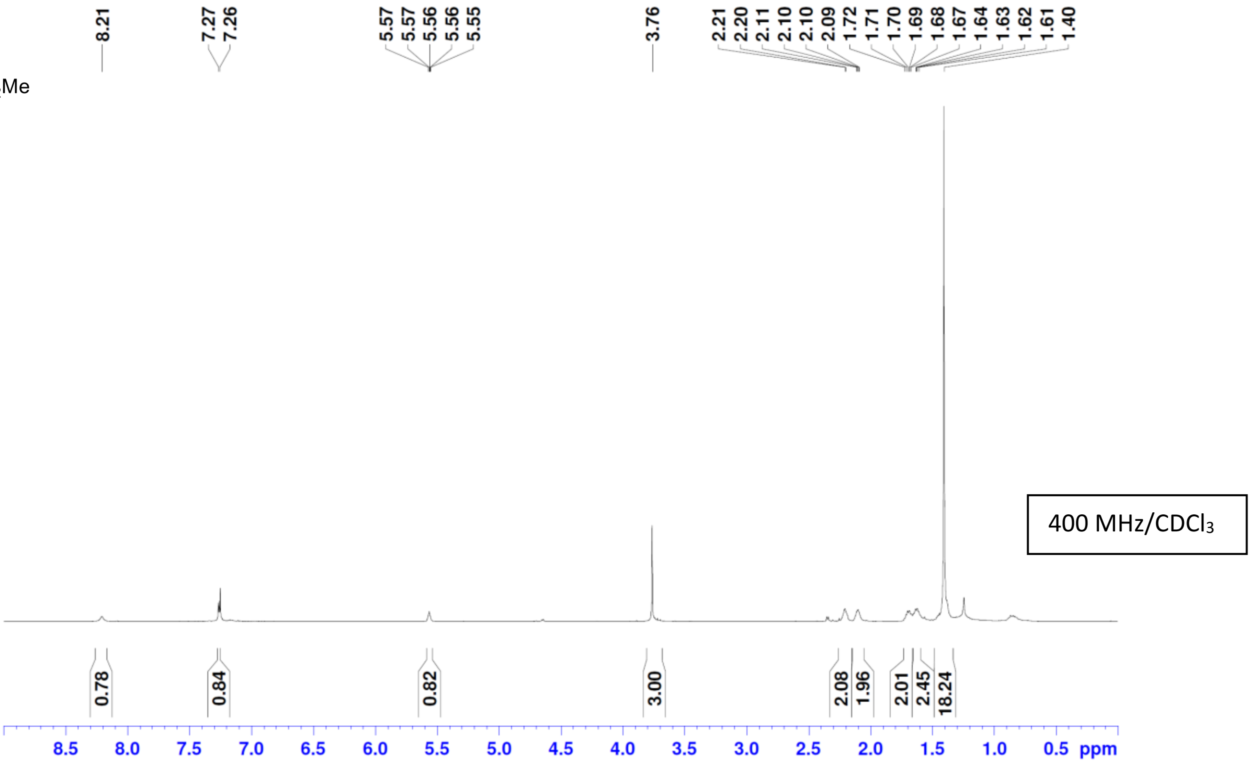
8.21
7.27
7.26
5.57
5.57
5.56
5.56
5.55
3.76
2.21
2.20
2.11
2.10
2.10
2.09
1.72
1.71
1.70
1.69
1.68
1.67
1.64
1.63
1.62
1.61
1.40
400 MHz/$CDCl_3$
0.78
0.84
0.82
3.00
2.08
1.96
2.01
2.45
18.24
8.5 8.0 7.5 7.0 6.5 6.0 5.5 5.0 4.5 4.0 3.5 3.0 2.5 2.0 1.5 1.0 0.5 ppm

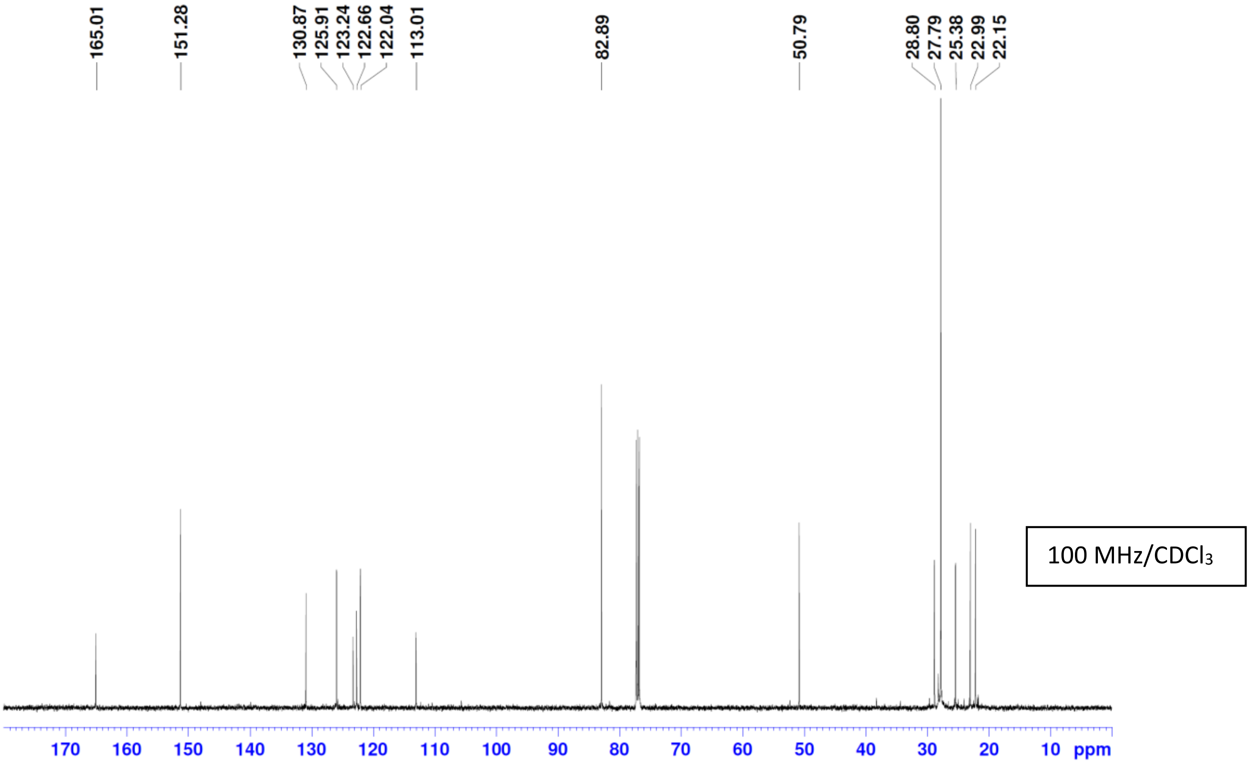
165.01
151.28
130.87
125.91
123.24
122.66
122.04
113.01
82.89
50.79
28.80
27.79
25.38
22.99
22.15
100 MHz/$CDCl_3$
170 160 150 140 130 120 110 100 90 80 70 60 50 40 30 20 10 ppm

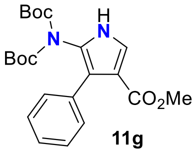
Boc
Boc
N
H
N
CO2Me

11g

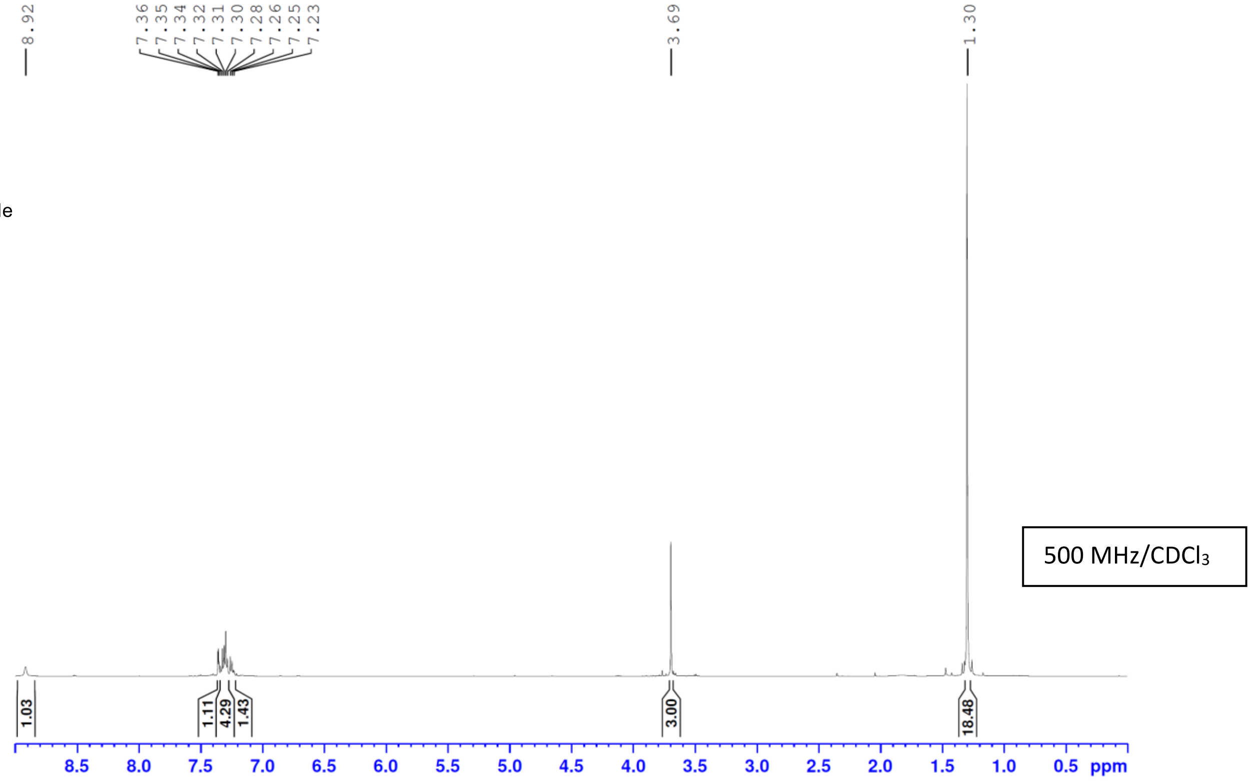
8.92
7.36
7.35
7.34
7.32
7.31
7.30
7.28
7.26
7.25
7.23
3.69
1.30
500 MHz/CDCl3
1.03
1.11
4.29
1.43
3.00
18.48
8.5 8.0 7.5 7.0 6.5 6.0 5.5 5.0 4.5 4.0 3.5 3.0 2.5 2.0 1.5 1.0 0.5 ppm

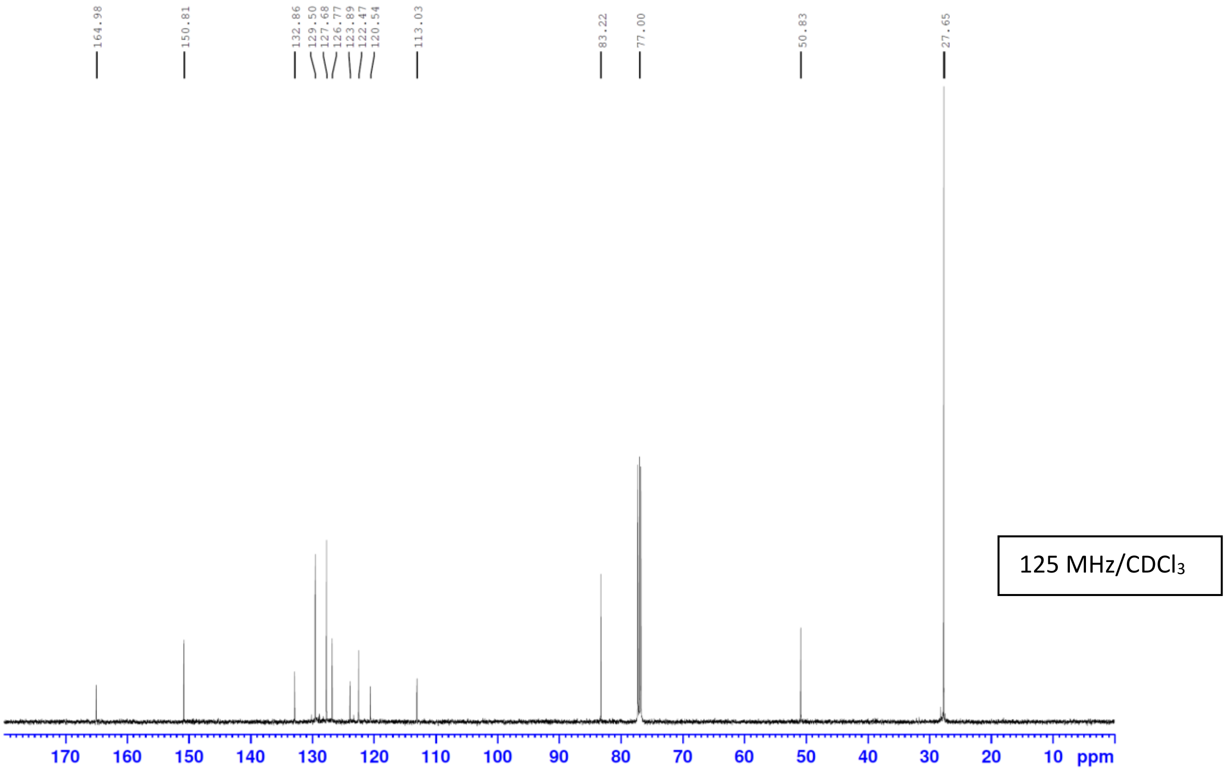
164.98
150.81
132.86
129.50
127.68
126.77
123.89
122.47
120.54
113.03
83.22
77.00
50.83
27.65
125 MHz/CDCl3
170 160 150 140 130 120 110 100 90 80 70 60 50 40 30 20 10 ppm

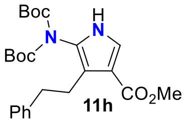
Boc
Boc
N
H
N
CO2Me
Ph
11h

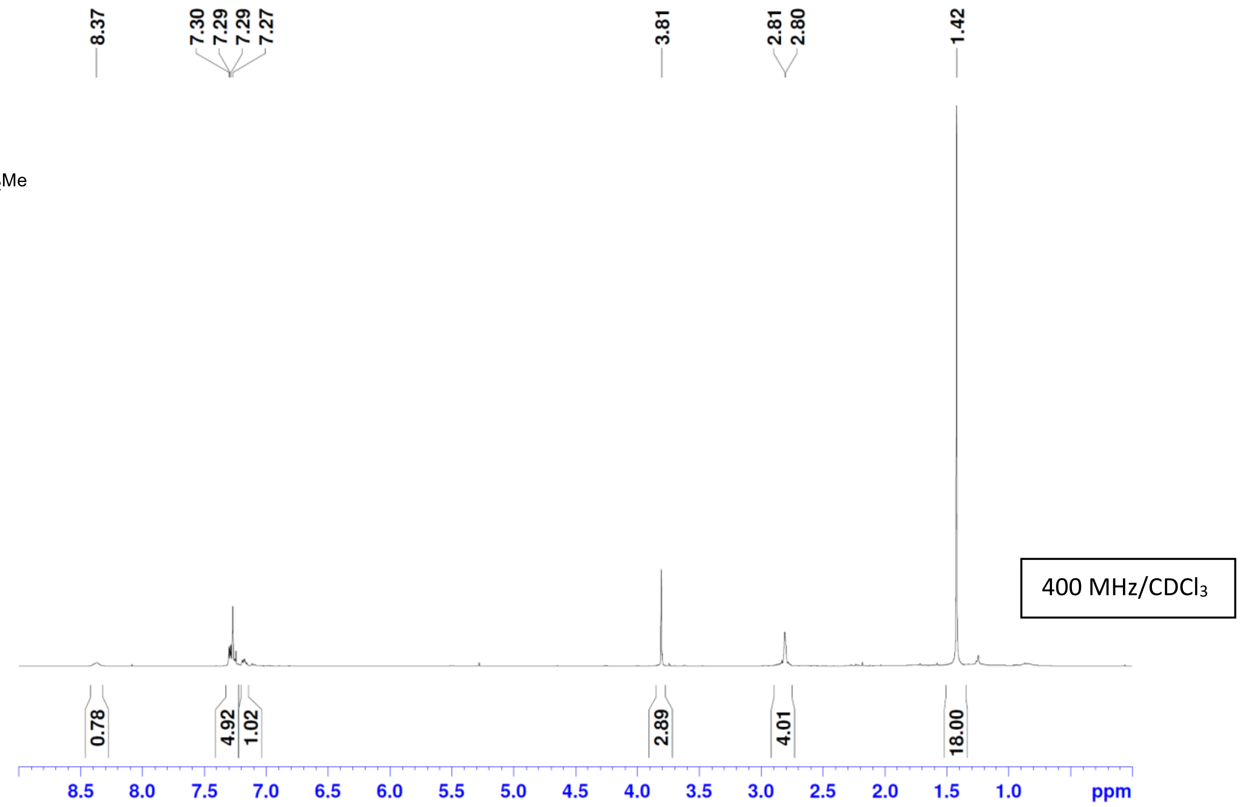
8.37
7.30
7.29
7.29
7.27
3.81
2.81
2.80
1.42
400 MHz/CDCl3
0.78
4.92
1.02
2.89
4.01
18.00
8.5 8.0 7.5 7.0 6.5 6.0 5.5 5.0 4.5 4.0 3.5 3.0 2.5 2.0 1.5 1.0 ppm

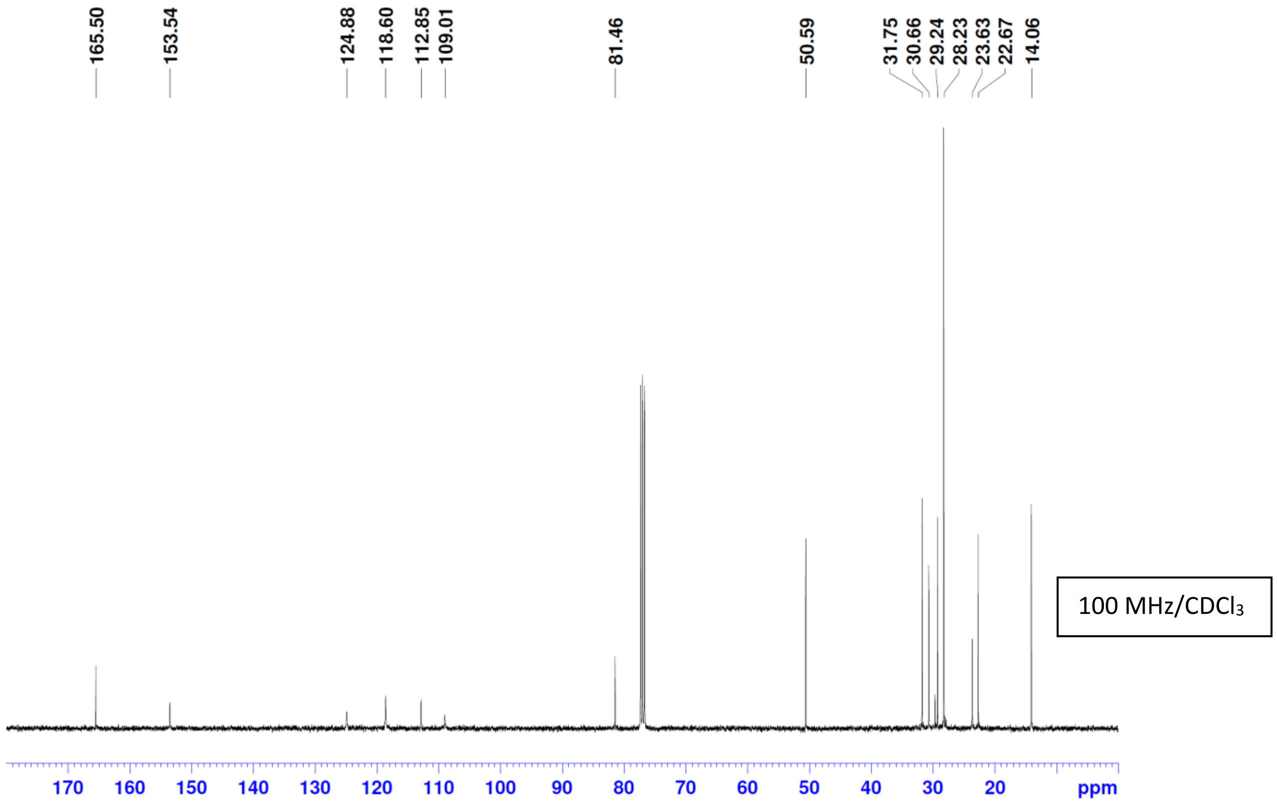
165.50
153.54
124.88
118.60
112.85
109.01
81.46
50.59
31.75
30.66
29.24
28.23
23.63
22.67
14.06
100 MHz/CDCl3
170 160 150 140 130 120 110 100 90 80 70 60 50 40 30 20 ppm

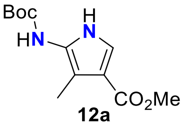
Boc
HN
H
N
CO2Me

12a

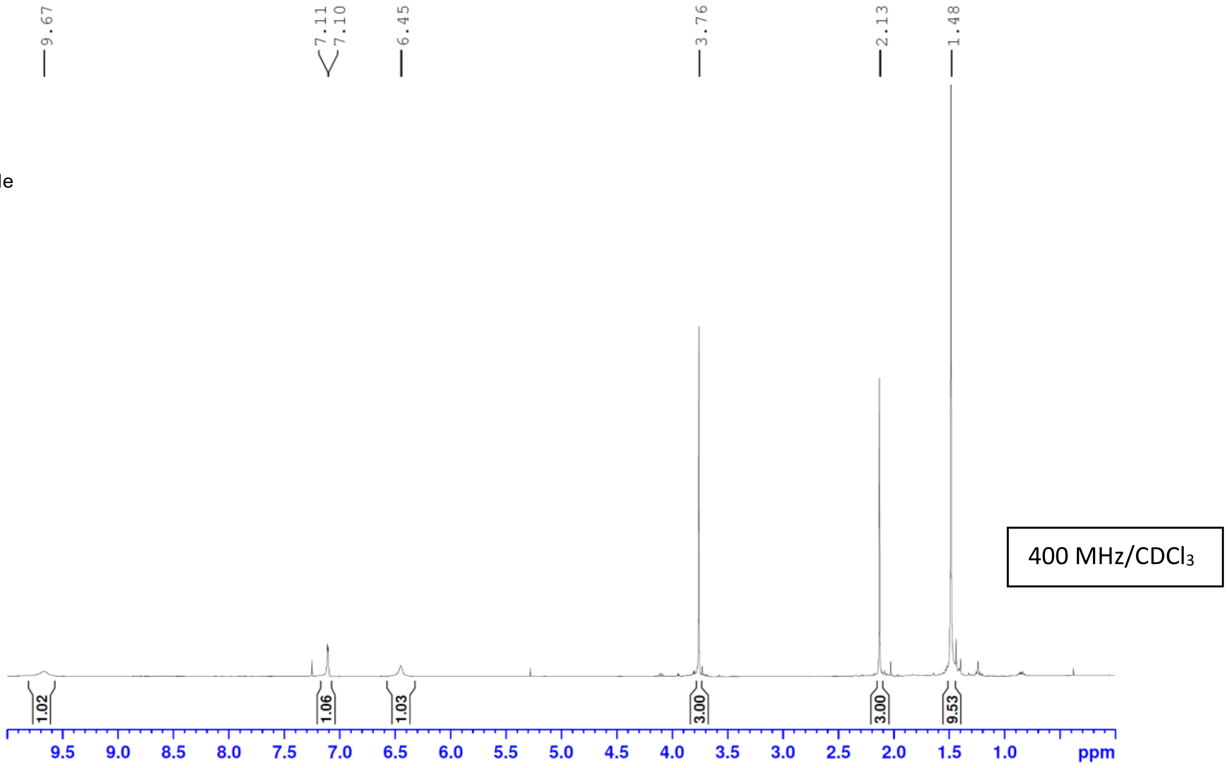
9.67
7.11
7.10
6.45
3.76
2.13
1.48
400 MHz/CDCl3
1.02
1.06
1.03
3.00
3.00
9.53
ppm

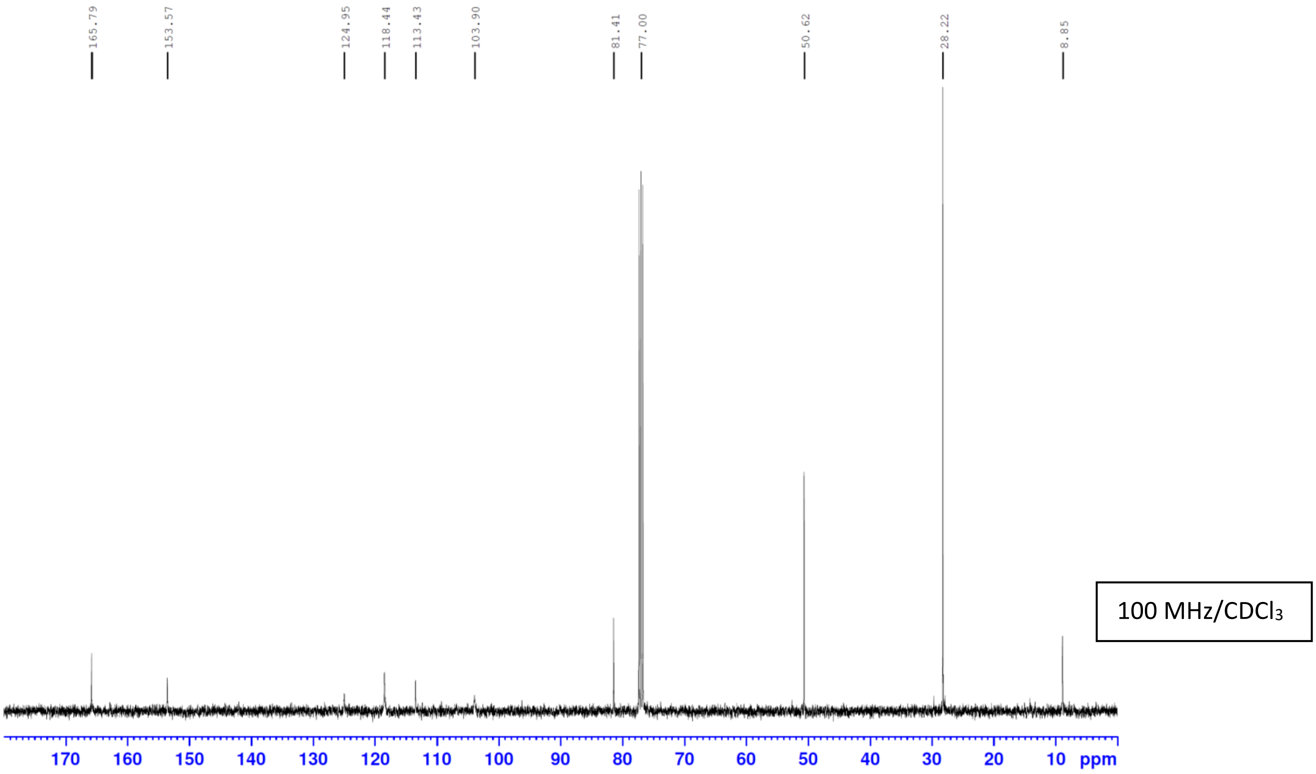
165.79
153.57
124.95
118.44
113.43
103.90
81.41
77.00
50.62
28.22
8.85
100 MHz/CDCl3
ppm

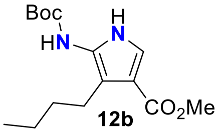
Boc
HN
H
N
CO2Me
12b

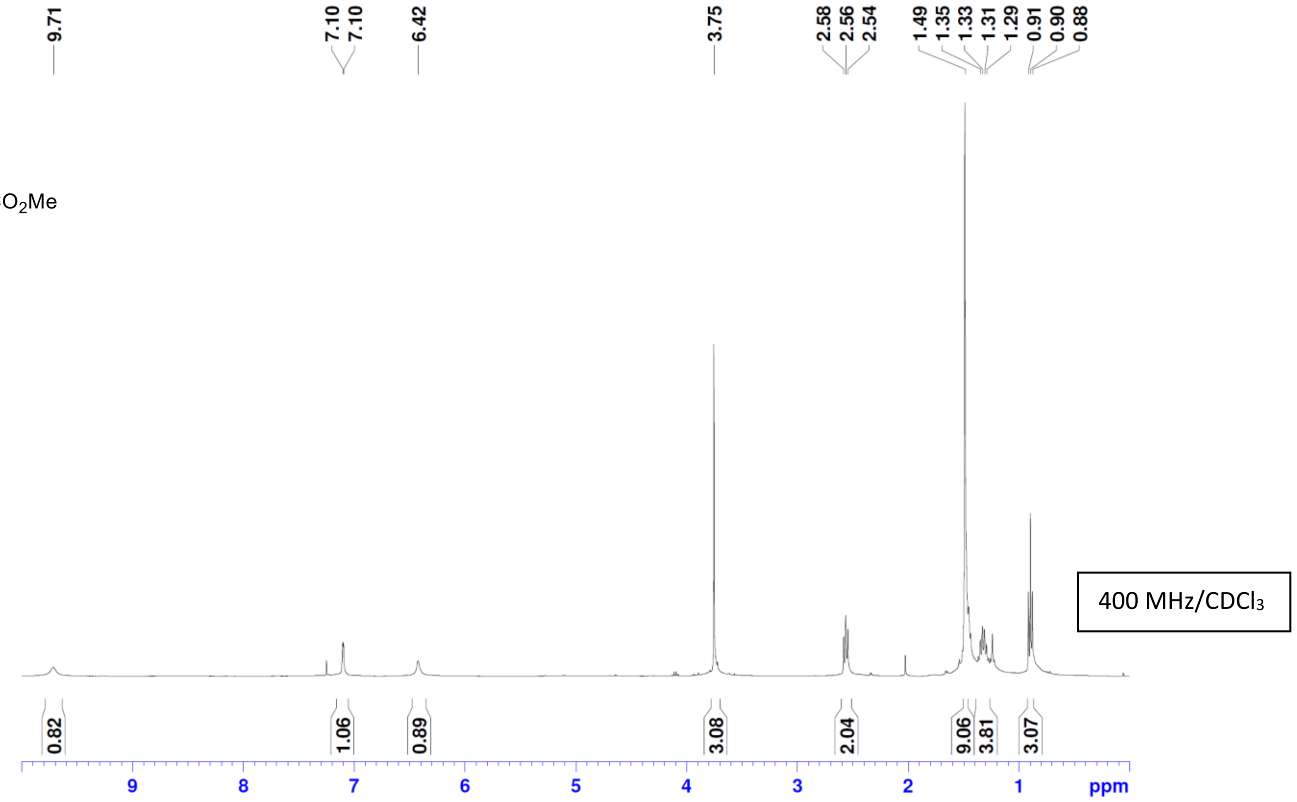
9.71
7.10
7.10
6.42
3.75
2.58
2.56
2.54
1.49
1.35
1.33
1.31
1.29
0.91
0.90
0.88
400 MHz/CDCl3
0.82
1.06
0.89
3.08
2.04
9.06
3.81
3.07
9
8
7
6
5
4
3
2
1
ppm

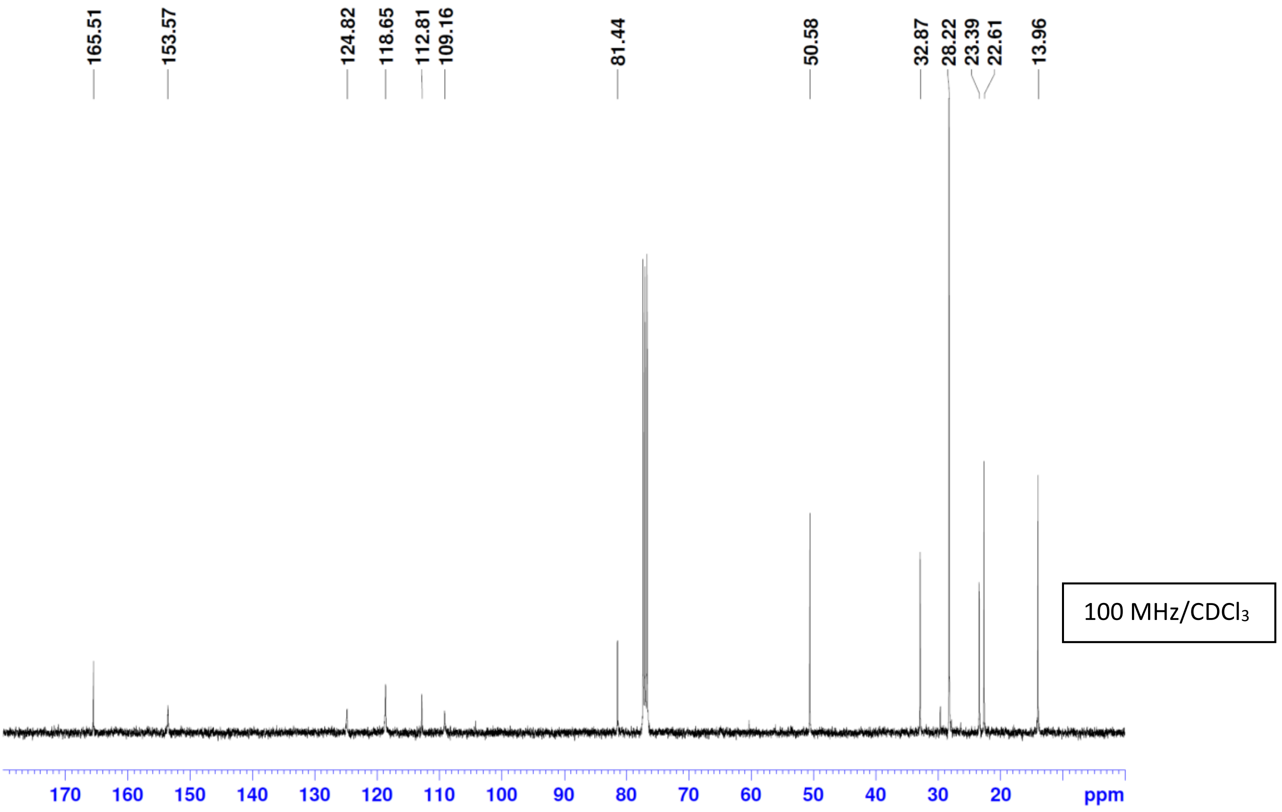
165.51
153.57
124.82
118.65
112.81
109.16
81.44
50.58
32.87
28.22
23.39
22.61
13.96
100 MHz/CDCl3
170
160
150
140
130
120
110
100
90
80
70
60
50
40
30
20
ppm

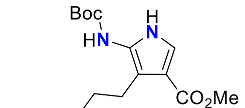
Boc
HN
H
N
CO2Me

12c

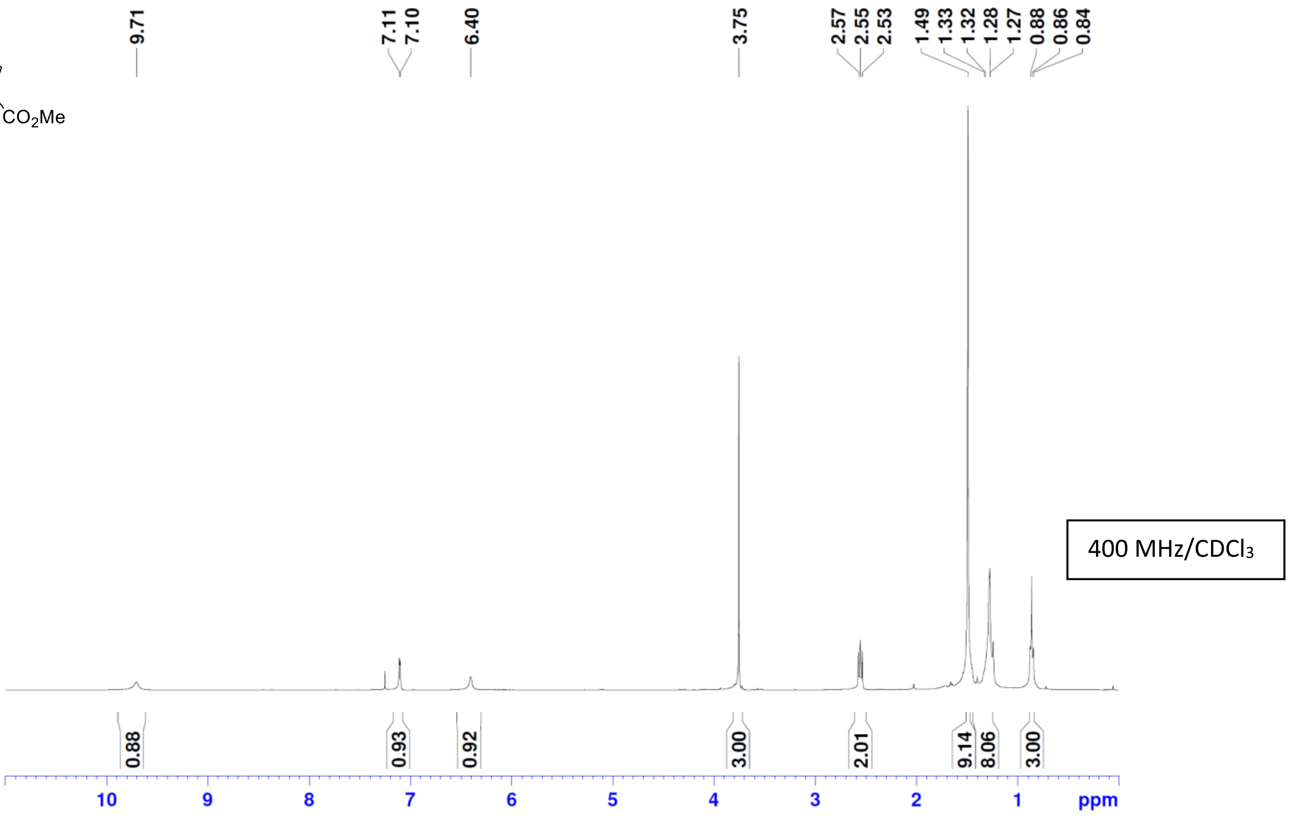
9.71
7.11
7.10
6.40
3.75
2.57
2.55
2.53
1.49
1.33
1.32
1.28
1.27
0.88
0.86
0.84
400 MHz/CDCl3
0.88
0.93
0.92
3.00
2.01
9.14
8.06
3.00
10 9 8 7 6 5 4 3 2 1 ppm

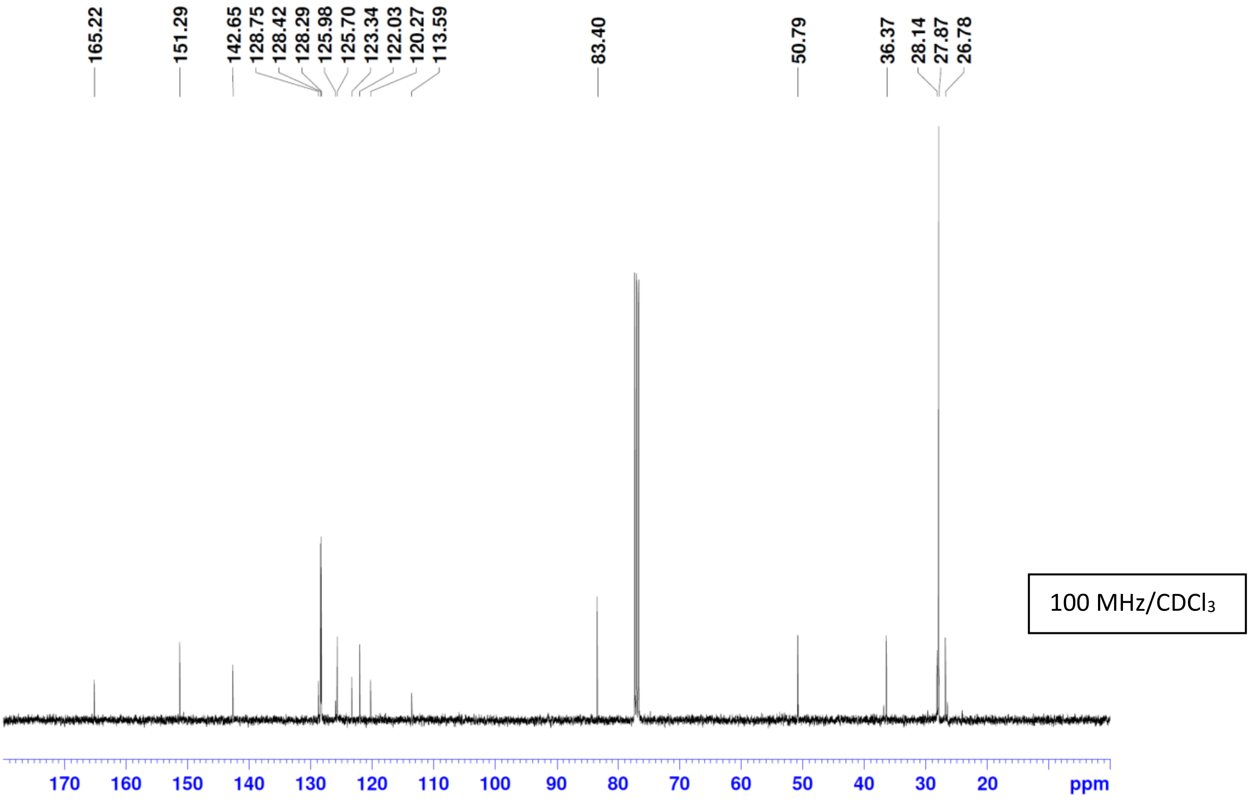
165.22
151.29
142.65
128.75
128.42
128.29
125.98
125.70
123.34
122.03
120.27
113.59
83.40
50.79
36.37
28.14
27.87
26.78
100 MHz/CDCl3
170 160 150 140 130 120 110 100 90 80 70 60 50 40 30 20 ppm

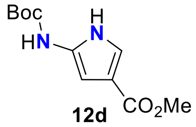
Boc
HN
H
N
CO2Me

12d

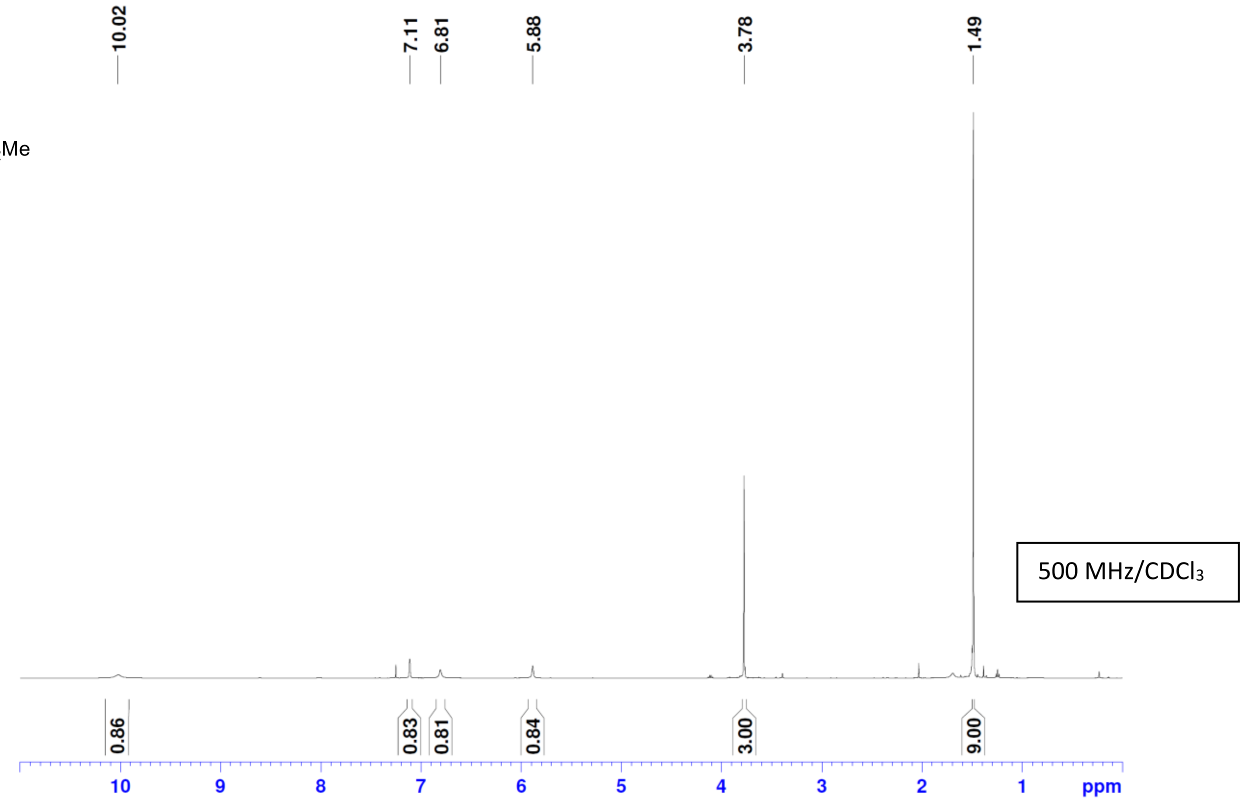
10.02
7.11
6.81
5.88
3.78
1.49
500 MHz/CDCl3
0.86
0.83
0.81
0.84
3.00
9.00
10 9 8 7 6 5 4 3 2 1 ppm

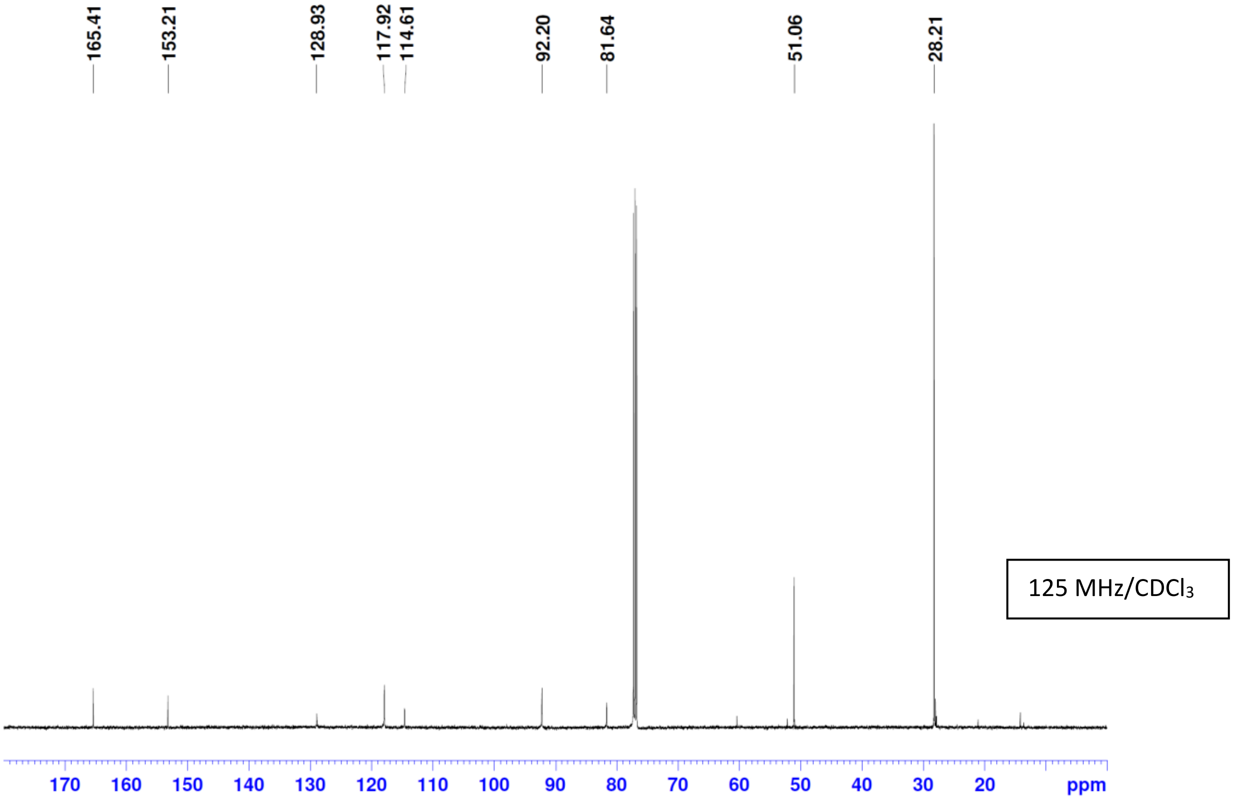
165.41
153.21
128.93
117.92
114.61
92.20
81.64
51.06
28.21
125 MHz/CDCl3
170 160 150 140 130 120 110 100 90 80 70 60 50 40 30 20 ppm

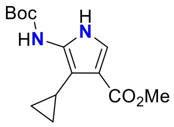

12e

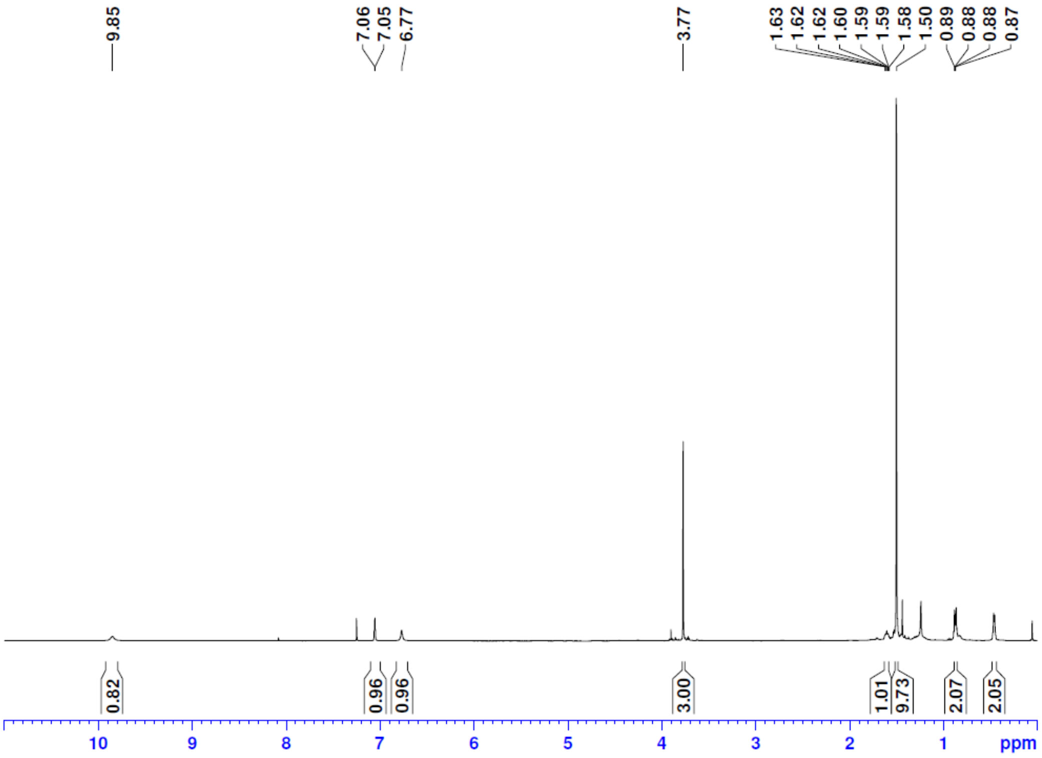

400 MHz/$CDCl_3$

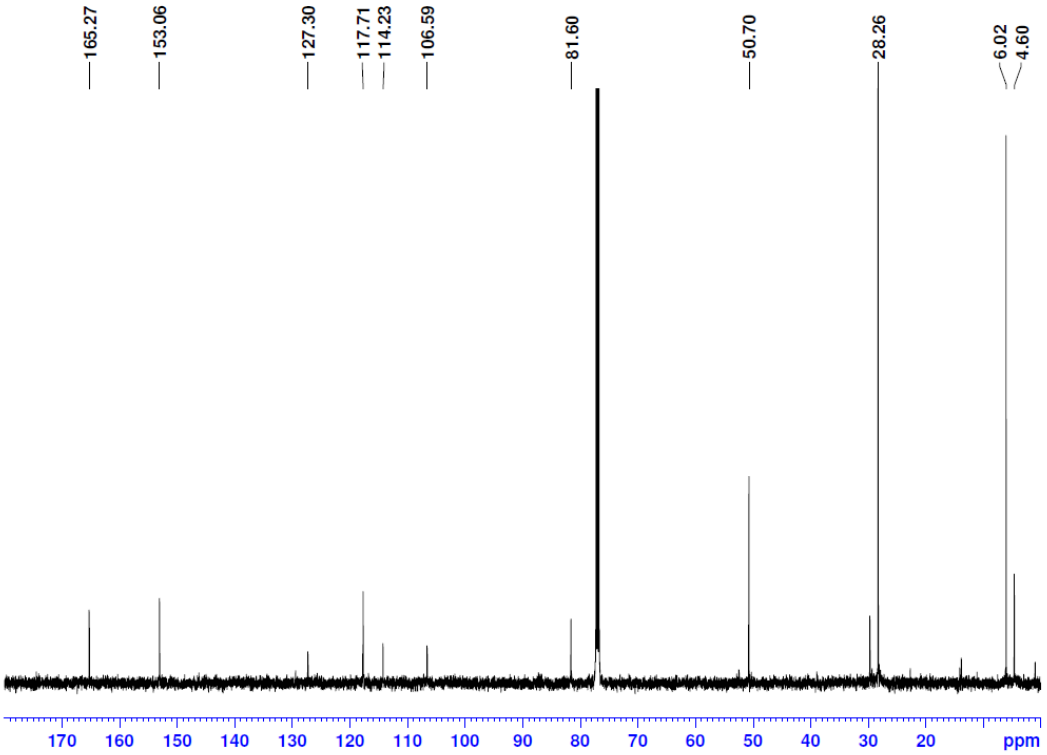

100 MHz/$CDCl_3$

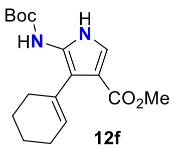
Boc
HN
H
N
CO2Me

12f

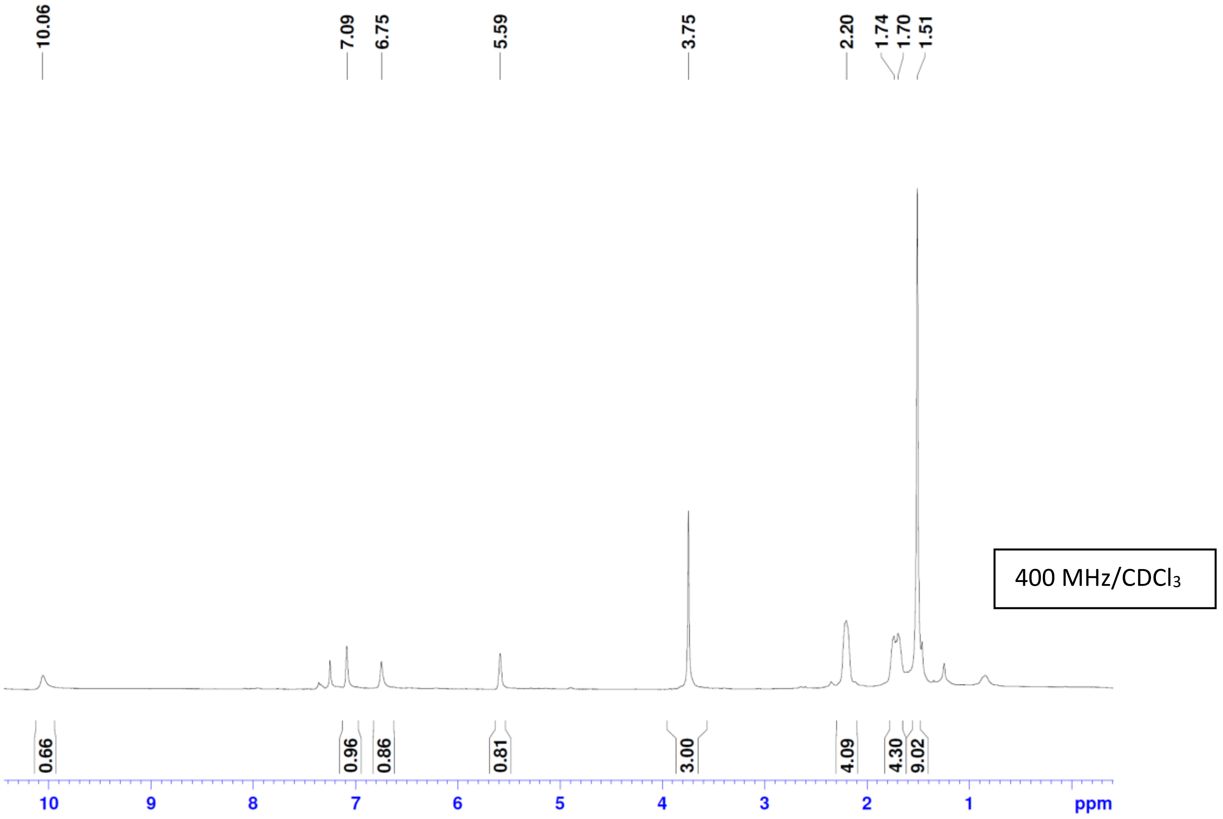
10.06
7.09
6.75
5.59
3.75
2.20
1.74
1.70
1.51
400 MHz/CDCl3
0.66
0.96
0.86
0.81
3.00
4.09
4.30
9.02
ppm

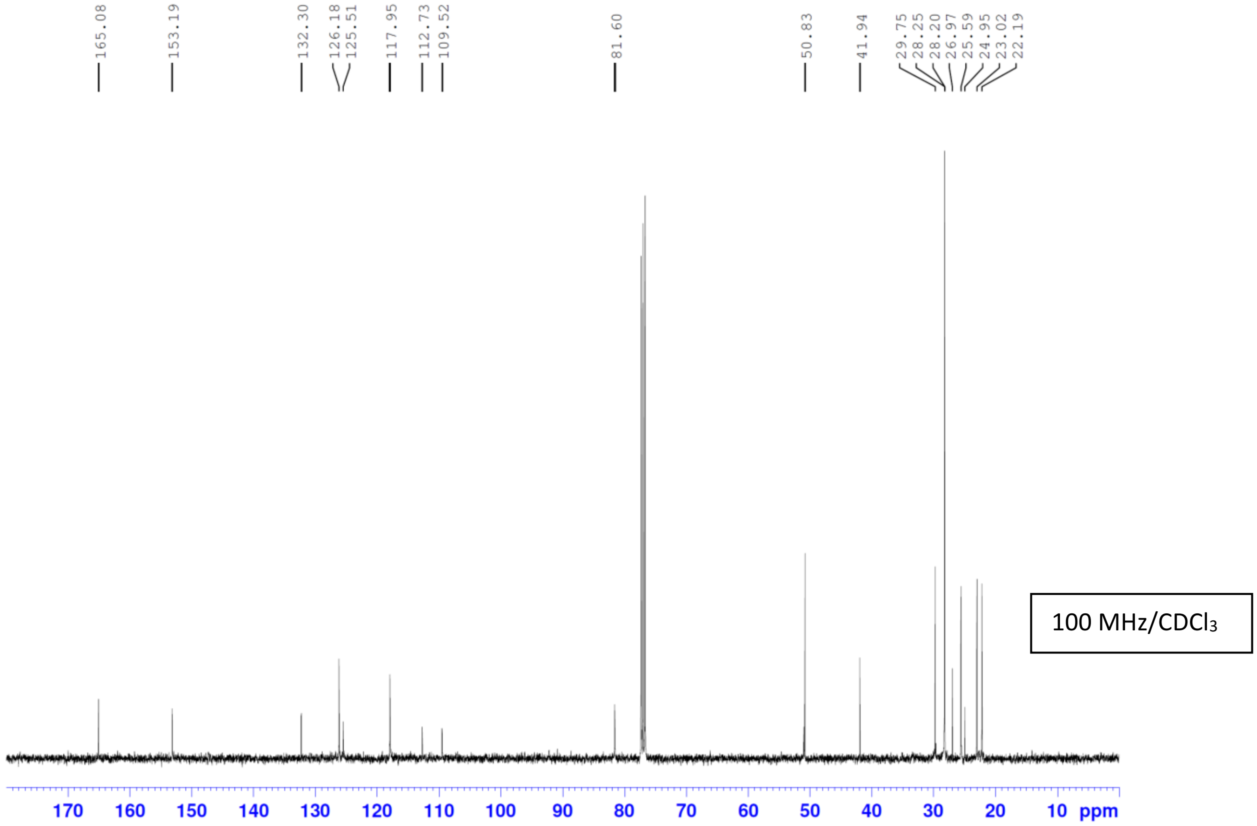
165.08
153.19
132.30
126.18
125.51
117.95
112.73
109.52
81.60
50.83
41.94
29.75
28.25
28.20
26.97
25.59
24.95
23.02
22.19
100 MHz/CDCl3
ppm

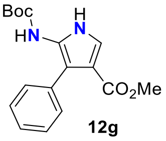
Boc
HN
H
N
CO2Me

12g

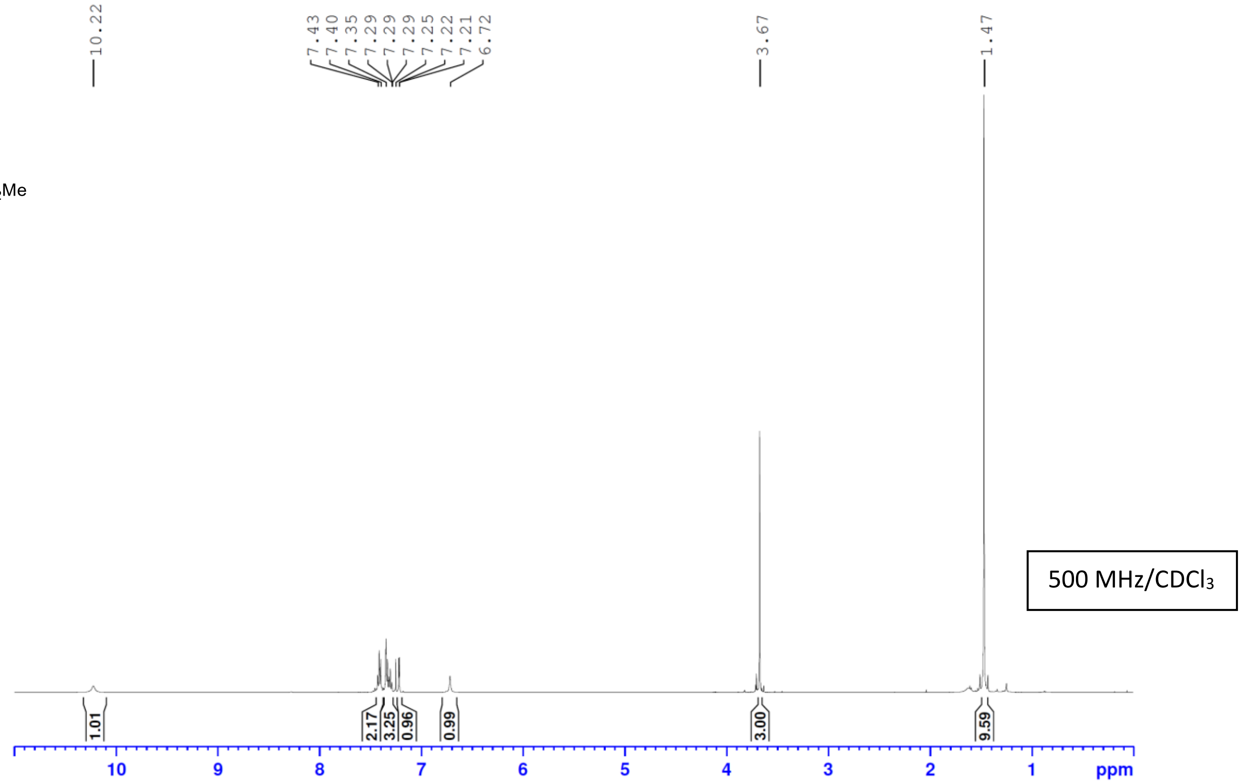
10.22
7.43
7.40
7.35
7.29
7.29
7.29
7.25
7.22
7.21
6.72
3.67
1.47
1.01
2.17
3.25
0.96
0.99
3.00
9.59
10 9 8 7 6 5 4 3 2 1 ppm
500 MHz/CDCl3

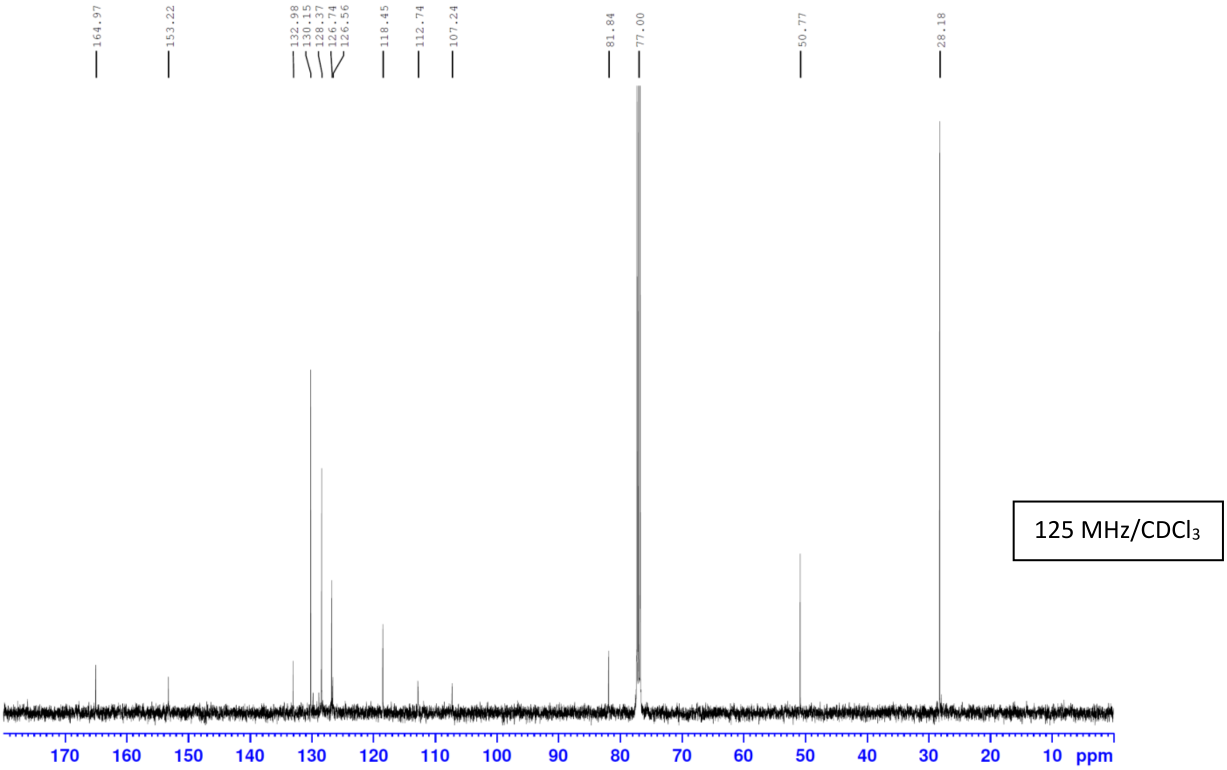
164.97
153.22
132.98
130.15
128.37
126.74
126.56
118.45
112.74
107.24
81.84
77.00
50.77
28.18
170 160 150 140 130 120 110 100 90 80 70 60 50 40 30 20 10 ppm
125 MHz/CDCl3

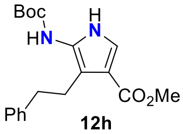
Boc
HN
H
N
CO2Me
Ph

12h

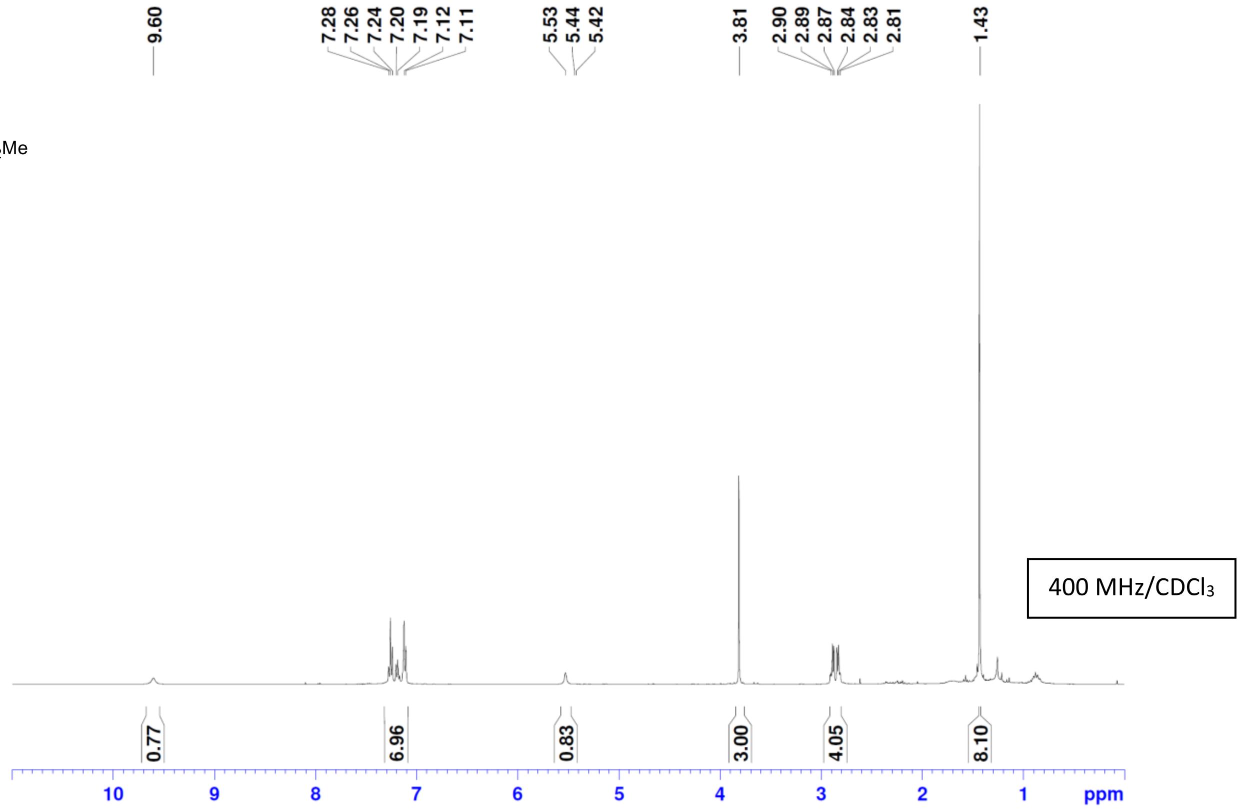
9.60
7.28
7.26
7.24
7.20
7.19
7.12
7.11
5.53
5.44
5.42
3.81
2.90
2.89
2.87
2.84
2.83
2.81
1.43
400 MHz/CDCl3
0.77
6.96
0.83
3.00
4.05
8.10
10 9 8 7 6 5 4 3 2 1 ppm

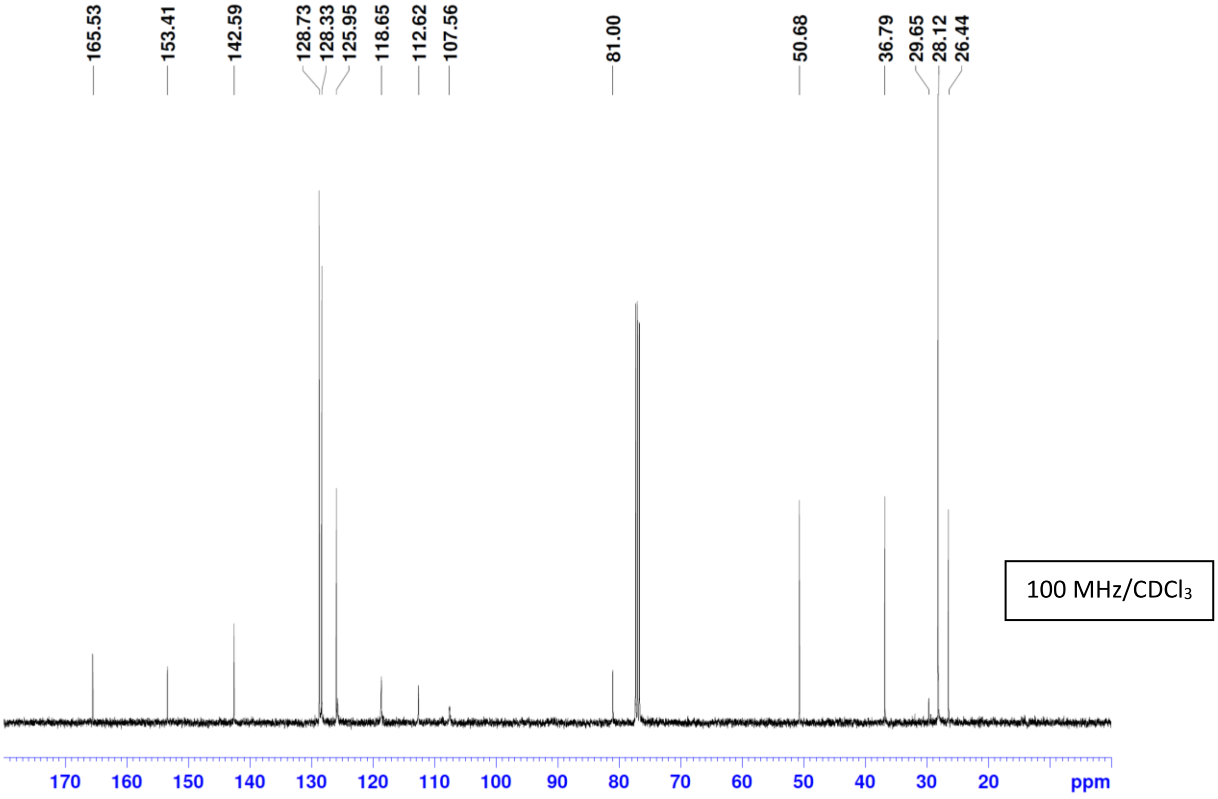
165.53
153.41
142.59
128.73
128.33
125.95
118.65
112.62
107.56
81.00
50.68
36.79
29.65
28.12
26.44
100 MHz/CDCl3
170 160 150 140 130 120 110 100 90 80 70 60 50 40 30 20 ppm

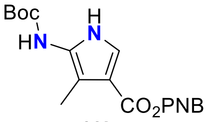

12i

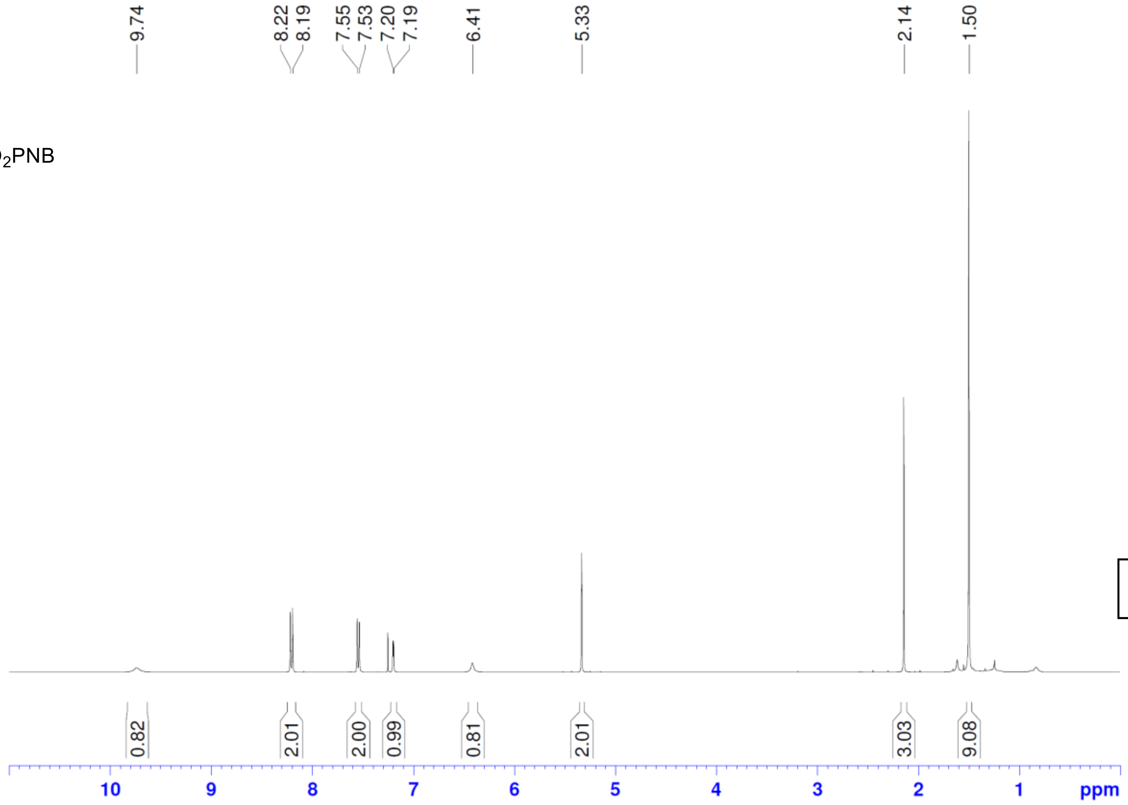

400 MHz/$CDCl_3$

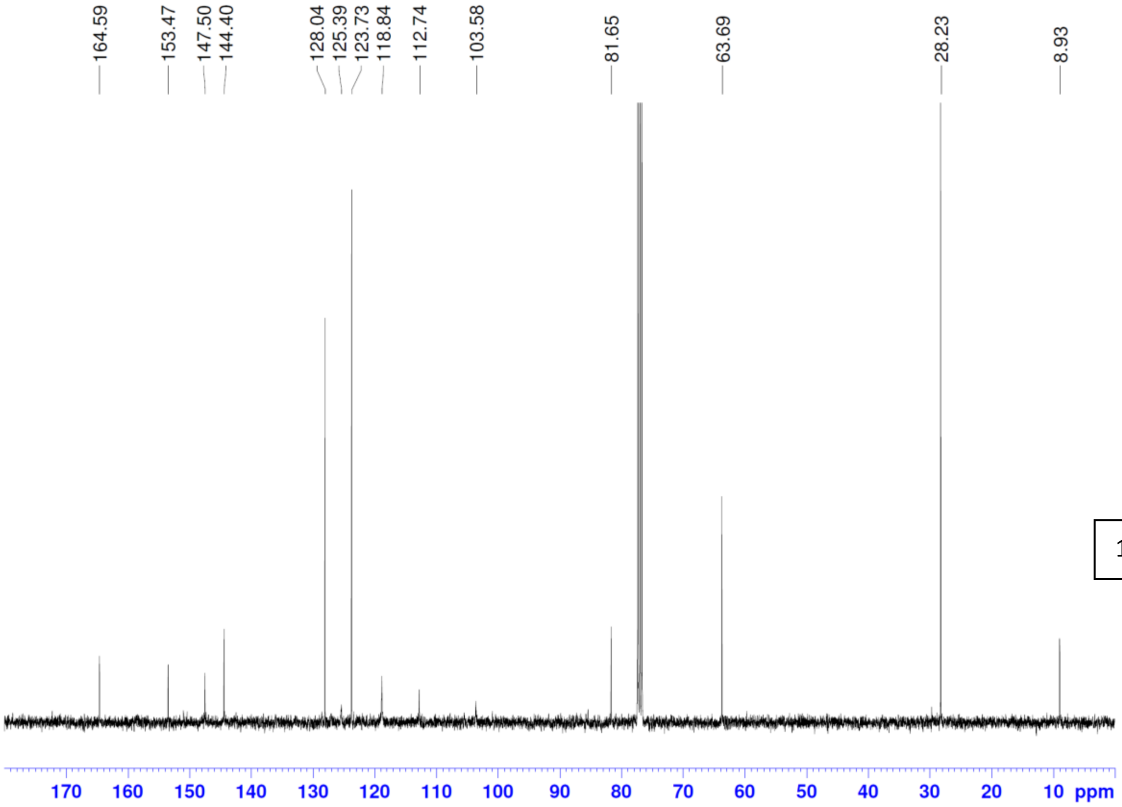

100 MHz/$CDCl_3$

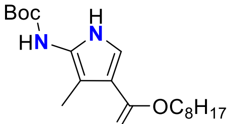

12j

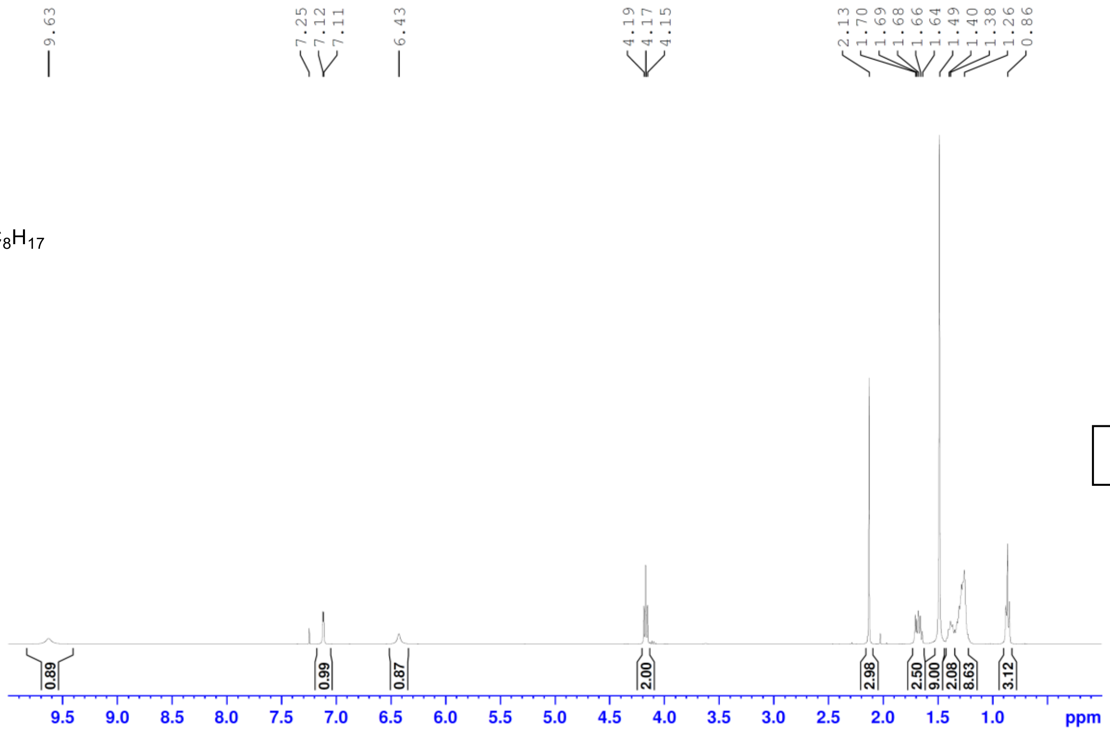

400 MHz/$CDCl_3$

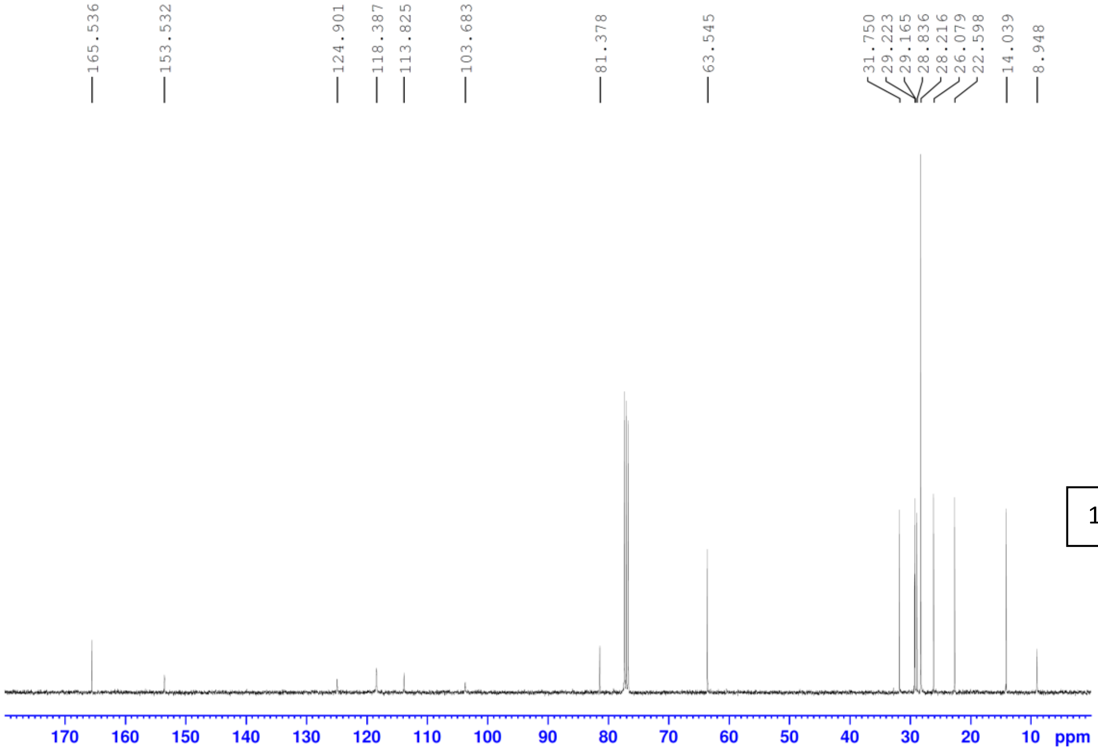

100 MHz/$CDCl_3$

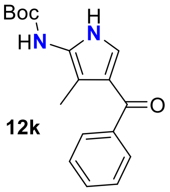

12k

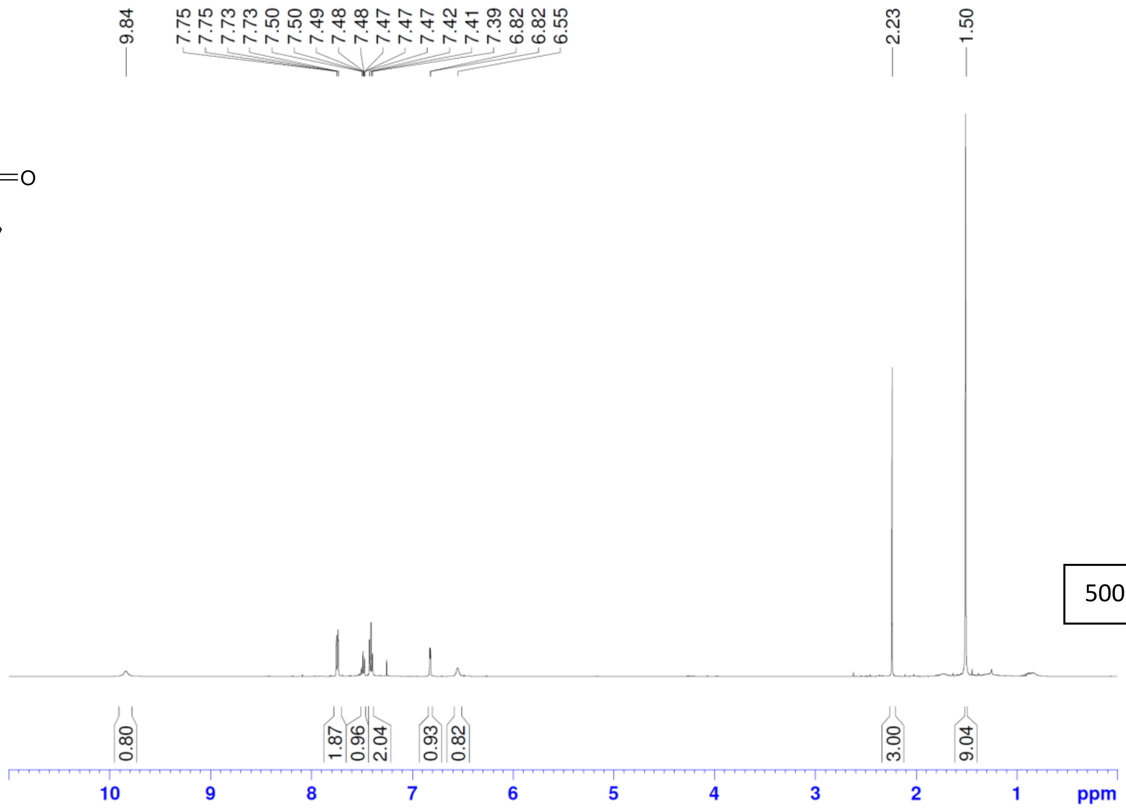

500 MHz/$CDCl_3$

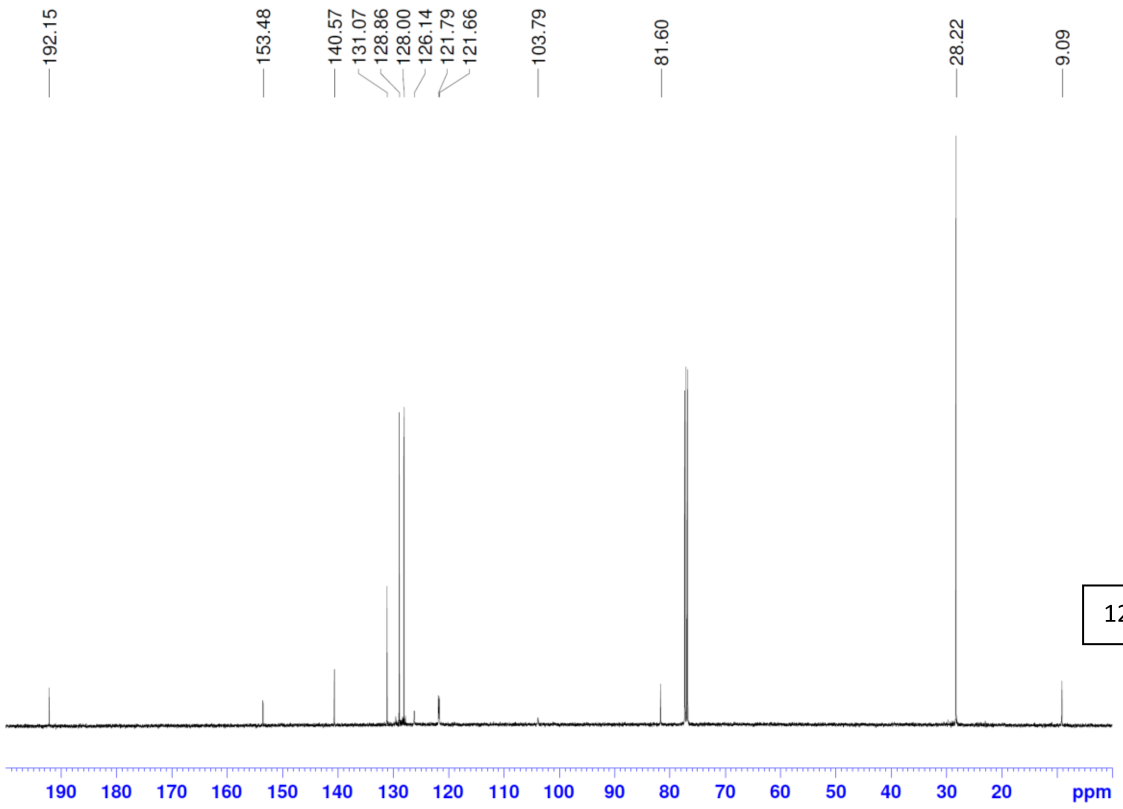

125 MHz/$CDCl_3$

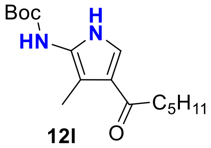

12l

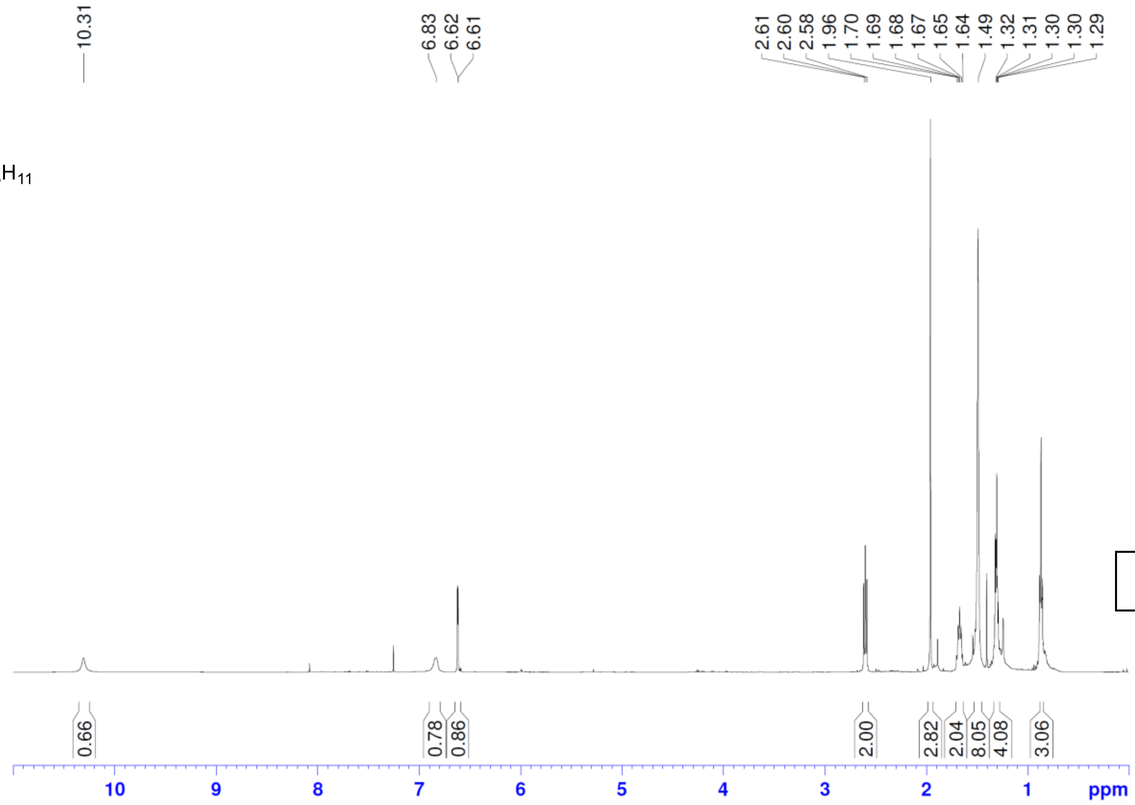

500 MHz/CDCl$_3$

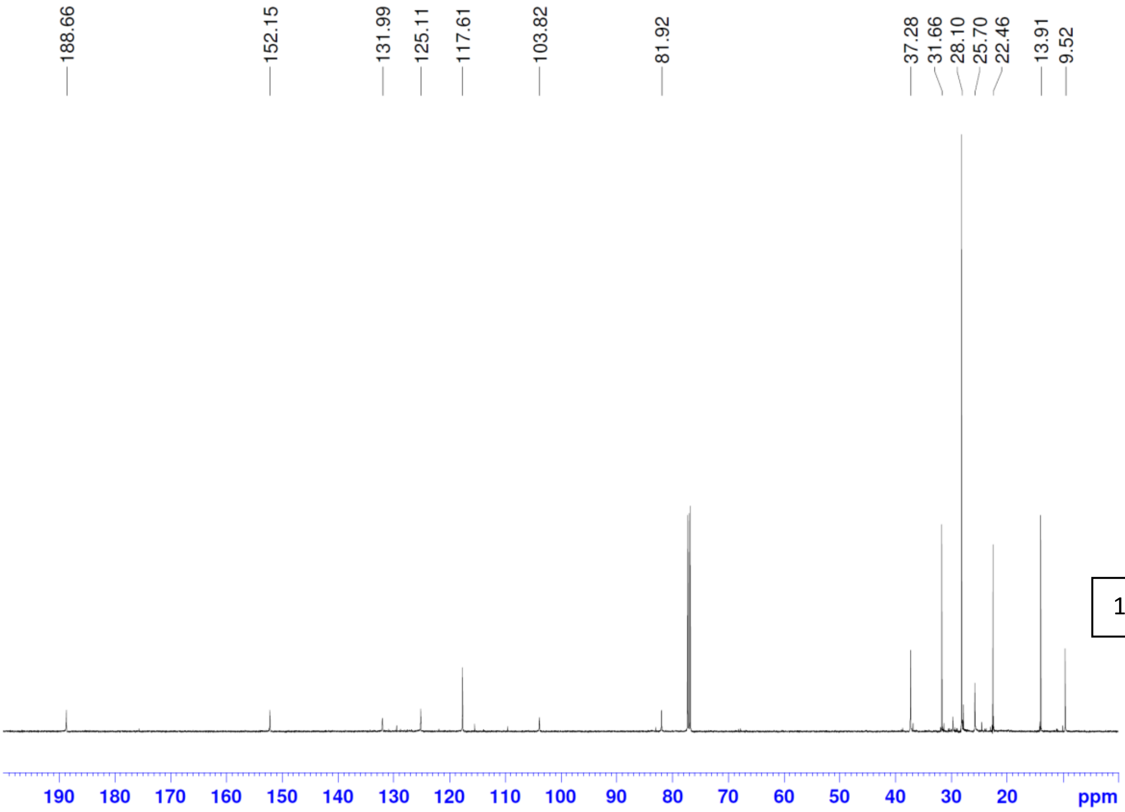

125 MHz/CDCl$_3$

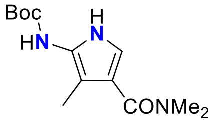
Boc
HN
H
N
CONMe$_2$

12m

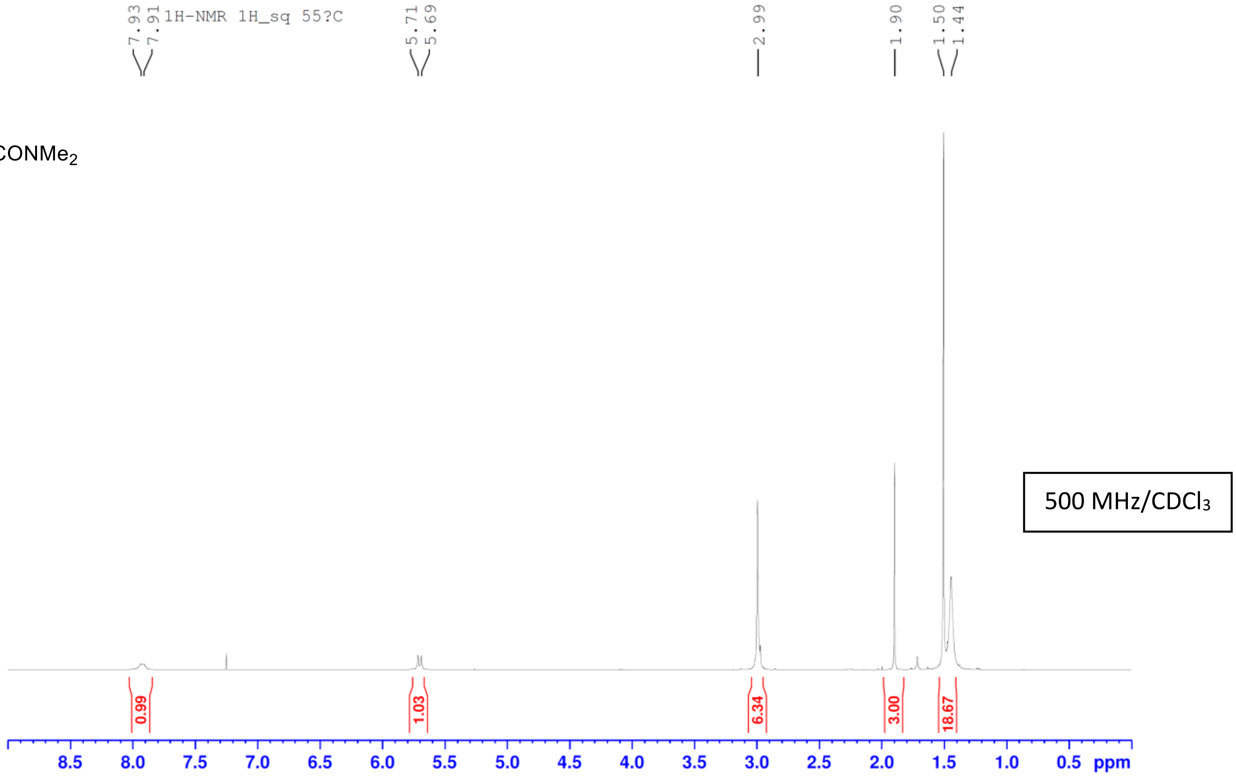
7.93
7.91
1H-NMR 1H_sq 55?C
5.71
5.69
2.99
1.90
1.50
1.44
500 MHz/CDCl$_3$
0.99
1.03
6.34
3.00
18.67
8.5 8.0 7.5 7.0 6.5 6.0 5.5 5.0 4.5 4.0 3.5 3.0 2.5 2.0 1.5 1.0 0.5 ppm

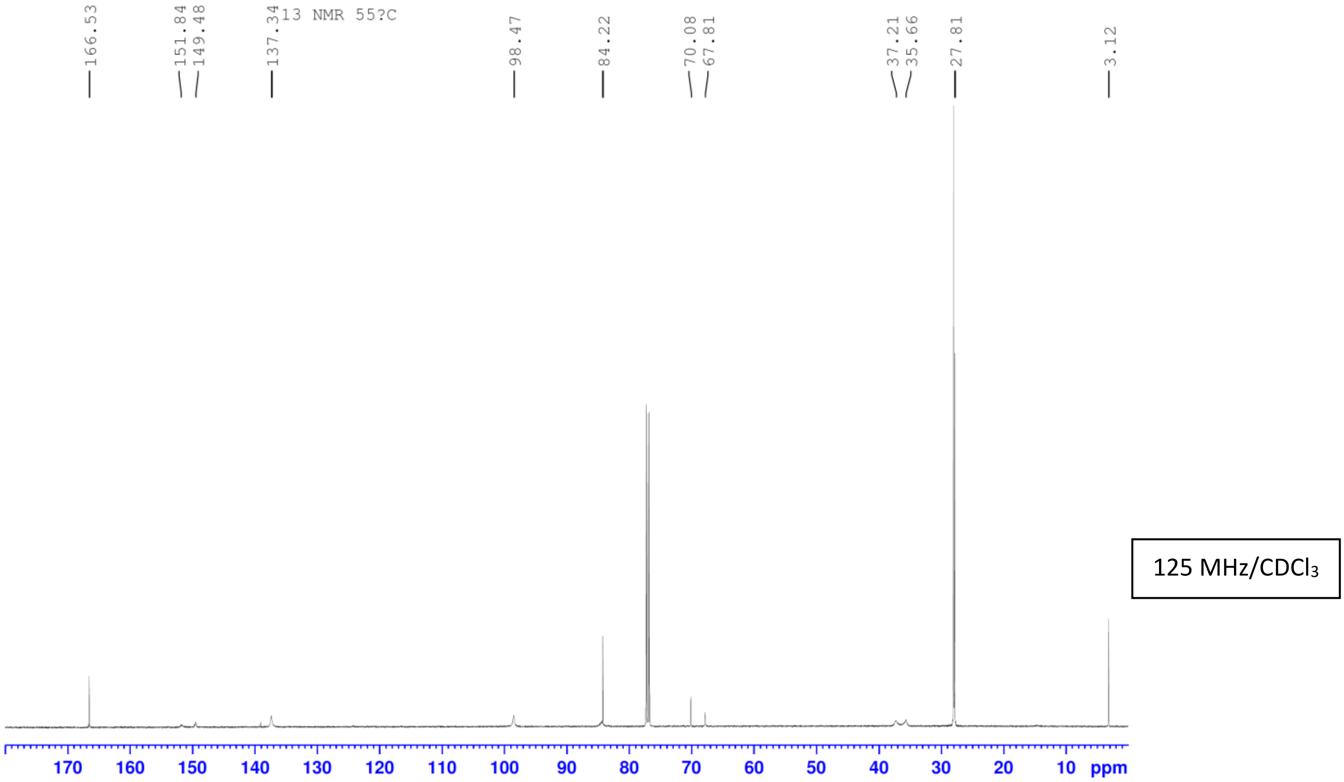
166.53
151.84
149.48
137.34
13 NMR 55?C
98.47
84.22
70.08
67.81
37.21
35.66
27.81
3.12
125 MHz/CDCl$_3$
170 160 150 140 130 120 110 100 90 80 70 60 50 40 30 20 10 ppm
